# Protective effect of paeoniflorin in diabetic nephropathy: A preclinical systematic review revealing the mechanism of action

**DOI:** 10.1371/journal.pone.0282275

**Published:** 2023-09-21

**Authors:** Xue-Er Zhang, Yao-bin Pang, Qu Bo, Shuang-Yuan Hu, Ju-Yi Xiang, Zheng-Ru Yang, Xiao-Mei Zhang, An-Jing Chen, Jin-Hao Zeng, Xiao Ma, Jing Guo

**Affiliations:** 1 Department of Dermatology, Hospital of Chengdu University of Traditional Chinese Medicine, Chengdu, China; 2 Department of Nephropathy, Hospital of Chengdu University of Traditional Chinese Medicine, Chengdu, China; 3 TCM Regulating Metabolic Diseases Key Laboratory of Sichuan Province, Hospital of Chengdu University of Traditional Chinese Medicine, Chengdu, China; 4 Institute of Medicinal Chemistry of Chinese Medicine, Chongqing Academy of Chinese Materia Medica, Chongqing, China; 5 State Key Laboratory of Southwestern Chinese Medicine Resources, School of Pharmacy, Chengdu University of Traditional Chinese Medicine, Chengdu, China; Foshan University, CHINA

## Abstract

**Background:**

Paeoniflorin (PF), the main active glucoside of Paeonia Lactiflora, has many pharmacological activities, such as inhibition of vasodilation, hypoglycemia, and immunomodulation. Although the current evidence has suggested the therapeutic effects of PF on diabetic nephropathy (DN), its potential mechanism of action is still unclear.

**Purpose:**

A systematic review and meta-analysis of the existing literature on paeoniflorin treatment in DN animal models was performed to evaluate the efficacy and mechanism of PF in DN animal models.

**Methods:**

The risk of bias in each study was judged using the CAMARADES 10-item quality checklist with the number of criteria met varying from 4 / 10 to 7 / 10, with an average of 5.44. From inception to July 2022, We searched eight databases. We used the Cochrane Collaboration’s 10-item checklist and RevMan 5.3 software to assess the risk of bias and analyze the data. Three-dimensional dose/time-effect analyses were conducted to examine the dosage/time-response relations between PF and DN.

**Results:**

Nine animal studies were systematically reviewed to evaluate the effectiveness of PF in improving animal models of DN. Meta-analysis data and intergroup comparisons indicated that PF slowed the index of mesangial expansion and tubulointerstitial injury, 24-h urinary protein excretion rate, expression of anti-inflammatory mediators (mRNA of MCP-1, TNF-α, iNOS, and IL-1 β), and expression of immune downstream factors (P-IRAK1, TIRF, P-IRF3, MyD88, and NF-κBp-p65). Furthermore, modeling methods, animal species, treatment duration, thickness of tissue sections during the experiment, and experimental procedures were subjected to subgroup analyses.

**Conclusion:**

The present study demonstrated that the reno-protective effects of PF were associated with its inhibition on macrophage infiltration, reduction of inflammatory mediators, and immunomodulatory effects. In conclusion, PF can effectively slow down the progression of DN and hold promise as a protective drug for the treatment of DN. Due to the low bioavailability of PF, further studies on renal histology in animals are urgently needed. We therefore recommend an active exploration of the dose and therapeutic time frame of PF in the clinic and in animals. Moreover, it is suggested to actively explore methods to improve the bioavailability of PF to expand the application of PF in the clinic.

## 1. Introduction

Diabetic nephropathy (DN) is a leading cause of end-stage renal disease (ESRD). DN occurs in approximately 30% of patients with type 1 diabetes mellitus (T1DM) and approximately 40% of patients with type 2 diabetes mellitus (T2DM), representing a diabetic microvascular complication caused by glomerulopathy and tubulopathy [1, 2]. The typical presenting features of DN are early hyperfiltration and albuminuria (glomerular filtration rate (GFR) below 60 ml / min /1.73m^2^ or albumin-to-creatinine ratio (ACR) higher than 30 mg / g for more than 3 months) and then the gradual decline of renal function [3]. The pathogenesis of DN is complex and diverse, mainly involving changes in renal hemodynamics and renal structure (characterized by glomerular hypertrophy, inflammation, and fibrosis). Moreover, DN is closely related to the dysfunction of the angiotensin aldosterone system and immune system. Studies have shown that the intrarenal inflammation mediated by macrophage activation and toll-like receptors (TLRs) plays an important role in the development and progression of DN [4]. Silent information regulator 1 (SIRT1) / nuclear factor related factor 2 (Nrf2) / nuclear factor-κB (NF-κB) signaling pathways are closely related to inflammation and oxidative stress [5]. Hence, the SIRT1/Nrf2/NF-κB signal may become a potential therapeutic target to inhibit oxidative stress and inflammation, thereby exerting reno-protective effects in DN.At present, few few therapeutic agents directly target the renal and vascular complications of diabetes, and most studies focus on albuminuria. However, the association between albuminuria and renal function decline may not be significant, because clinically, some diabetic patients with renal insufficiency still show albuminuria in the normal range [6]. The prevalence of normoalbuminuric diabetes mellitus (DADN) with renal dysfunction is gradually increasing and has become the prevalent phenotype of DN [7]. Therefore, it is necessary to seek novel downstream bioindicators and effective treatments to delay DN progression.

Paeoniflorin (PF) is the most abundant component (> 40%) of the total glucoside of peony (TGP), and also the main bioactive component. The plant extract TGP is a mixture of glycosides isolated from the roots of the traditional herb Paeonia Lactiflora Pall [8]. Mechanistically, PF can regulate the function of immune cells and endothelial cells (ECS), reduce the production of inflammatory mediators, and restore abnormal signal transduction [9]. PF has shown notable efficacy in treating rheumatoid arthritis, psoriasis, systemic lupus erythematosus, and diabetes [10–13].Importantly, PF has been reported to inhibit high glucose-induced macrophage activation and renal inflammation [14], ameliorate glycemic variability-induced oxidative stress [15], and improve the renal biochemical and pathological damage [16]. Although the existing evidence shows that PF has a therapeutic effect on DN, its potential mechanism of action has not been fully clarified. Herein, this study aimed to conduct a systematic review and meta-analysis of the existing literature on PF treatment in DN animal models to evaluate the efficacy and mechanism of PF in DN animal models.

## 2. Methods

### 2.1 Data sources and search strategies

We performed literature retrieval from Chinese Science and Technology Journal Database, Chinese Biomedical Database, Wan Fang, China National Knowledge Infrastructure, PubMed, Cochrane Library, EMBASE, and Web of Science from inception to July 2022 to identify animal experimental studies that met the targeted PF treatment for DN. To obtain a complete literature list, we carefully searched for all eligible studies. The search terms used in PubMed were as follows: “Diabetic Nephropathy”, “Diabetic Kidney Diseases”, “Kidney Diseases, Diabetic”, “Intracapillary Glomerulosclerosis”, “Glomerulosclerosis, Nodular”, “Paeoniflorin”, “Paeoniflorin-6’-O-benzene sulfonate” and “Paeoniflorin sulfonate”. The specific search methods are summarized in Appendix 1 in S1 File.

### 2.2 Eligibility criteria

The selection criteria were as follows: (1) Population (P): establishment of DN rodent models in a generally accepted manner; (2) Intervention (I) and Control (C): the experimental group received treatment with PF single agent or derivative at any dose, and the model group received treatment with an equal amount of nonfunctional substances (normal saline) or did not receive treatment; (3) Results (R): the primary outcome measures of the study were changes in histopathology, morphology, and renal function parameters, including 24-h urinary protein excretion rate, etc., while the reno-protective mechanism of PF against DN was selected as the secondary outcome measures, including the changes in metabolic parameters, biochemical parameters, and inflammatory and oxidative stress markers.

The exclusion criteria were as follows: (1) Population (P): the target disease was not DN (no DN pattern); (2) Intervention (I): PF-based prescription or combination therapy with other drugs; (3) Control (C): unclear comparison with other drugs (e.g., Western pharmaceuticals, combination therapies from traditional Chinese medicine); (4) Outcome (O): no predefined outcome index or no data available; (5) Study design and format: non in vivo studies (in vitro studies, clinical trials, review articles, case reports, meta-analyses, reviews, commentaries, abstracts, editorials, multiple publications, or patents). Notably, although a variety of Paeonia species have been widely used in folk medicine, the extracts of these plants have not been used in clinical trials alone currently, but are often used as part of a prescription. However, the combined application of multiple traditional Chinese medicines in a prescription does not seem to prove the medicinal value of a single herb. Therefore, we excluded clinical trials that included PF compounds. Detailed characteristics of the inclusion and exclusion criteria are shown in Table 1.

**Table 1:** Literature inclusion and exclusion criteria.

	Inclusion criteria	Exclusion criteria
Population (P)	Establishment of DN rodent models in a generally accepted manner	The target disease was not DN (no DN pattern)
Intervention (I)	The experimental group received treatment with PF single agent or derivative at any dose. The model group received treatment with an equal amount of nonfunctional substances (normal saline) or did not receive treatment	PF-based prescription or combination therapy with other drugs
Control (C)		Unclear comparison with other drugs (Western pharmaceuticals, combination therapies from traditional Chinese medicine)
Outcome (O)	Main outcome measures: changes in histopathology, morphology, and renal function parameters (24-hour urinary protein excretion rate). Secondary outcome measures: metabolic parameters, biochemical parameters, and inflammatory and oxidative stress markers.	No predefined outcome index or no available data
Study design and format	Vivo studies	Non in vivo studies

### 2.3 Data extraction

Two trained and qualified researchers (ZY and JX) performed literature screening and data extraction independently in strict accordance with the inclusion/exclusion criteria. Any disagreement was resolved by the third party (JG). All eligible articles were independently evaluated from the following aspects: (1) The year of publication and the name of the first author; (2) Details of animals (quantity, species, sex, and weight); (3) The method of establishing animal model and the standard of successful modeling; (4) The use of anesthetics in the process of the experiment; (5) Treatment group and control group; (6) Primary and secondary outcomes and intergroup differences. If the results were shown by gradient drug doses or multiple time points, only the final measurement data for the highest dose of drugs were used. If the documentation data were in the form of graphs, we attempted to obtain the data from the original author. If the raw data could not be acquired, the numerical values were measured from the graphs by using the publicly available Webplotdigitizer was used for measurement. The final results were presented graphically, not in digital text. The remaining 62 studies.

### 2.4 Risk of bias in individual study

Two qualified researchers (BQ and ZY) assessed the risk of bias for each included study using the CAMARADES 10-item quality checklist [17] with minor modifications: A: peer-reviewed publication; B: control of laboratory temperature; C: random allocation to treatment or control; D: blinded modeling (modelling by randomisation or use of transgenic knockout mice); E: blinded assessment of outcomes; F: use of anaesthetics without nephroprotective activity or nephrotoxicity; G: selection of a reasonable animal model (old age, hyperlipidemia, or hypertension); H: sample size calculation; I: compliance with animal experimental welfare regulations (≥ three of the following: nutrition, disinfection, ambient temperature and humidity, preoperative anesthesia, postoperative analgesia, and finally euthanasia); J: declaration of potential conflicts of interest. Each study received a maximum quality score of 10 points, and the median of the calculated results was taken. Any disagreement during data extraction and quality assessment was resolved by consensus or third arbitration between the authors (JG and JZ).

### 2.5 Statistical analysis

We downloaded and used Revman 5.3 software on the official website to perform statistical analysis on the extracted data. Considering the differences in measurement instrument and animal modeling, we quantitatively determined the summary statistics of the results using the standardized mean difference (SMD) with 95% confidence interval (95% CI). A value of *p* < 0.05 indicated that the difference between the experimental and model groups was statistically significant. The heterogeneity between studies was assessed using the I^2^ statistical test. If *I*^*2*^ ≤ 50%, it was indicative of no significant heterogeneity, and a fixed effects model was employed to combine effect sizes. If *I*^*2*^ > 50%, it was suggested that the included studies were of different quality, and a random effects model was adopted, or sensitivity analyses were performed. We performed subgroup analyses for modeling methods, animal species, PF doses, and treatment duration to explore the sources of heterogeneity. In addition, time dose interval analysis was performed using Origin 2021 software.

## 3.Results

### 3.1 Research retrieval

**Fig 1:**
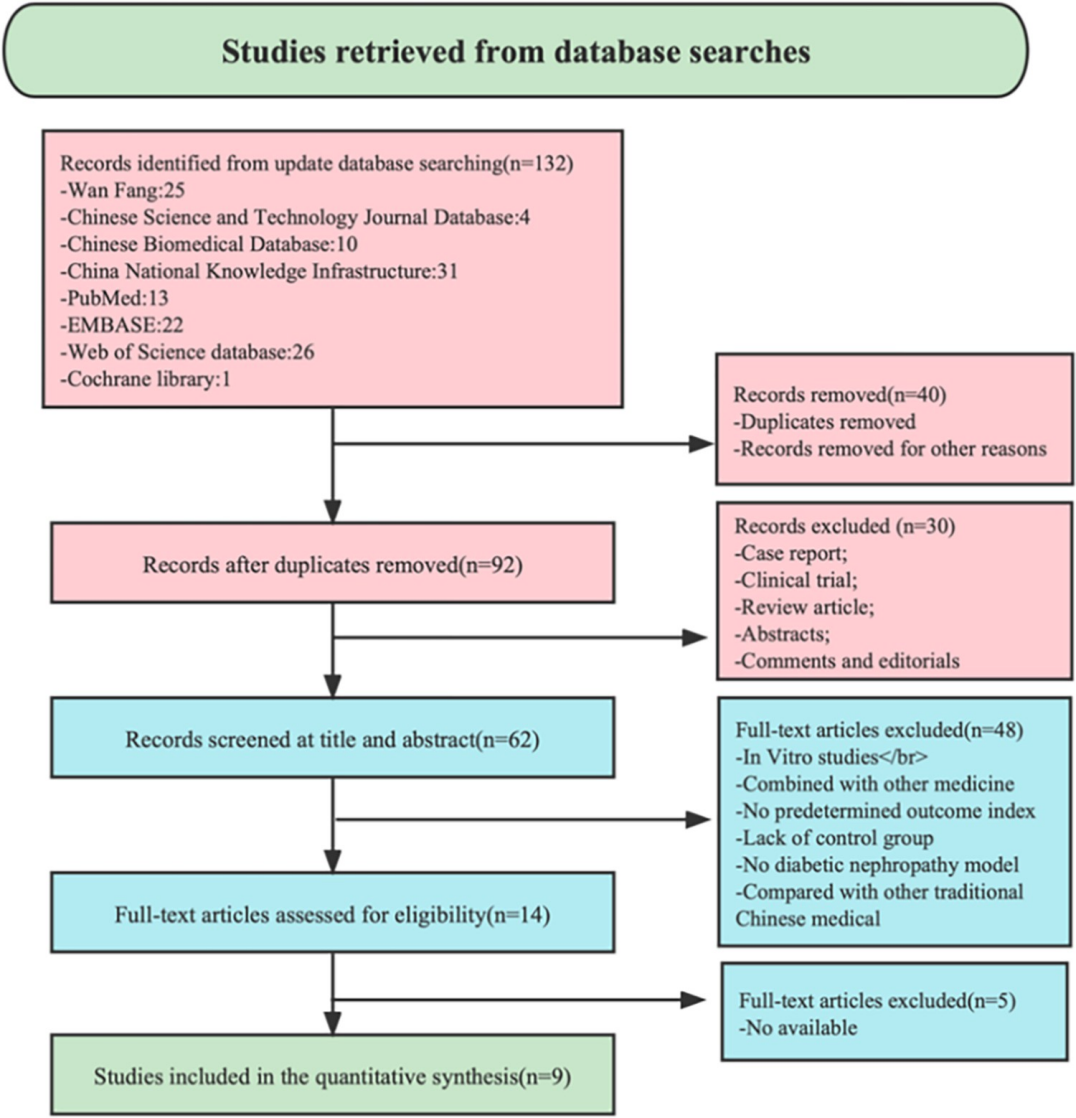
Summary of the process for identifying candidate studies.

We retrieved 132 potentially relevant studies from eight databases. After reviewing titles and abstracts, we excluded 60 duplicate or irrelevant studies. The remaining 62 studies were reviewed in full-text, 48 of which were not considered because they were not TCM monomer studies or did not include animal models. Finally we identified 9 eligible studies, including 5 studies in English [18–26]. The specific retrieval process is shown in Fig 1.

### 3.2 Characteristics of included studies

**Fig 2:**
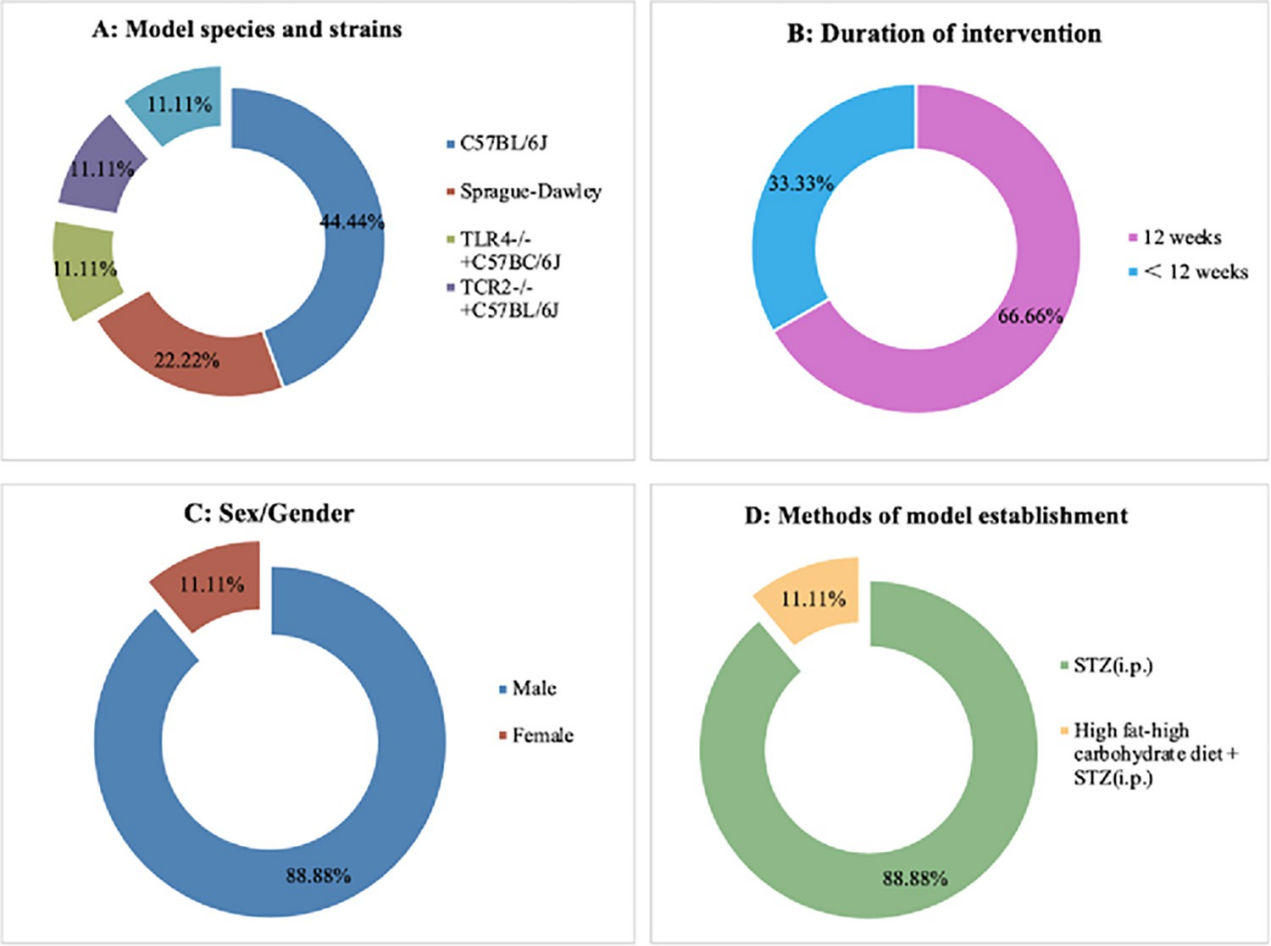
Study characteristics of eligible studies. Model species and strains(A); Duration of intervention(B);Sex/Gender(C);Methods of model establishment(D).

A total of 9 studies involving 526 animals were finally included. The sample size of each study varied from 8 to 12 animals. Male C57BL/6 mice were used in 6 studies; Sprague Dawley (SD) rats were used in 2 studies; male TLR4^-/-^ mice were used in 1 study and male TLR2^-/-^ mice were used in 1 study; male db/db mice were used in 1 study. Seven studies used spontaneous diabetic mutant or transgenic mice (1 study used db/db mice and 6 studies used C57BL/6 mice, of which 2 studies used male TLR4^-/-^ mice versus male TLR2^-/-^ mice, respectively). The weight of SD rats was 200–250 g, and the weight of mice was 18–20 g. Eight studies established DN models by intraperitoneal injection of streptozotocin (STZ); 1 study used a high-fat diet for several weeks and intraperitoneal injection of STZ. For the induction of anesthesia, 5 studies did not report anesthetics; 3 studies reported sodium pentobarbital and 1 study used CO_2_. PF details in each study are shown in Fig 2 and Table 2. Nine studies implemented gradient doses of PF from 5 to 100 mg • kg ^− 1^ • d ^− 1^ by oral or intragastric administration.

**Table 2:** Information of PF of each study.

Study(years)	specifications	Source	Purity (%)	Quality control reported
**(Yang2018)**	Powder	Beijing Zhongshan Jin qiao Biotechnology Co., Ltd	NM	?
**(Huang2020)**	Powder	the Chemistry Lab of the institute of Clinical Pharmacology of Anhui Medical University	NM	HPLC
**(Zhao2022)**	Powder	China Institute for Food and Drug Control.	NM	?
**(Duan2018)**	Powder	Nanjing Guanrun Biological Products Co., Ltd.	NM	HPLC-DAD, HPLC-ELSD
**(Fu2009)**	Powder	Xuan Cheng Baicao Plants Industry and Trade, Anhui, China.	95%	HPLC
**(Y. Shao 2017)**	Powder	Nanjing GOREN BIO Technology Co., Ltd. (Nanjing, China).	98.78%	HPLC
**(Zhang2017)**	Powder	Nanjing GOREN BIO Technology Co., Ltd (Nanjing, China).	98.78%	HPLC
**(Y. Shao 2019)**	Powder	Nanjing GOREN BIO Technology Co., Ltd. (Nanjing, China).	98.78%	HPLC
**(Li2018)**	Powder	Nanjing GOREN BIO Technology Co., Ltd. (Nanjing, China).	98.78%	HPLC

Note: Abbreviations (see Appendix 1 in S1 File);—Data not reported; NM: Not mentioned; p < 0.05 represents the statistical difference.

To assess the effect of PF treatment on DN, (1) renal pathology and 24-hour urinary protein were used as the primary outcomes in 7 studies; (2) secondary outcomes were as follows: 1) BG-related parameters: BG reported in 9 studies, glut reported in 1 study, FIN reported in 1 study, and HOMA-IR reported in 1 study; 2) kidney function measures: BUN reported in 2 studies, SCr reported in 1 study, Cr reported in 1 study, UAlb / UCR reported in 1 study, KW / BW ratio reported in 2 studies, and TGF-β1 reported in 1 study; 3) anti-inflammatory factor indicators and downstream factors: TNF-α in 6 studies, IL-1β in 6 studies, MCP-1 in 7 studies, IL-6 in 1 study, GRK2 in 1 study, and SIRT1 in 1 study; 4) indicators of macrophage activation: iNOS in 6 studies, CD68 in 6 studies, and ICAM1 in 1 study; 5) TLR2 versus TLR4 signaling pathways and protein expression: TLR2 in 3 studies, TLR4 in 2 studies, MyD88 in 4 studies, p-irs1 in 1 study, P-IRAK1 in 4 studies, P-IRF3 in 3 studies, and TRIF in 3 studies; 6) JAK2 / STAT3 signaling-related proteins: p-JAK2 in 2 studies, p-JAK2 / JAK2 in 2 studies, and p-STAT3 / STAT3 in 2 studies; 7) NF- κB signaling pathway proteins: NF-κB-p65 in 5 studies and NF-κBp-p65 in 4 studies; 8) oxidative stress indicators: SOD in 1 study, Nrf2 in 1 study, and GSH-PX in 1 study. Detailed characteristics of included studies are shown in Table 3.

**Table 3:** Characteristics of the included studies.

(Year of publication)	Species (Sex, n = experimental/ control group)	Weight	Modeling method	Criteria for successful modeling	Anesthetic	Treatment group (Method to astragal sides)	Control group	Outcome index (time)
**(Yang2018)**	C57BL/6J mice male (12/12)	18-20g	By intraperitoneal injection of STZ (50 mg/kg)	After 5 days of injection and 7 days after the end of injection, the venous blood glucose concentration was detected, which exceeded 16.7 mmol/l	Phenobarbital sodium	PF (100 mg / kg, QD) was orally administered 5 days after STZ injection for 12 weeks	Equal volume of CPBS was injected 5 days after STZ injection for 12 weeks	Pathological changes of renal tissue; 2. BG; 3. 24-h urinary protein; 4. KW/BW ratio; 5. CD68, p-JAK2, and p-STAT3; 6. TNF-α, IL1-β, MCP-1, and iNOS mRNA; 7. p-JAK2/JAK2 and p-STAT3/STAT3
**(Huang2020)**	C57BL/6J mice male (8/8)	18-20g	By intraperitoneal injection of STZ (60 mg/kg)	3 days after intraperitoneal injection of STZ 60mg / kg for 5 consecutive days, the fasting blood glucose FBG of mice was measured to be ≥ 11.1 mmol/l	NM	Phenylsulfonyl PF (CP-25) (70 mg / kg, QD)was orally administered after STZ injection for 5 weeks	Equal volume of CMC administered after STZ injection for 5 weeks	1. Body weight and BG; 2. IPGTT and AUC; 3. FBG, FIN, and HOMA-IR; 4. renal volume and renal index; 5. UAlb / Ucr; 6. SCR, BUN, Chol, and TG; 7. Nephron and GRK2; 8. INR, p-IRS1, and PI3K
**(Li2018)**	C57BL / 6J mice male (12/12)	18-20g	By intraperitoneal injection of STZ (50 mg/kg)	After 5 days of injection and 7 days after the end of injection, the venous blood glucose concentration was detected, which exceeded 16.7 mmol/l	NM	PF (100 mg / kg, QD) was orally administered 5 days after STZ injection for 12 weeks	Equal volume of CPBS was injected 5 days after STZ injection for 12 weeks	Pathological changes of renal tissue; 2. BG; 3. KW/BW ratio; 4. 24-h urinary protein; 5. CD68, p-JAK2, p-STAT3, JAK2, and STAT3; 6. TNF-α, IL1-β, MCP-1, and iNOS mRNA
**(Zhao2022)**	SD rats male (10/10)	200 ± 20 m g	By intraperitoneal injection of STZ (60 mg/kg)	After 72 hours of injection the venous blood glucose concentration was detected, which exceeded 16.7 mmol/l	NM	PF (100 mg / kg, QD) was orally administered after STZ injection for 8 weeks	Captopril (30mg/kg) was administered after STZ injection for 8 weeks	BG; 2. 24-h urinary protein, 3. BUN and Cr; 4. Pathological observation of renal tissue; 5. TNF-α, IL-1β and IL-6; 4. SOD and GSH–Px; 5.SIRT1, Nrf2, and NF-kBp65
**(Duan2018)**	C57BL/6J mice male (12/12)	18-20g	By intraperitoneal injection of STZ (50 mg/kg)	After 5 days of injection and 7 days after the end of injection, the venous blood glucose concentration was detected, which exceeded 16.7 mmol/l	NM	PF (100 mg / kg, QD) was orally administered 5 days after STZ injection for 12 weeks	By oral gavage of an equal volume of distilled water for 12 weeks	1. Pathological changes of renal tissue; 2. BG; 3. 24-h urinary protein; 4. KW/BW ratio; 5. TNF-α, MCP-1, and IL-1β mRNA; 6. TLR2, MyD88, P-IRAK-1, NF-kBp-p65, and NF-kBp65
**(Fu2009)**	SD rats female (10/10)	220-250g	By intraperitoneal injection of STZ (65 mg/kg)	After 3days of injection and 7 days after the end of injection, the venous blood glucose concentration was detected, which exceeded 16 mmol/l	CO2	PF (20 mg / kg, QD) was orally administered after injection for 8weeks	By oral gavage of an equal volume of distilled water for 8 weeks	Pathological changes of renal tissue; 2. Body weight; 3. Kidney weight; 4. BP; 5. BG; 6. Urinary albumin: creatinine ratio; 7. TGF-β, type IV collagen, and ICAM1; 8. MCP1; 9. NFκB and macrophages (ED1-positive cells)
**(Y. Shao2017)**	wild-type (TLR2-/-)and C57BL/6J mice male (12/12)	18-20g	By intraperitoneal injection of STZ (50 mg/kg)	After 5 days of injection and 7 days after the end of injection, the venous blood glucose concentration was detected, which exceeded 16.7 mmol/l	Phenobarbital sodium	PF (100 mg / kg, QD) was orally administered 5 days after STZ injection for 12 weeks	Equal volume of CPBS was injected 5 days after STZ injection for 12 weeks	Pathological changes of renal tissue; 2. BG; 3 24-h urinary protein; 4. TLR2, NF-κBp65, and CD-68; 5.TNF-α, MCP-1, IL-1 β, and iNOS; 6.TLR2, MyD88, p-IRAK1, Trif, p-IRF3, NF-κBp-p65, and NF-κBp65
**(Zhang2017)**	db/db mice male (12/12)	NM	NM	Blood glucose levels were over 16.7 mmol/L	NM	PF (60 mg / kg, QD) was orally administered for 12 weeks	By oral gavage of an equal volume of distilled water for 2 weeks	BG; 2. Body weight; 3. Kidney weight; 4. 24-h urinary protein; 5. Cr; 6. TLR2, TLR4, CD-68, and NF-κBp65; 7. iNOS, TNF-α, IL-1β, and MCP-1; 8. TLR2 and TLR4; 9. MyD88, p-IRAK1, Trif, p-IRF3, NF-κBp-p65, and IL-1β
**(Y. Shao2019)**	C57BL/6J mice and C57BL/10ScN mice males (12/12)	18-20g	By intraperitoneal injection of STZ (50 mg/kg)	NM	Phenobarbital sodium	PF (100 mg / kg, QD) was orally administered 5 days after STZ injection for 12 weeks	By oral gavage of an equal volume of distilled water for 12 weeks	Pathological changes of renal tissue; 2. BG; 3 24-h urinary protein; 4. TNF-α, IL-1β, MCP-1, and iNOS mRNA; 5. MyD88, p-IRAK1, Trif, p-IRF3, NF-κBp-p65, and NF-κBp65

Note: Abbreviations (see Appendix 1 in S1 File);—Data not reported; NM: Not mentioned; p < 0.05 indicates statistical difference.

#### 3.2.1 Study quality

The CAMARADES 10-item quality checklist was used for the assessment of risk of bias in each study. The fulfilled criterion scores ranged from 4 / 10 to 7 / 10, with an average of 5.44. Judgments about the risk of bias items for each included study are detailed in Table 4.

**Table 4:** Risk of bias of the included studies.

(Years)	A	B	C	D	E	F	G	H	I	J	Total
**(Yang2018)**	√	**√**	√	√		√			√		6
**(Li2018)**	√	√	√	√					√		5
**(Huang2020)**	√		√	√			√		√		5
**(Zhao2022)**	√	√	√						√		4
**(Duan2018)**	√	√	√	√					√		5
**(Fu2009)**	√	√	√	√		√			√		6
**(Y. Shao2017)**	√	√	√	√		√			√	√	7
**(Zhang2017)**	√	√	√	√					√		5
**(Y. Shao2019)**	√	√	√	√		√			√		6

Studies fullfiling the criteria of: A peer-reviewed publication; B: control of temperature; C: random allocation to treatment or control; D: blinded induction of model (group randomly after modeling or transgenic mice or knockout mice); E: blinded assessment of outcome; F: use of anesthetic without significant renal protective activity or nephrotoxicity; G: appropriate animal model (aged, hyperlipemia, or hypertensive); H: sample size calculation; I: compliance with animal welfare regulations (including three or more of the following points: preoperative anesthesia, postoperative analgesia, nutrition, disinfection, environment temperature, environment humidity, circadian rhythm, and euthanasia); J: statement of potential conflict of interests.

### 3.3 Effectiveness and mechanism

#### 3.3.1 Pathology analysis and clinical parameters

**Fig 3:**
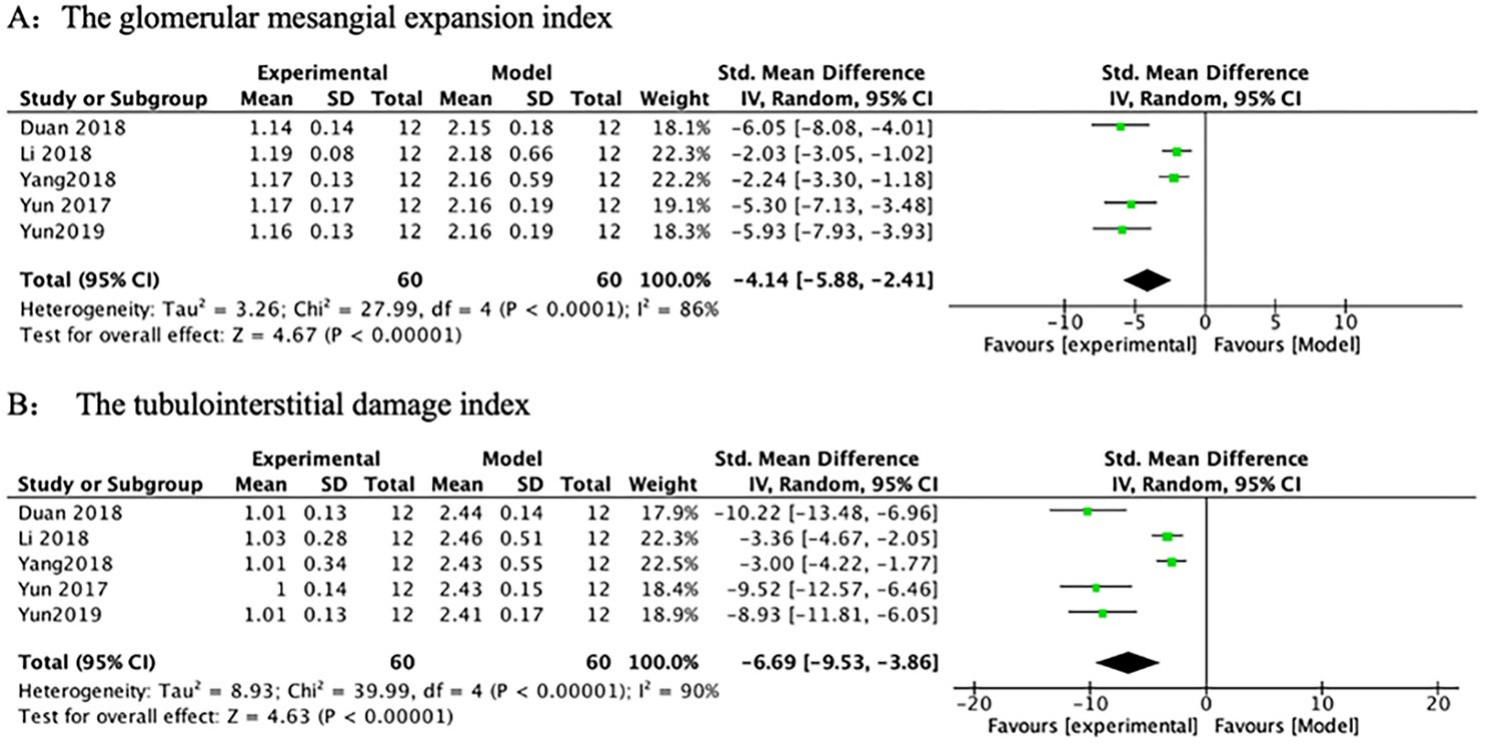
The forest plot: Effects of PF on mesangial expansion index and tubulointerstitial injury index compared with the model group.

*3*.*3*.*1*.*1 Renal pathology*. Among the 9 studies, 5 studies found that PF treatment significantly attenuated the renal histopathological changes compared with the model group, with a significant reduction in mesangial expansion index (Fig 3A) (n = 120, SMD-4.14, 95% CI (-5.88, -2.41), *p* < 0.00001; heterogeneity: χ^2^ = 27.99, *I*^*2*^ = 86%) and tubulointerstitial injury index scores (Fig 3B) (n = 120, SMD -6.69, 95% CI (-9.53, -3.86), *p* < 0.00001; heterogeneity: χ^2^ = 39.99, *I*^*2*^ = 90%). Both mesangial expansion and tubulointerstitial damage indices are strongly associated with renal impairment [27]. Of the 5 studies, 2 studies showed that PF treatment reduced glomerular volume and inhibited mesangial and basement membrane proliferation; 1 study showed that PF treatment reduced extracellular matrix; 1 study reported that PF attenuated renal interstitial congestion, tubular epithelial edema, and inflammatory cell infiltration. The above research findings suggested that PF might significantly prevent DN.

**Fig 4:**
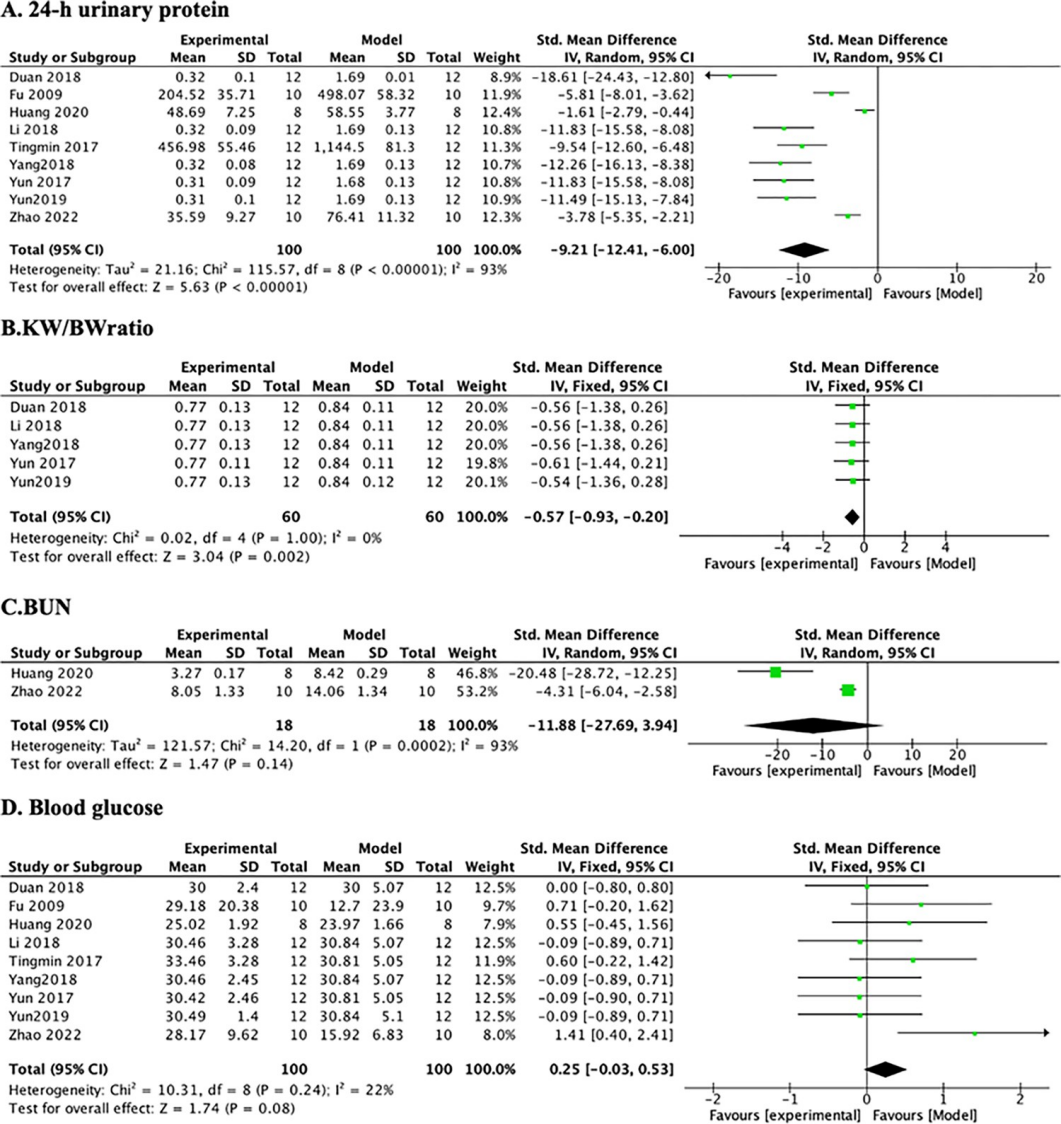
The forest plot: Effects of PF for decreasing 24-h urinary protein, KW/BW ratio, BUN, and BG compared with the model group.

*3*.*3*.*1*.*2 24-h urinary protein*. A meta-analysis of 9 studies showed that mice in the PF intervention group had significantly lower levels of 24-h urinary protein than did mice in the model group (Fig 4A) (n = 200, SMD -9.21, 95% CI (-12.41, -6.00), *p*<0.00001; heterogeneity: χ^2^ = 21.16, *I*^*2*^ = 93%). Subsequently, we performed sensitivity analysis on BG and 24-h urinary indicators. However, there was no significant decrease in heterogeneity after the elimination of any studies.

*3*.*3*.*1*.*3 KW/BW ratio*. A meta-analysis of 5 studies showed that mice in the PF intervention group had significantly lower levels of KW/BW ratio than did mice in the model group (Fig 4B) (n = 120, SMD -0.57, 95% CI (-0.93, -0.20), *p* = 0.002; heterogeneity: χ^2^ = 0.02, *I*^*2*^ = 0%).

*3*.*3*.*1*.*4 BUN*. A meta-analysis of 2 studies showed that mice in the PF intervention group had significantly lower levels of BUN than did mice in the model group (Fig 4C) (n = 36, SMD -11.88, 95% CI (-27.69,3.94), *p* = 0.14; heterogeneity: χ^2^ = 14.20, *I*^*2*^ = 93%).

*3*.*3*.*1*.*5 BG*. A meta-analysis of 9 studies found no significant differences in BG levels between the PF intervention and model groups (*p* > 0.05) (Fig 4D) (n = 200, SMD 0.26, 95% CI (-0.06, 0.59), *p* = 0.11; heterogeneity: χ^2^ = 10.33, *I*^*2*^ = 23%).

#### 3.3.2 Anti-inflammation

**Fig 5:**
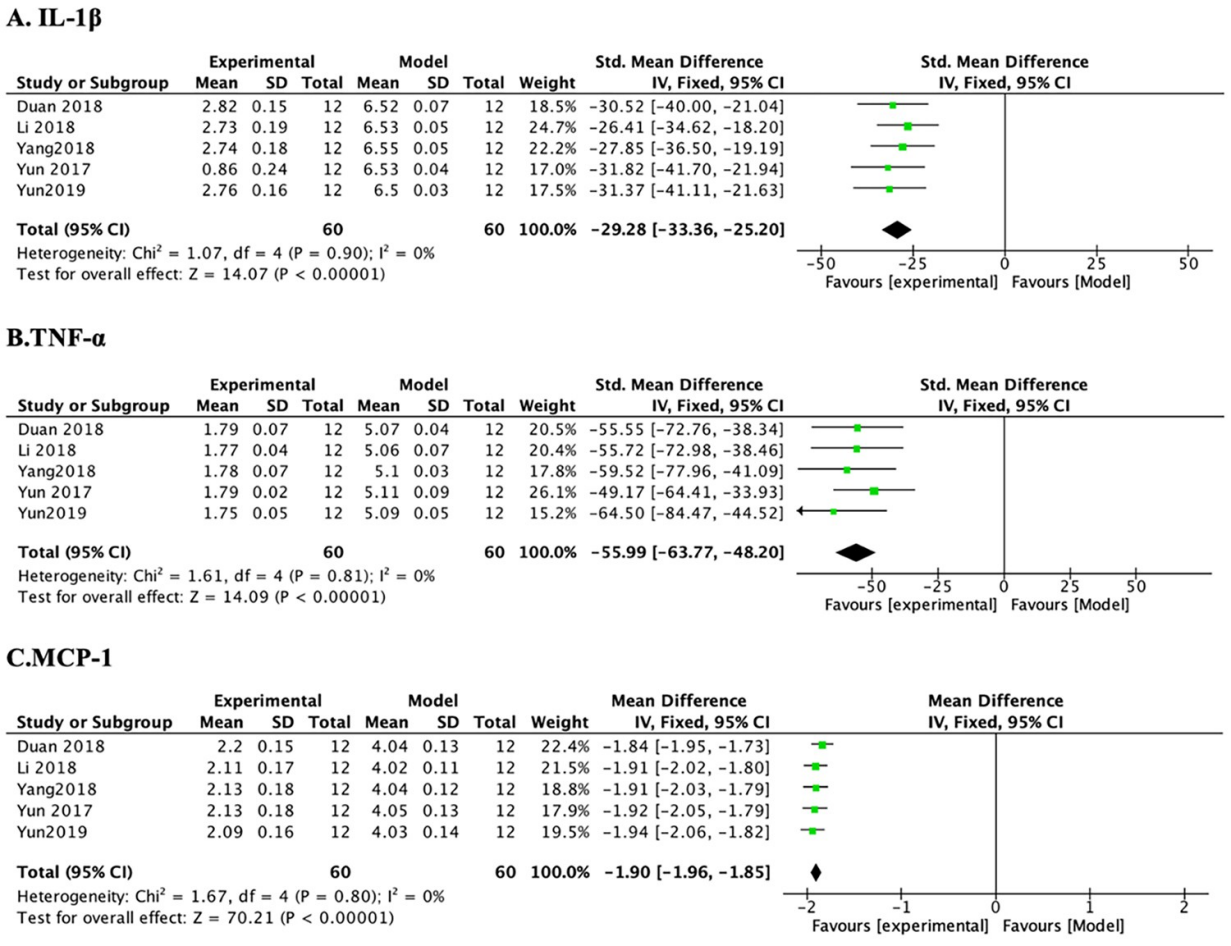
The forest plot: Effects of PF for decreasing mRNA expressions of IL-1β, TNF-α, and MCP-1 compared with the model group.

*3*.*3*.*2*.*1 mRNA expressions of IL-1 β and TNF-α*. Of the 9 studies, 6 studies focused on the changes in IL-1β mRNA expression in the PF treatment group. After sensitivity analysis, 1 study was removed due to that the modeling methods, animal species, high and low PF doses, and length of treatment duration in this study were different from those in other studies. Subsequently, the heterogeneity of IL-1β mRNA expression across studies was decreased substantially (Table 5 and Fig 5A) (n = 120, SMD-29.28, 95% CI (-33.36, -25.20), *p* < 0.00001; heterogeneity: χ^2^ = 1.07, *I*^*2*^ = 0%). Of the 9 studies, 7 studies focused on the changes in TNF-α mRNA expression in the PF treatment group. After sensitivity analysis, 2 studies were removed separately because the modeling methods, animal species, high and low PF doses, and length of treatment duration in these two studies were different from others. Subsequently, the heterogeneity of TNF-α mRNA expression was decreased substantially (Table 5 and Fig 5B) (n = 120, SMD-55.99, 95% CI (-63.77, -48.20), *p* < 0.00001; heterogeneity: χ^2^ = 1.61, *I*^*2*^ = 0%).

**Table 5:** List of the anti-inflammatory effect of PF and its inhibition on macrophage activation.

Variables	Experiments(n)	Individuals (n)	SMD	95% CI	P-value	Heterogeneity
**(1) Effect on inflammatory markers**						
IL-1β	5	120	-29.28	(-33.36, -25.20)	*P* <0.00001	χ^2^ = 1.07, *I*^*2*^ = 0%
TNF-α	5	120	-55.99	(-63.77, -48.20)	*P* <0.00001	χ^2^ = 1.61, *I*^*2*^ = 0%
MCP-1	5	120	-1.90	(-1.96, -1.85)	*P* <0.00001	χ^2^ = 1.67, *I*^*2*^ = 0%
**(2) Effect on macrophage infiltration**						
CD68	6	144	-3.72	(-4.30, -3.14)	*P*<0.00001	χ^2^ = 3.45, *I*^*2*^ = 0%
iNOS	4	96	-33.0	(-38.34, -27.75)	*P<*0.00001	χ^2^ = 9.84, *I*^*2*^ = 69%

*3*.*3*.*2*.*2 mRNA expression of MCP-1*. Of the 9 studies, 7 studies focused on the changes in mRNA expressions of TNF-α and MCP-1 in the PF treatment group. After sensitivity analysis, 2 studies were removed separately because the modeling methods, animal species, doses of PF, and duration of treatment in these 2 studies were different from those in others. Subsequently, the heterogeneity of MCP-1 was decreased substantially (Table 5 and Fig 5C) (n = 120, SMD-1.90, 95% CI (-1.96, -1.85), *p* < 0.00001; heterogeneity: χ^2^ = 1.67, *I*^*2*^ = 0%).

#### 3.3.3 Inhibition of macrophage activation

**Fig 6:**
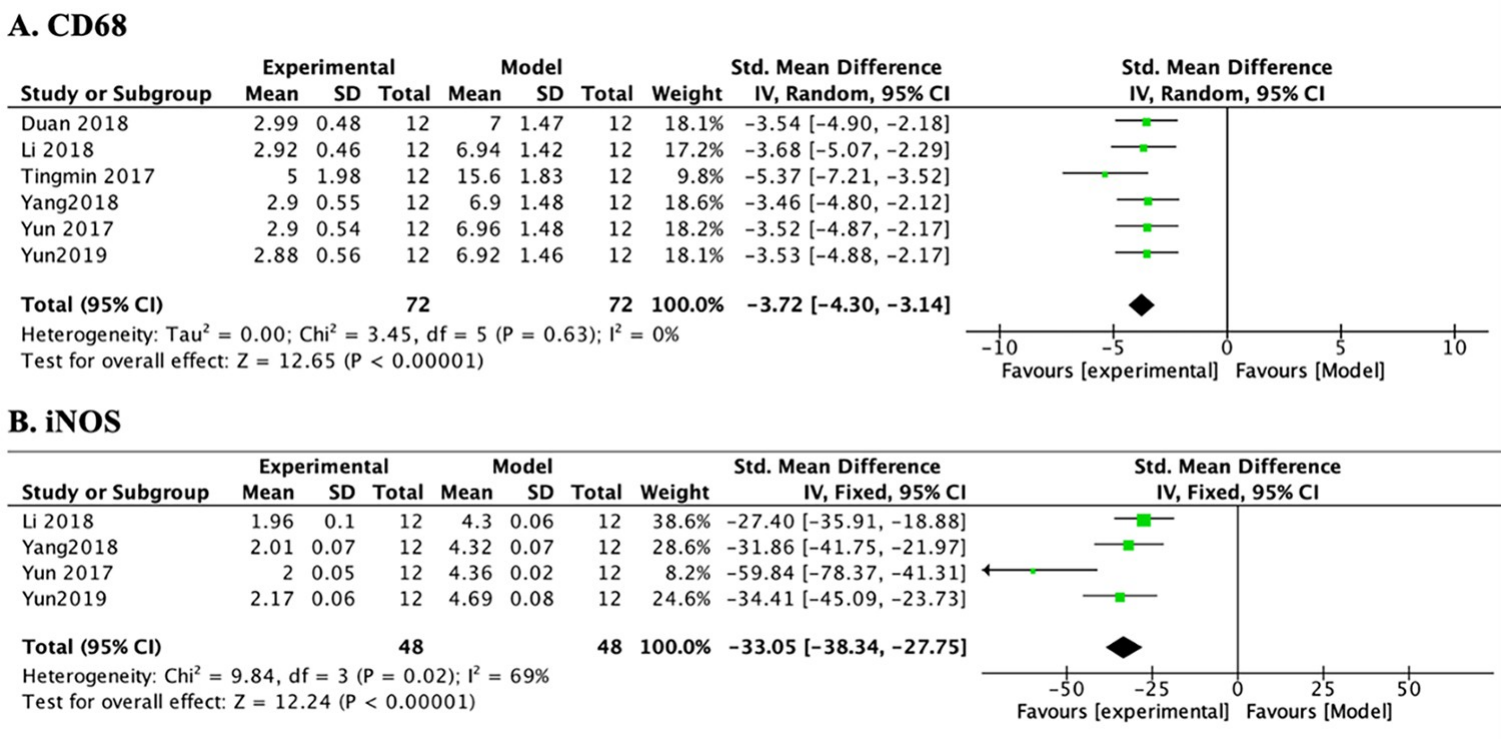
The forest plot: Effects of PF for decreasing mRNA expression of iNOS compared with the model group.

*3*.*3*.*3*.*1 Protein expression of CD68*. Of the 9 studies, 6 studies used CD68-positive macrophages as the observation index. A meta-analysis of 6 studies indicated that PF significantly reduced CD68-positive macrophage infiltration in tubulointerstitial areas (Table 5 and Fig 6A) (n = 144, SMD-3.72, 95% CI (-4.30, -3.14), *p* < 0.00001; heterogeneity: χ^2^ = 3.45, *I*^*2*^ = 0%).

*3*.*3*.*3*.*2 mRNA of iNOS*. Of the 9 studies, 5 studies focused on the effect of PF treatment on iNOS mRNA expression. After sensitivity analysis, 1 study was removed separately because the modeling methods, animal species, doses of PF, and duration of treatment in this study were different from those in other studies. Subsequently, the heterogeneity of iNOS was decreased substantially (Table 5 and Fig 6B) (n = 96, SMD-33.05, 95% CI (-38.34, -27.75), *p* < 0.00001; heterogeneity: χ^2^ = 9.84, *I*^*2*^ = 69%).

#### 3.3.4 Inhibition of signaling pathways and protein expressions

**Fig 7:**
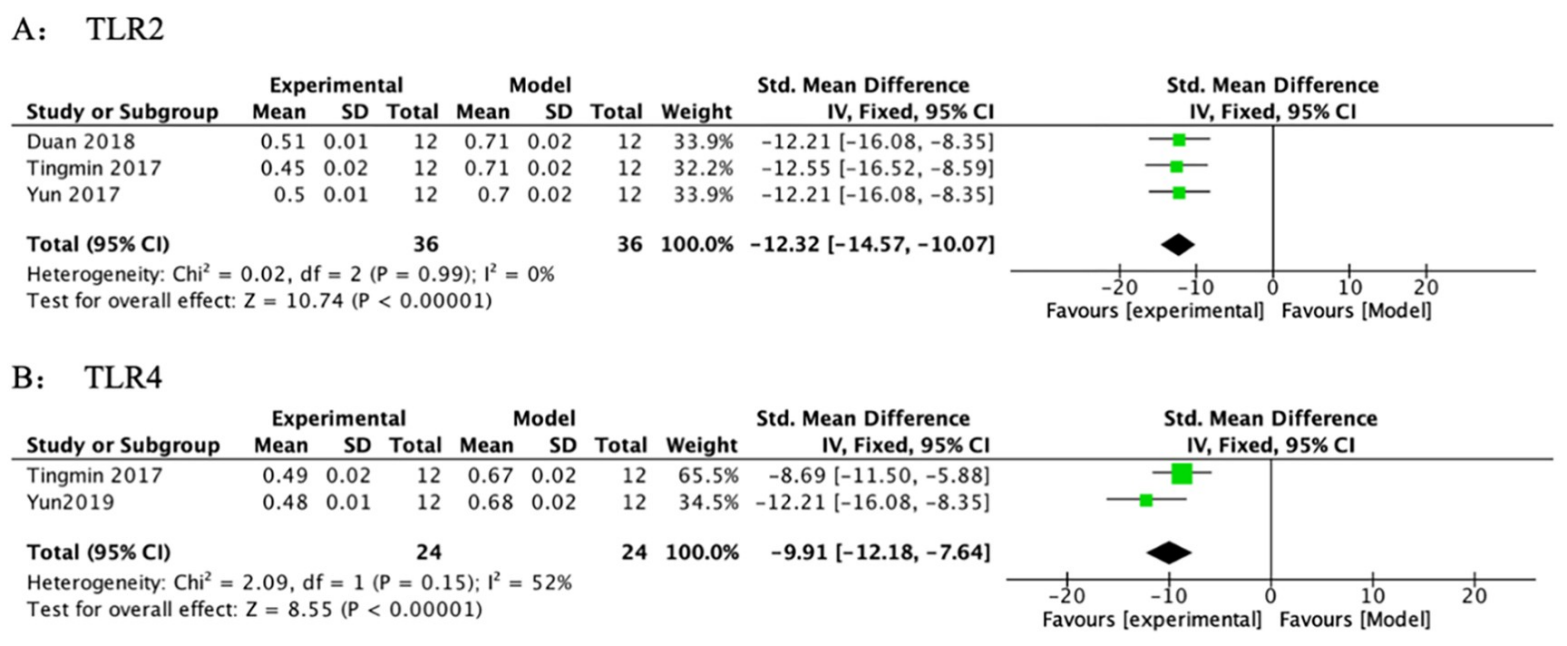
The forest plot: Effects of PF for decreasing TLR2 and TLR4 compared with the model group.

*3*.*3*.*4*.*1 TLR2 and TLR4 signaling pathway*. A meta-analysis across the 3 studies indicated that PF significantly reduced TLR2 levels compared with the model group (Table 6 and Fig 7A) (n = 72, SMD-12.32, 95% CI (-14.57, -10.07), *p* < 0.00001; heterogeneity: χ^2^ = 0.02, *I*^*2*^ = 0%). A meta-analysis across the 2 studies indicated that PF reduced TLR4 levels compared with the model group (Table 6 and Fig 7B) (n = 48, SMD-10.19, 95% CI (-13.61, -6.78), *p* < 0.00001; heterogeneity: χ^2^ = 2.09, *I*^*2*^ = 52%).

**Table 6:** List of the inhibitory effects of PF on signaling pathways and protein expressions.

Variables	Experiments (n)		Individuals(n)	SMD	95% CI	P-value	Heterogeneity
**(1) Effect onTLR2 and TLR4 signaling pathway**							
TLR2		3	72	-12.32	(-14.57, -10.07)	*P* < 0.00001	χ^2^ = 0.02, *I*^*2*^ = 0%
TLR4		2	48	10.19	(-13.61, -6.78)	*P* < 0.00001	χ^2^ = 2.09, *I*^*2*^ = 52%
**(2) Effect on downstream signal factors**							
MyD88		4	96	-15.02	(-21.07, -8.96)	*P* < 0.00001	χ^2^ = 22.28, *I*^*2*^ = 87%
p-IRAK1		3	72	-4.82	(-5.80, -3.83)	*P* < 0.00001	χ^2^ = 1.92, *I*^*2*^ = 0%
Trif		3	72	-8.57	(-10.83, -6.31)	*P* < 0.00001	χ^2^ = 3.87, *I*^*2*^ = 48%
p-IRF3		3	72	-8.60	(-16.07, -1.13)	*P* < 0.00001	χ^2^ = 41.40, *I*^*2*^ = 95%
**(3) Effect on JAK2/ STAT3 signaling pathway**							
p-JAK2 in G		2	48	-6.17	(-6.17, -4.71)	*P* < 0.00001	χ^2^ = 0.28, *I*^*2*^ = 0%
p-JAK2 in T		2	48	-4.35	(-5.46, -3.24)	*P* < 0.00001	χ^2^ = 0.06, *I*^*2*^ = 0%
p-STAT3 in G		2	48	-0.20	(-0.76, 0.37)	*P* = 0.95	χ^2^ = 0, *I*^*2*^ = 0%
p-STAT3 in T		2	48	-8.26	(-10.15, -6.36)	*P* < 0.00001	χ^2^ = 0.16, *I*^*2*^ = 0%
**(4) Effect on NF-κB signaling pathway**							
NF-κB p65		4	92	-7.87	(-9.21, -6.53)	*P* < 0.00001	χ^2^ = 5.46, *I*^*2*^ = 45%
NF-κB p-p65		3	72	-33.16	(-39.11, -27.20)	*P* < 0.00001	χ^2^ = 0.43, *I*^*2*^ = 0%

**Fig 8:**
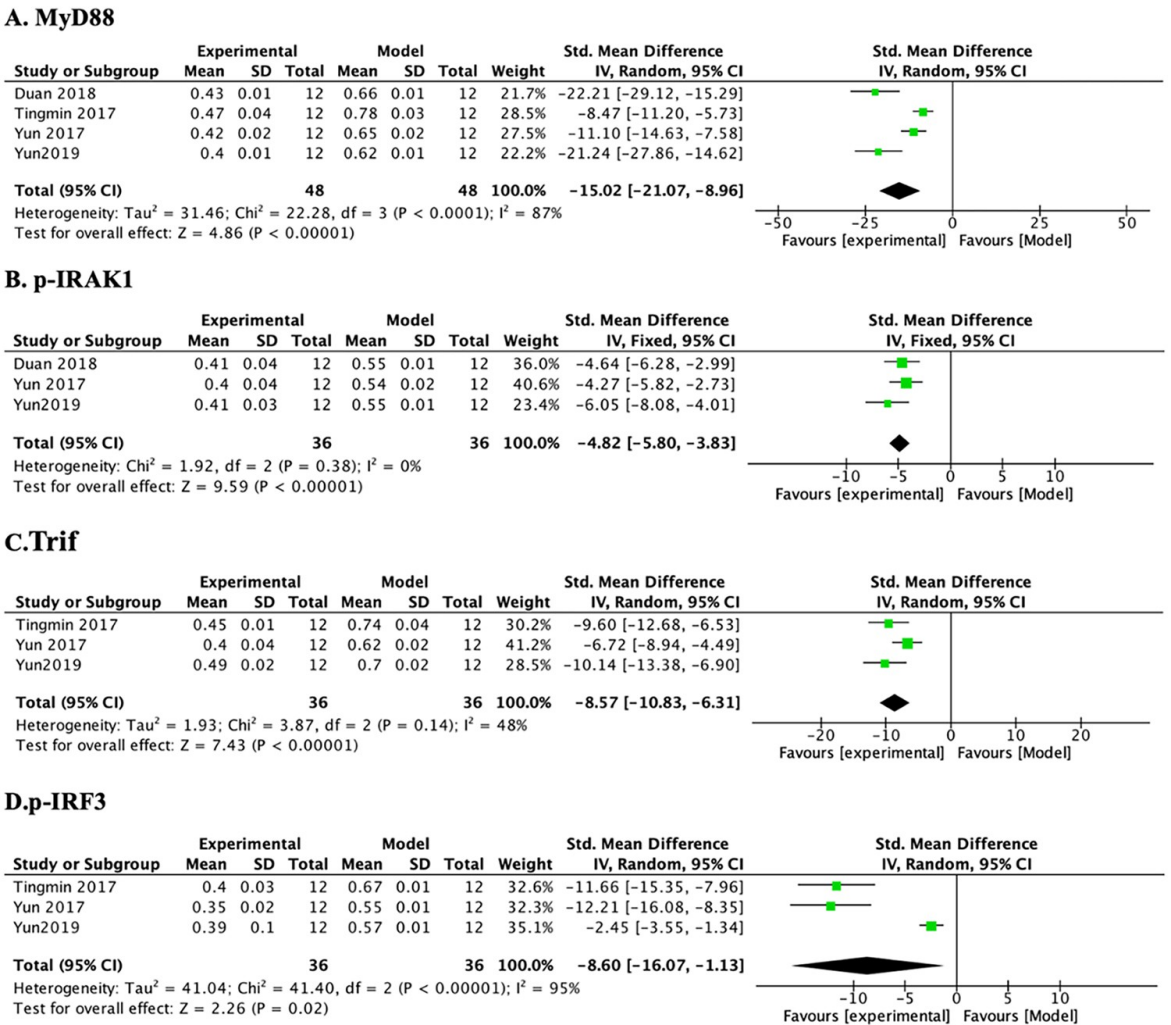
The forest plot: Effects of PF for decreasing MyD88, p-IRAK1, Trif, and p-IRF3 compared with the model group.

*3*.*3*.*4*.*2 Protein expressions of downstream signal factors-MyD88*, *p-IRAK1*, *Trif*, *and p-IRF3*. A meta-analysis across 4 studies indicated that PF reduced MyD88 and p-IRAK1 protein expressions compared to controls. The heterogeneity of MyD88 (Table 6 and Fig 8A) (n = 96, SMD-15.02, 95% CI (-21.07, -8.96), *p* < 0.00001; heterogeneity: χ^2^ = 22.28, *I*^*2*^ = 87%) did not decrease significantly after elimination of any studies. After sensitivity analysis, 1 study was removed separately due to that the modeling methods, animal species, high and low PF doses, and length of treatment duration in this study were different from those in others. Subsequently, the heterogeneity of p-IRAK1 was decreased substantially (Table 6 and Fig 8B) (n = 72, SMD-4.82, 95% CI (-5.80, -3.83), *p* < 0.00001; heterogeneity: χ^2^ = 1.92, *I*^*2*^ = 0%). A meta-analysis across 2 studies showed that the PF group had significantly reduced TRIF (Table 6 and Fig 8C) (n = 72, SMD-8.57, 95% CI (-10.83, -6.31), *p* < 0.00001; heterogeneity: χ^2^ = 3.87, *I*^*2*^ = 48%) and p-IRF3 protein expressions (Table 6 and Fig 8D) (n = 72, SMD-8.60, 95% CI (-16.07, -1.13), *p* = 0.02; heterogeneity: χ^2^ = 41.40, *I*^*2*^ = 95%) compared with the model group (*p* < 0.001).

**Fig 9:**
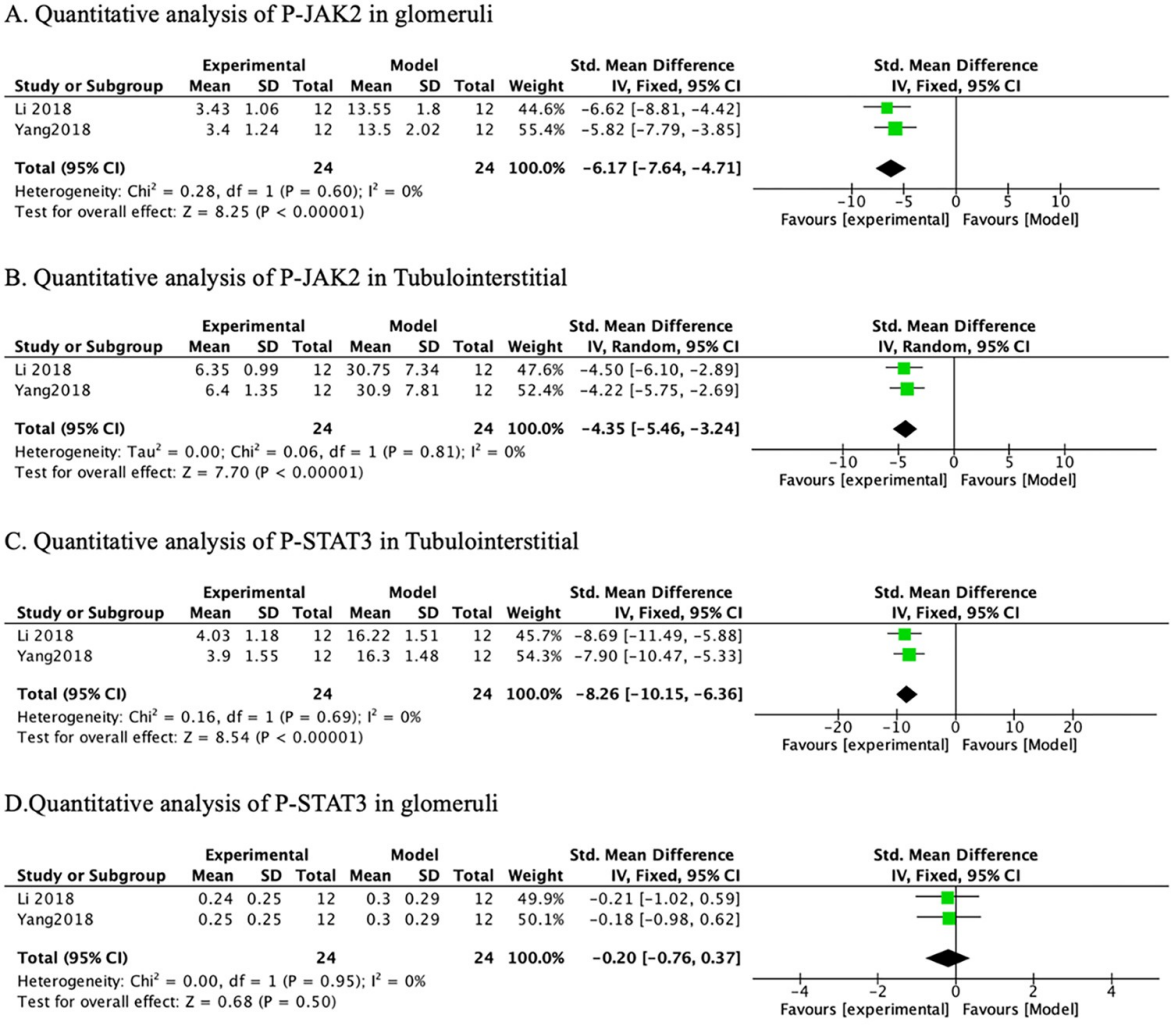
The forest plot: Effects of PF for decreasing p-JAK2 and p-STAT3 compared with the model group.

*3*.*3*.*4*.*3 JAK2/ STAT3 signaling pathway*. A meta-analysis across the 2 studies indicated that PF significantly reduced glomerular (Table 6 and Fig 9A) (n = 48, SMD-6.17, 95% CI (-6.17, -4.71), *p* < 0.00001; heterogeneity: χ2 = 0.28, I^2^ = 0%) and tubulointerstitial (Table 6 and Fig 9B) (n = 48, SMD-4.35, 95% CI (-5.46, -3.24), *p* < 0.00001; heterogeneity: χ2 = 0.06, I^2^ = 0%) P-JAK2 protein levels compared with controls. Also compared with the model group, PF obviously reduced the levels of tubulointerstitial p-STAT3 protein (Table 6 and Fig 9C) (n = 48, SMD-8.26, 95% CI (-10.15, -6.36), *p* < 0.00001; heterogeneity: χ2 = 0.16, I^2^ = 0%) but had no obvious effect on glomerular p-STAT3 protein (Table 6 and Fig 9D) (n = 48, SMD-0.20, 95% CI (-0.76, 0.37), p = 0.95; heterogeneity: χ2 = 0, I^2^ = 0%).

**Fig 10:**
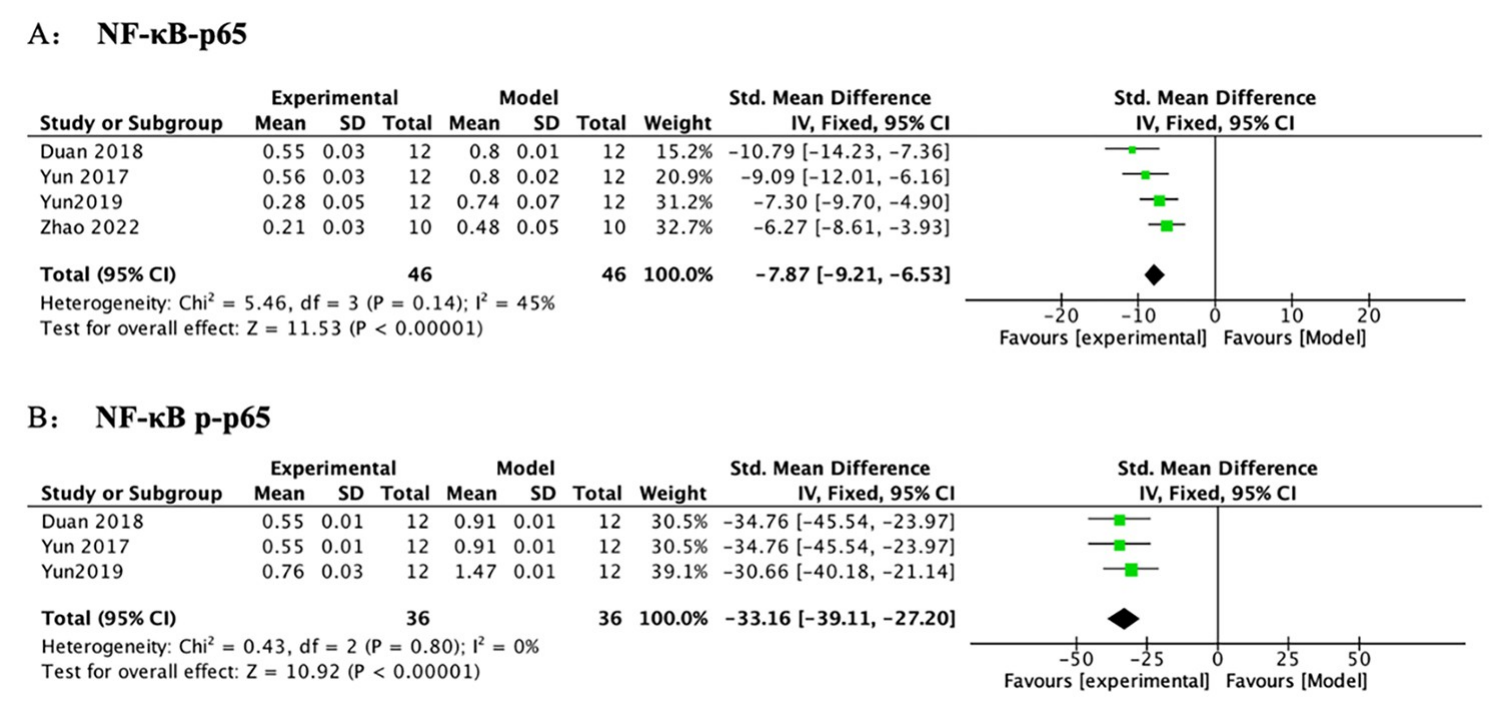
The forest plot: Effects of PF for decreasing NF-κB-p65 and NF-κB p-p65 compared with the model group.

*3*.*3*.*4*.*4 Protein expression of NF-κB p-p65 and NF-κB p65*. A meta-analysis across 5 studies indicated that PF reduced NF-κB-p65 protein compared to controls. After sensitivity analysis, 1 study was removed separately because the modeling methods, doses of PF, animal species, and duration of treatment in this study were varied from those in other studies. Subsequently, the heterogeneity of NF-κB-p65 was decreased substantially (Table 6 and Fig 10A) (n = 92, SMD-7.87, 95% CI (-9.21, -6.53), *p* < 0.00001; heterogeneity: χ^2^ = 5.46, I^2^ = 45%). A meta-analysis across 4 studies revealed that PF reduced NF-κB-p-p65 protein compared to controls. After sensitivity analysis, 1 study was removed separately due to that the animal species, modeling methods, doses of PF, and duration of treatment in this study were different from those in others. Subsequently, the heterogeneity of NF-κB-p-p65 was decreased substantially (Table 6 and Fig 10B) (n = 72, SMD-33.16, 95% CI (-39.11, -27.20), *p* < 0.00001; heterogeneity: χ^2^ = 0.43, I^2^ = 0%).

#### 3.3.5 Time-dose interval analysis

**Fig 11:**
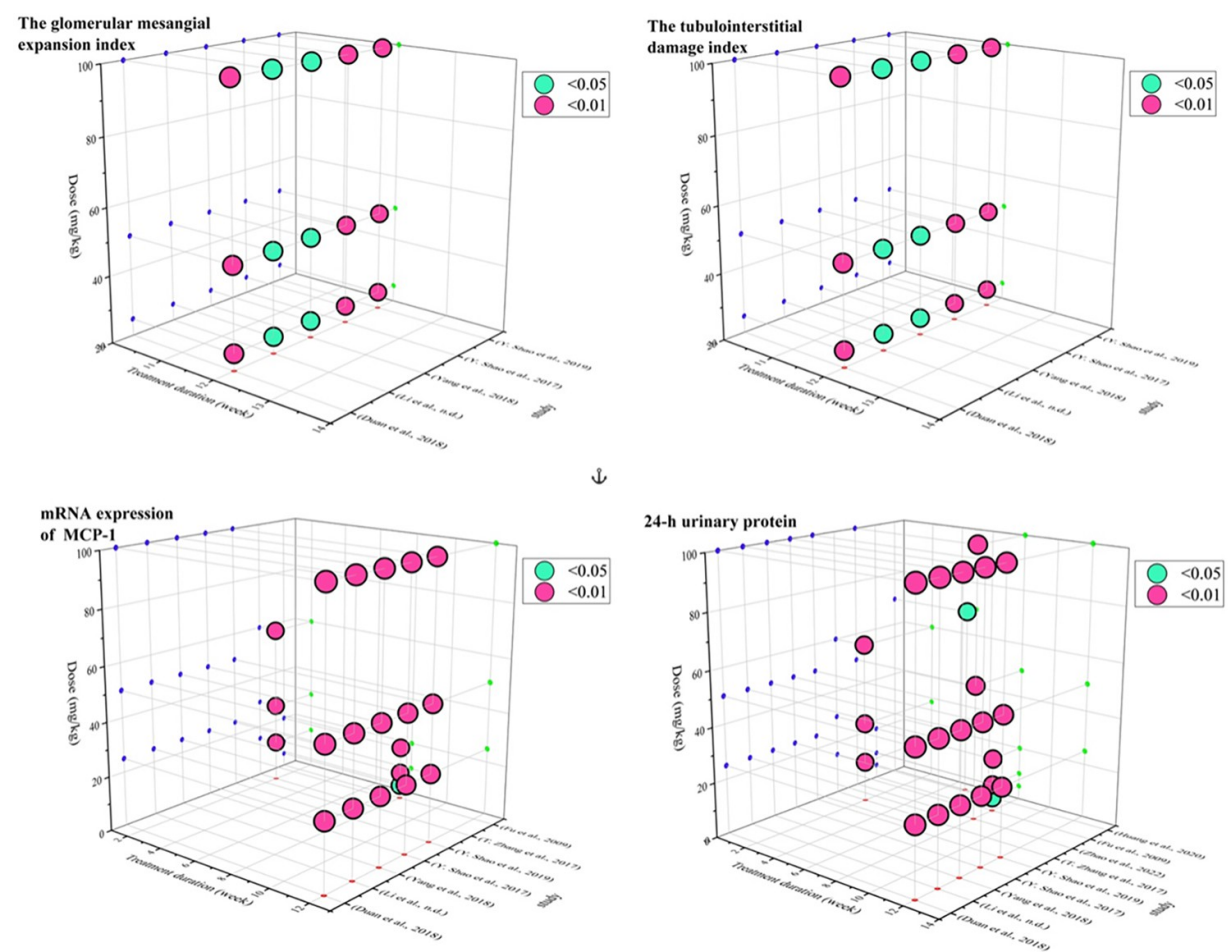
The scatter plot of time-dose interval analysis.

Notably, the time-dose interval analysis can intuitively display the optimal drug dose and treatment duration for providing optimal efficacy. To determine the optimal duration and most reasonable dosage of PF in the treatment of DN, four indicators including mesangial expansion index, tubulointerstitial damage index, 24-h urinary protein, and MCP-1 mRNA were selected for the "time-dose analysis" (Fig 11 and S1-S4 Tables in S1 File). Details on the relationship between the main biochemical markers of renal injury and the dose/time of PF administration is shown in S1-S4 Tables in S1 File. In the analysis of glomerular mesangial dilatation index and renal tubulointerstitial injury index, when the PF dose was maintained at 25~100 mg and the treatment duration was maintained at 12 weeks, the effect of PF treatment group on relieving mesangial expansion and tubulointerstitial injury was better than that of the model group (*p* < 0.05). In the 24-h urinary protein analysis, when the PF dose was maintained at 5~100 mg and the treatment duration was maintained at 2~12 weeks, PF treatment resulted in obvious improving effects on the 24-h urinary protein compared with the model group (*p* < 0.05). In the MCP-1 mRNA expression analysis, when the PF dose was maintained at 5~100 mg and the treatment duration was maintained at 2~12 weeks, PF treatment exerted significant effects on MCP-1 compared to the model group (*p*<0.05).

#### 3.3.6 Subgroup analysis

**Fig 12:**
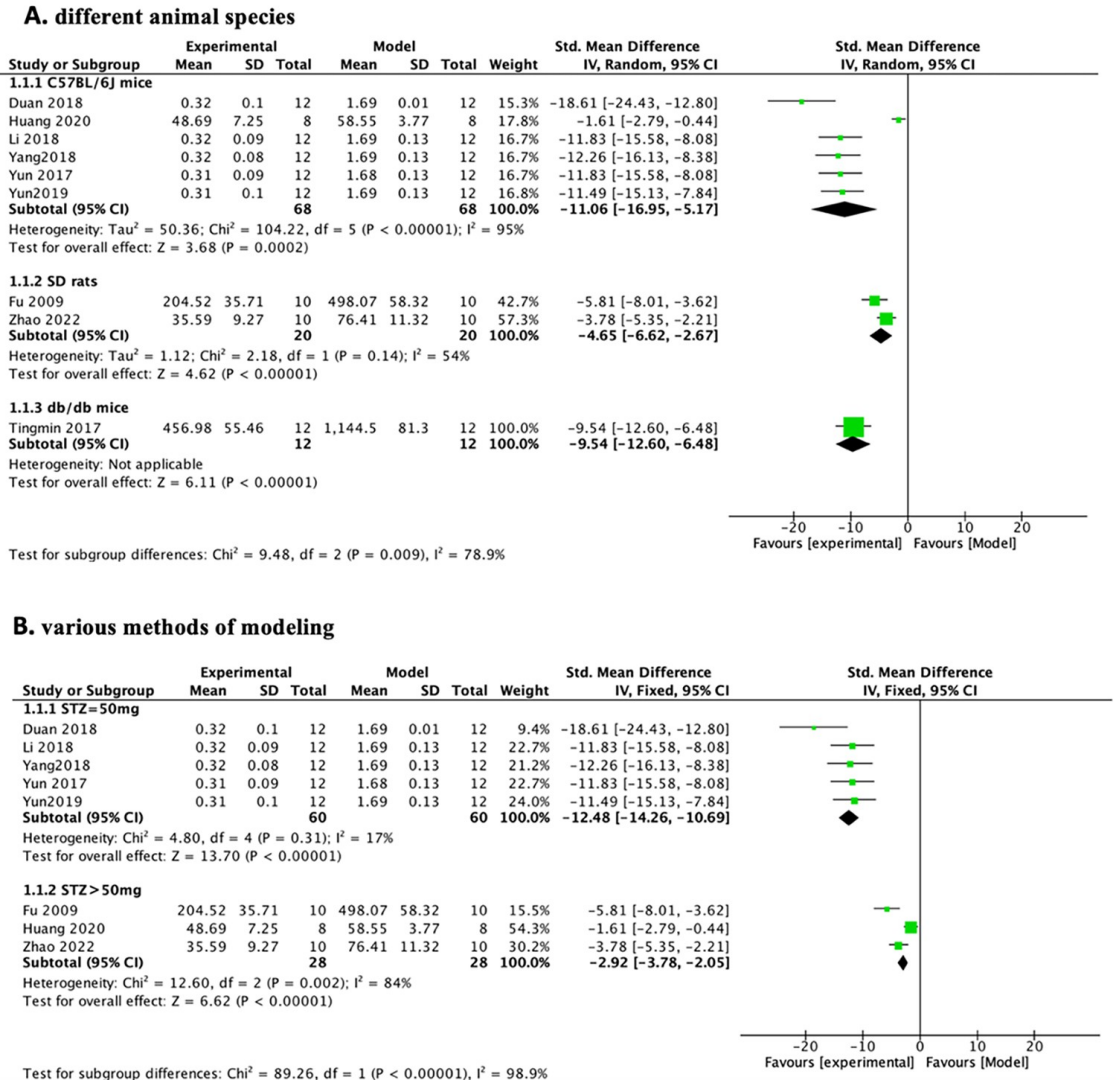
Effects of PF on 24-h urinary protein in subgroups. (A) Induction type; (B) Species.

**Fig 13:**
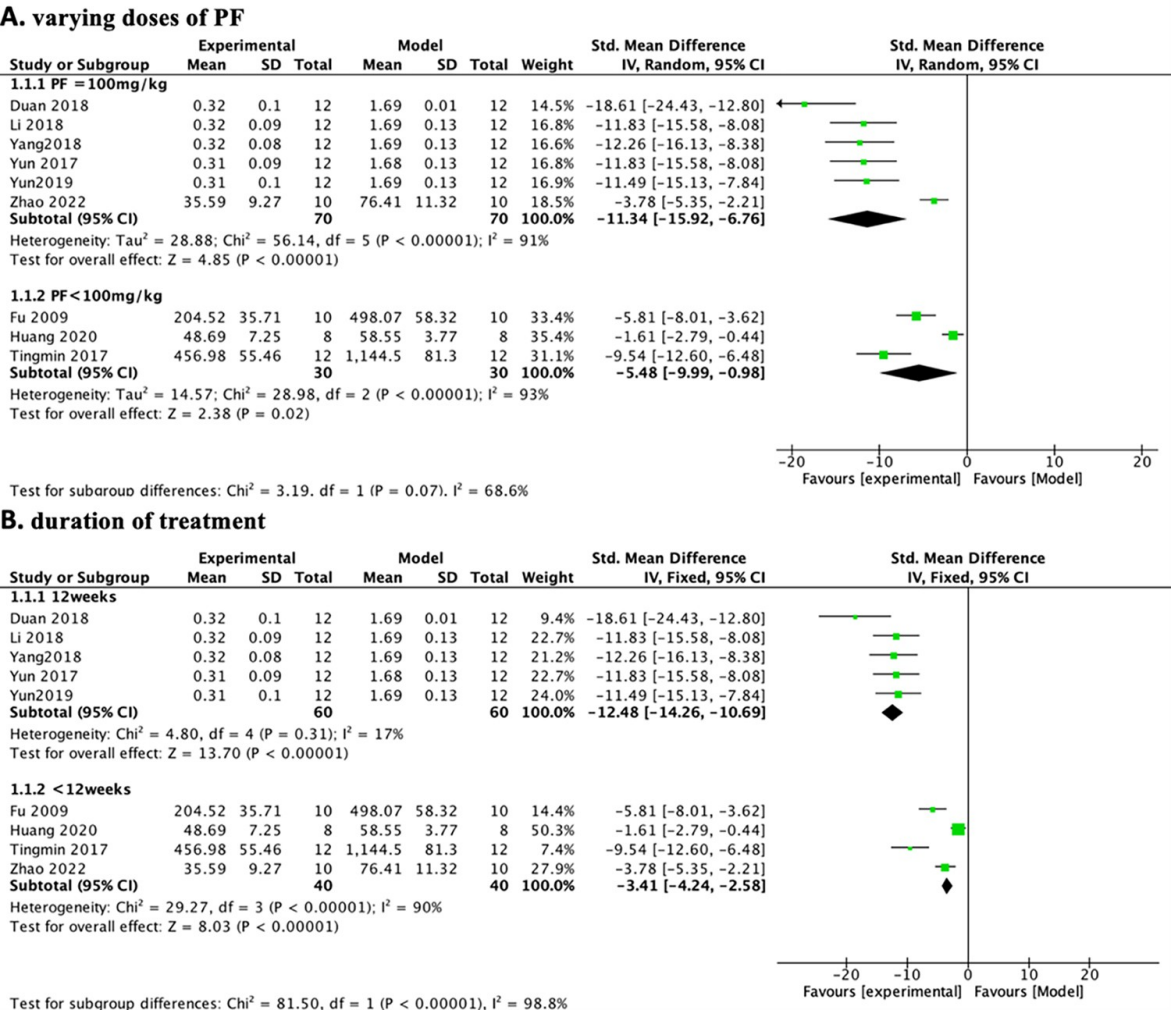
Effects of PF on 24-h urinary protein in subgroups. (A) PF dose; (B) Duration of treatment.

We explored potential confounders (including modeling method, animal species, PF dose, and treatment duration) that may increase 24-hour urinary protein heterogeneity and then performed subgroup analyses of these factors to explore sources of heterogeneity (Table 7). In the subgroup analysis of different animal species, the groups of C57BL/6J mice (Table 7 and Fig 12A)(n = 136, SMD-4.89, 95% CI (-5.87, -3.91, p < 0.00001; heterogeneity: χ2 = 104.22, I^2^ = 95%) were more heterogeneous than the groups of SD rats (Table 7 and Fig 12A) (n = 40, SMD-4.47, 95% CI (-5.75, -3.19, p < 0.00001; heterogeneity: χ2 = 2.18, I^2^ = 54%). To reduce sensitivity, we excluded the literature that did not meet the regulations before subgroup analysis of various modeling methods [26] because it used the db/db diabetic mouse model and did not mention the relevant choice of modeling reagents. The group of DN mice induced by 50 mg/kg STZ (Table 7 and Fig 12B) (n = 120, SMD-12.48, 95% CI (-14.26, -10.69, p < 0.00001; heterogeneity: χ2 = 4.80, I^2^ = 17%) was less heterogeneous than the group of DN mice induced by > 50 mg/kg STZ (Table 7 and Fig 12B) (n = 56, SMD-2.92, 95% CI (-3.78, -2.05, p < 0.00001; heterogeneity: χ2 = 12.60, I^2^ = 84%). Before subgroup analysis of PF doses, we deleted the literature (Huang et al., 2020) because it used a derivative of paeoniflorin (CP-25) to reduce sensitivity. We found that the heterogeneity was not obviously different between the 100 mg/kg PF group (Table 7 and Fig 13A) (n = 140, SMD-11.34, 95% CI (-15.92, -6.76, *p* < 0.00001; heterogeneity: χ^2^ = 56.14, *I*^*2*^ = 91%) and the < 100 mg/kg PF group (Table 7 and Fig 13A) (n = 60, SMD-5.48, 95% CI (-9.99, -0.98, p = 0.002; heterogeneity: χ2 = 28.98, I^2^ = 93%). In the subgroup analysis of duration of treatment, we could see that the group with 12 weeks of treatment (Table 7 and Fig 13B) (n = 120, SMD-12.48, 95% CI (-14.26, -10.69, p < 0.00001; heterogeneity: χ2 = 4.80, I^2^ = 17%) had a significantly lower heterogeneity than the the group with < 12 weeks of treatment (Table 7 and Fig 13B) (n = 80, SMD-3.41, 95% CI (-4.24, -2.58, *p* < 0.00001; heterogeneity: χ^2^ = 29.27, *I*^*2*^ = 90%). Through the above subgroup analyses, it was suggested that modeling methods, animal species, and duration of treatment were potential confounders that might increase the heterogeneity of outcome measures.

**Table 7:** Stratified analysis of pooled estimates according to24-h urinary protein.

Variables	Experiments(n)	Individuals(n)	SMD	95%CI	P-value	Heterogeneity
**species**						
C57BL/6J mice	6	136	-4.89	(-5.87, -3.91)	p < 0.00001	χ^2^ = 104.22, *I*^*2*^ = 95%
SD rats	2	40	-4.47	(-5.75, -3.19)	p < 0.00001	χ^2^ = 2.18, *I*^*2*^ = 54%
**modeling**						
STZ = 50 mg/kg	5	120	-12.48	(-14.26, -10.69)	p < 0.00001	χ^2^ = 4.80, *I*^*2*^ = 17%
STZ > 50 mg/kg	3	56	-2.92	(-3.78, -2.05)	p < 0.00001	χ^2^ = 12.60, *I*^*2*^ = 84%
**Dosage**						
PF = 100 mg/kg	6	140	-11.34	(-15.92, -6.76)	p < 0.00001	χ^2^ = 56.14, *I*^*2*^ = 91%
PF < 100 mg/kg	3	60	-5.48	(-9.99, -0.98)	p = 0.002	χ^2^ = 28.98, *I*^*2*^ = 93%
**Period**						
12 weeks	5	120	-12.48	(-14.26, -10.69)	p < 0.00001	χ^2^ = 4.80, *I*^*2*^ = 17%
<12 weeks	4	80	-3.41	(-4.24, -2.58)	p < 0.00001	χ^2^ = 29.27, *I*^*2*^ = 90%

**Fig 14:**
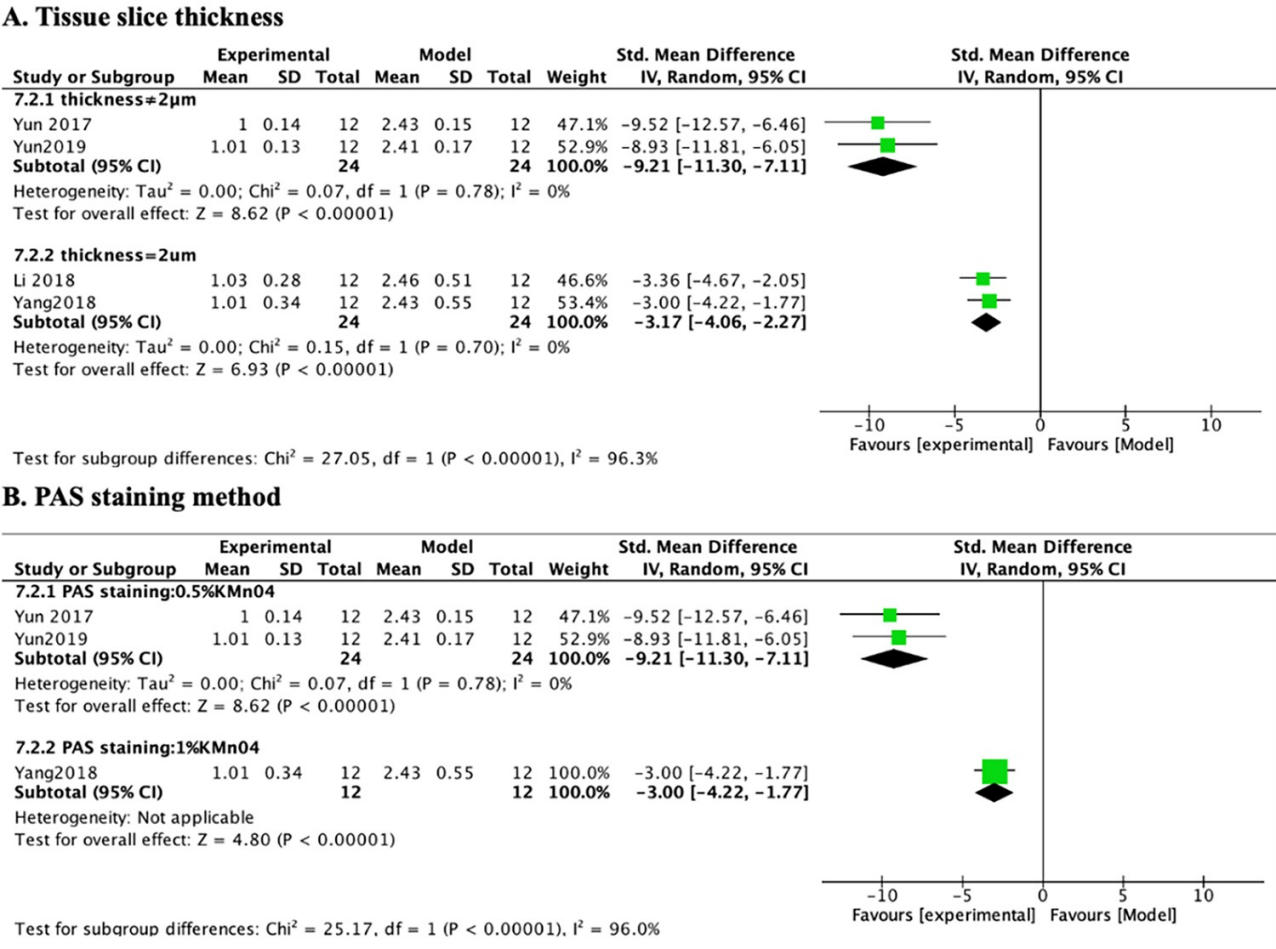
Effects of PF on renal pathology in subgroups. (A) thickness; (B) PAS staining.

Consistently, the same confounding factors were revealed in another previous study with renal pathology as an experimental index [28]. We re-screened the following potential confounding factors (including evaluation and grading of the glomerular mesangial expansion index and the tubulointerstitial damage index, tissue section thickness, and periodic acid-Schiff (PAS) staining method) through subgroup analysis of the renal pathology. In the subgroup analysis of tissue section thickness, 1 of the studies was removed because it did not mention the renal tissue section thickness. The heterogeneity of the group with tissue section thickness 2 μm (Fig 14A) (n = 48, SMD -3.17, 95% CI (-4.06, -2.27, *p* < 0.00001; heterogeneity: χ^2^ = 0.15, *I*^*2*^ = 0%) was substantially reduced compared with other groups (Fig 14A) (n =, 48, SMD -9.21, 95% CI (-11.30, -7.11, *p* < 0.00001; heterogeneity: χ^2^ = 0.07, *I*^*2*^ = 0%). In the subgroup analysis of the PAS staining method, 2 of studies were removed because they did not mention the specific method of PAS staining. Contrasting the group with potassium permanganate concentration 0.5% (Fig 14B) (n = 48, SMD -9.21, 95% CI (-11.30, -7.11, *p* < 0.00001; heterogeneity: χ^2^ = 0.07, *I*^*2*^ = 0%) against the two groups with potassium permanganate concentration 1%, the heterogeneity of the group with potassium permanganate concentration 0.5% was greatly reduced. Through the above subgroup analyses, we considered that the tissue section thickness and the drug concentration of specific experimental procedures (e.g., PAS, HE, and Masson) might also be the potential confounding factors that increased the heterogeneity of outcome measures.

#### 3.3.8 Hypothetical model of the protective mechanisms of PF

**Fig 15:**
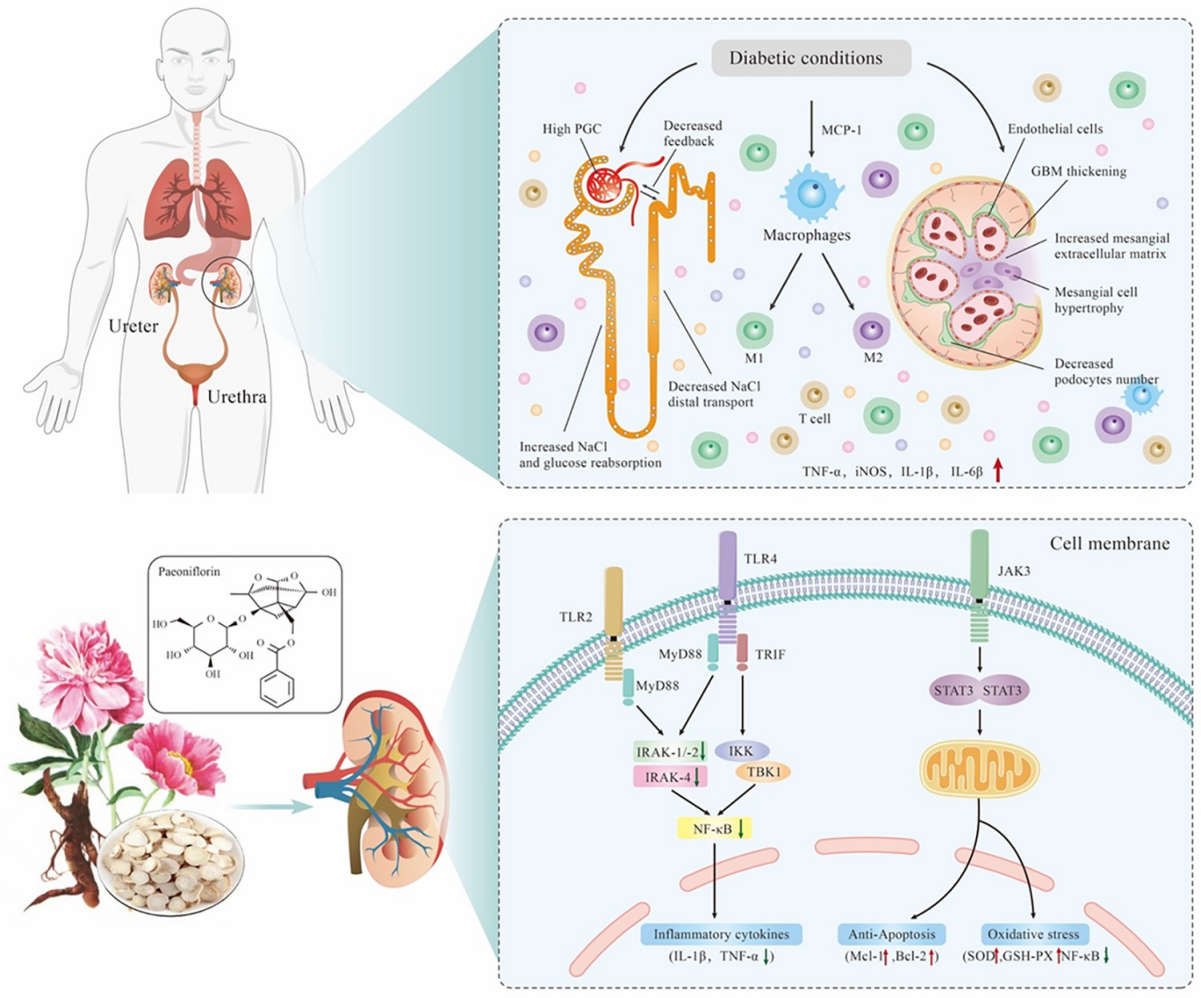
Hypothetical mechanism of the reno-protective effect of PF against DN.

Based on the above literature analysis, we proposed a hypothetical model of the protective mechanisms of PF against DN. PF might exert therapeutic effects on DN through the inhibition of the TLR2/4 signaling pathway under high glucose stimulation to initiate macrophage activation. Its therapeutic effects were not only related to the regulation of inflammatory pathways (e.g., MyD88 and NF-κB pathway) and inflammatory cytokines (e.g., TNF-α, IL-1β, MCP-1), but also related to the regulation of antioxidant effects (e.g., SOD and GSH-PX) (Fig 15). The possible mechanisms of PF in mediating reno-protection are summarized as follows: (1) reducing mesangial expansion index and tubulointerstitial injury index, and alleviating pathological renal injury; (2) inhibiting macrophage infiltration activation and suppressing M1 macrophage production; (3) reducing the production of inflammatory factors (TNF-α, IL-1β, MCP-1) and iNOS, a marker of macrophage activation; (4) decreasing the expression of TLR2/4 and inhibiting their downstream signaling pathways (MyD88, P-IRAK1, Trif, P-IRF3, NF-κB-p-p65, NF-κB-p65; (5) reducing p-JAK2 and p-STAT3 proteins and inhibiting JAK2/STAT3 signaling pathway activation; (6) increasing SOD and GSH-Px activity to reduce the release of MDA, inhibiting renal inflammatory responses, and improving the body peroxidation status, thus alleviating the severity of renal injury.

## 4. Discussion

### 4.1 Summary of evidence

This is the first preclinical systematic review to evaluate the efficacy of PF in animal DN models, including 9 studies with 526 animals. Generally, the included studies are of medium quality. Our analysis results suggest that PF is a multifaceted reno-protective agent for the treatment of DN.

### 4.2 Possible mechanisms

#### 4.2.1 Inhibition of macrophage infiltration and activation

Abnormal macrophage infiltration, recruitment, and activation can be commonly observed in the renal tissues of DN patients and animal DN models, leading to renal immunoinflammatory injury [29]. Activated macrophages can damage renal tissues through the release of inflammatory factors. For example, monocyte macrophages can release proteolytic enzymes and reactive oxygen species clusters that cause glomerular injury, structural remodeling, sclerosis, tubular atrophy, interstitial inflammation, and fibrosis [30].

Ricardo et al. have found that macrophages can be activated into different subtypes under different microenvironments: classically activated (M1-type) and alternatively activated (M2-type), respectively [31]. M1 macrophages promote inflammatory responses and tissue damage [32]. Studies have confirmed that iNOS is one of the marker products of M1 phenotype [33]. The synthesis of iNOS can promote nitric oxide to bind superoxide anion and produce some hydroxyl radical superoxide, thus affecting cell membrane permeability and initiating metabolic abnormalities [34]. Kawagoe et al. have observed an increasing trend in CD68 protein level and MCP-1 mRNA expression in an STZ-induced diabetes model [35]. CD68 is a typical macrophage surface marker protein, which can be used to judge the therapeutic efficacy [36]. MCP-1 can induce fibrocyte proliferation and accelerate glomerulosclerosis to tubulointerstitial transition, gradually leading to the deterioration of renal function [37, 38].

Reducing macrophage infiltration in renal tissues can obviously improve renal function. In STZ-induced rats, CCR2 antagonist can significantly alleviate macrophage infiltration, reduce urinary protein contents, and restore renal function [39]. In our study, PF inhibited macrophage infiltration and reduced the release of active products after macrophage activation, showing obvious anti-inflammatory and immunomodulatory effects.

#### 4.2.2 Regulation of inflammatory changes

Inflammatory response plays an important role in the pathogenesis of DN. Various inflammatory mediators and chemokines influence the progression of DN and contribute to kidney injury [40]. In addition to macrophage infiltration, the role of the TLRs family in the inflammatory mechanism of DN has received wide concerns, especially TLR2 and TLR4 [41, 42] NF-κB can be activated by high glucose, and the presence of NF-κB signaling pathway activation has been observed in peripheral blood mononuclear cells of DN patients [43]. The activation of NF-κB is a marker of renal injury and an important etiological factor of urinary protein in DN patients [44].

Recent studies have found that janus kinase-signal transducer and activator of transcription (JAK-STAT) signaling pathways are also closely related to the development of DN [45]. JAK2/STAT3 gene expression is significantly elevated in DN patients [46]. Activation of the JAK/STAT pathway promotes mesangial cell proliferation and accelerate macrophage infiltration, leading to kidney injury. Experimental results in cultured human mesangial cells have shown that inhibitors of cell signaling pathways can suppress JAK2/STAT3 pathway activation and reduce the corresponding gene expression [47]. Marrero et al., have shown in STZ-induced diabetic rats that AG490 intervention treatment can significantly inhibit glomerular JAK2 and STAT3 phosphorylation, manifesting as a reduction in albuminuria along with a predominance of blood pressure reduction [48]. Our study showed that PF could inhibit the proteins downstream of TLR2/4 and JAK2/STAT3 signaling pathways including MyD88, p-IRAK-1, NF-κB-p-p65, Trif, and p-IRF3, and also reduce the expressions of macrophage inflammatory factors including TNF-α, MCP-1, IL-1β, and iNOS.

#### 4.2.3 Promotion of antioxidant status

Oxidative stress plays a prominent role in the pathogenesis and pathophysiology of DN. A persistent high glucose state may weaken the antioxidant capacity, induce the production of inflammatory factors and reactive oxygen species, and cause damage to the cell structure, further aggravating renal impairment [49]. Oxidative stress is primarily due to the imbalance between oxidants and antioxidants. The degree of imbalance is mainly reflected by GSH-Px, SOD, and MDA [50]. SOD, an endogenous scavenger of oxygen free radicals, contributes to maintaining the antioxidant capacity of the body; GSH-Px can scavenge toxic peroxides in vivo, block free radical damage, and maintain the intracellular redox state [50]. MDA is the product of lipid peroxidation in the body, and the content of MDA can reflect the degree of body oxidative damage [51]. GSH inhibits oxidative stress and abnormal angiogenesis by targeting multiple factors and improving cellular immune responses in DN patients [52].

The study suggested that PF could inhibit renal inflammatory responses and improve the peroxidation status in DN rats via the SIRT1/Nrf2 signaling pathway, thereby alleviating the degree of renal tissue injury and exerting reno-protective effects. SIRT1, a class of histone deacetylases, can regulate oxidative stress, inflammatory response, apoptosis, and many other physiopathological activities [53]. Nrf2 is a core transcription factor that regulates endogenous antioxidant pathways and is bound in the cytoplasm by kelch propylene oxide related protein 1 (Keap1) in the resting state. Activated SIRT1 can change the conformation of Keap1, allowing Nrf2 to dissociate from it, translocate into the nucleus, and become activated. Subsequently, activated Nrf2 can regulate antioxidant pathways and improve the antioxidant capacity of tissues and cells, thereby alleviating the degree of renal oxidative stress [54]. PF potentially regulated the SIRT1/Nrf2/NF- κ B signaling pathways to upregulate GSH-Px and SOD indicators, thereby attenuating renal oxidative stress.

### 4.3 Implications

STZ is a highly selective islet β cytotoxic agent that typically administered as a single high dose, resulting in complete β cell necrosis. Different animals have different sensitivity to STZ. C57BL/6 mice, like Wistar and SD rats, are exquisitely sensitive to STZ [55]. In fact, since the body size of rats facilitates the access to adequate renal tissues for monitoring renal physiological cases, studies are more inclined to select rats to establish diabetes models. STZ dose is one of the most important factors determining whether the DN model is successfully established [56]. STZ at low doses may not successfully induce the expected diabetes model, while high doses (≥ 65 mg/kg) may cause death, nephrotoxicity, or acute tubular necrosis in animals [57, 58]. It is reported that STZ at a dose of 55 mg/kg can significantly avoid the above side responses in rats [59]. Our subgroup analysis of STZ dose showed a significant difference in the 24-h urinary protein between the > 50 mg/kg STZ group and the ≤ 50 mg/kg STZ group, which may be due to that the relatively high doses of STZ (≥ 50 mg/kg) caused nephrotoxicity, rather than hyperglycemia, thus affecting the 24-h urinary protein. Since different types of mice are highly sensitive to STZ, the dosage of STZ should be strictly controlled to avoid high animal mortality and solve the problem of low modeling rate. (1) The protocol employed multiple administrations of low-dose STZ to model pathological outcomes similar to human T1DM with insulitis and insulin deficiency. (2) Animals exposed to a high-fat diet were given an intermediate dose of STZ to reduce the β cell capacity and, as a result, induce hyperglycemia associated with insulin resistance [59, 60]. (3) The suitable temperature and humidity were guaranteed throughout the modeling process. The animals had free access to food and water before STZ injection, and then the mice had to fast for 4 h and 6–8 h, etc.

Although no obvious difference was found in the treatment duration between the high-dose PF group (100 mg/kg) and the low-dose PF group (60 mg/kg), our subgroup analysis of the effect of PF treatment duration on 24-h urinary protein showed a clear difference between the group with 12 weeks of treatment (n = 120, SMD-16.38, 95% CI (-23.78, -8.99, *p* < 0.00001; heterogeneity: χ^2^ = 58.56, *I*^*2*^ = 93%)and the group with < 12 weeks of treatment (n = 44, SMD-0.02, 95% CI (-0.61, 0.57, *p* = 0.94; heterogeneity: χ^2^ = 0, *I*^*2*^ = 0%), suggesting that PF treatment duration was most likely to be the source of heterogeneity. The heterogeneity produced by the length of oral PF exposure may be related to the absorption of PF in the body or the pharmacokinetics of the receptor binding. Numerous pharmacokinetic studies have shown that PF has a low bioavailability low. The bioavailability of PF is around 3% to 4% in rats after oral administration [61, 62], which may be related to the low penetration rate and metabolic pathway of PF. Lipophilicity and p-glycoprotein-mediated efflux can affect the penetration rate of PF in vivo. The metabolic pathways of PF are mainly the hydrolysis of ester bonds, glycosidic bonds, and the conjugation with glucuronic acid [63, 64]. PF is not metabolized by intestinal mucosal enzymes in the intestinal wall, and its degradation rate is also extremely low in the liver and lung [62]. In addition, oral bioavailability is a key factor to ensure that effective drug concentrations are achieved [65]. The low bioavailability of PF limits its clinical application due to its high hydrophilicity and low lipophilicity, low permeability, transporter efflux, and hydrolytic degradation in the intestinal lumen. The present study is still in the phase of exploring whether the drug is effective or not. The results suggest that PF suppresses the immune and restores renal functions in DN. However, the differential relationship between high and low PF doses and efficacy remains uncertain, which may be related to the low bioavailability of PF. Therefore, (1) we suggest to explore approaches to promote PF bioavailability and determine the optimal dosage and therapeutic time frame of PF in DN so as to expand the application of PF in the clinic. Accumulating evidence has confirmed that esterification of hydrophilic compounds can modulate the affinity of PF [66, 67]. (2) Blocking efflux transporters can increase the bioavailability of PF, perhaps by using glucosidase inhibitors. (3) In addition, paeoniflorin-6’-o-benzene sulfonate (cp-25) has been reported to possess a higher bioavailability as a derivative of PF. Cp-25 exhibits a favorable absorption profile, low clearance, long mean residence time, and moderate in vivo bioavailability in rats [68].

### 4.4 Limitations

This meta-analysis has some limitations. (1) the 9 studies included did not report blind induction of models and had flaws in both blind assessment of outcomes and sample size calculation [69]. The arrival guidelines shall be strictly followed for sample size estimation and blind assessment of outcomes in future animal studies. (2) There was a lack of relevant adverse PF outcomes, which may lead to an overestimation of the true effect of PF [70]. (3) The development of DN is often accompanied by comorbidities such as hypertension and hyperglycemia [71]. However, the factors related to hypertension and hyperglycemia were not included in 8 animal models out of 9 studies, which may bias the effectiveness of PF for DN treatment. In future animal studies, the effects caused by multiple factors should be comprehensively considered to minimize the possibility of bias. (4) In the absence of active intervention, DN will progress to ESRD within 6~7 years. The decline rate of renal function varies among DN patients and is influenced by albuminuria, blood pressure, and BG [72, 73]. The models in the 9 studies were all early DN models, 2~3 of which focused on the ability of PF to effectively reduce BUN and Scr and improve the renal function-related clinical indicators. However, it does not mean that the performance of PF treatment is effective throughout DN development. It is necessary to design more rigorous experiments to verify the value of PF in treating different stages of DN.

## 5. Conclusion

The present study demonstrate that PF exerts reno-protective effects in animal models of DN by promoting antioxidant effects, inhibiting macrophage infiltration activation, suppressing inflammatory responses, reducing mesangial cell proliferation, inhibiting endothelial proliferation, attenuating mesangial expansion and tubulointerstitial injury, and inhibiting TLR2 / 4 signaling pathway activation. As there are few negative reports of PF in the included studies, coupled with methodological flaws, the conclusion of benefit should be taken with caution. Follow-up studies according to the arrival guidelines are recommended. Since different types of mice are highly sensitive to STZ, it is recommended to strictly control the dosage of STZ to avoid high animal mortality and solve the problem of low modeling rate. Because of the low bioavailability of PF, further studies on renal histology in animals are urgently needed. It is recommended to actively explore the optimal dosage and therapeutic time frame of PF in the clinic and animals. Moreover, it is necessary to explore methods to improve the PF bioavailability to expand the application of PF in the clinic. In conclusion, our findings suggest that PF is a reno-protective candidate for the treatment of DN.

## Supporting information

S1 Checklist (DOCX)

S1 File (DOCX)

## Abbreviations

AUC: area under the curve
Bax and Bcl-2: BCL2-associated X and B-cell lymphoma-2
BG: blood glucose
CVD: cardiovascular disease
CI: confidence interval
CP-25: paeoniflorin-6’-o-benzene sulfonate
Chol: cholesterol
CD68: cluster of differentiation 68
DN: diabetic nephropathy
DKD: diabetic kidney disease
DM1: type 1 diabetes mellitus
DM2: type 2 diabetes mellitus
ESRD: end-stage renal disease
FBG: fasting blood-glucose
FIN: fasting insulin level
GSH–Px: glutathione peroxidase
GRK2: G protein coupled receptor kinases 2
GFR: glomerular filtration rate
HOMA-IR: homeostasis model assessment
iNOS: inducible nitric oxide synthase
ICAM1: intercellular cell adhesion molecule 1
INR: international normalized ratio
MCP-1: monocyte chemoattractant protein 1
KW / BW ratio: kidney weight / body weight
IL-1β: interleukin 1β
MyD88: myeloid differentiation factor 88
MD: mean difference
NF-κB-p65: nuclear factor kappa p65
NF-κBp-p65: nuclear factor kappa p-p65
Nrf2: nuclear factor erythroid-2 related factor 2
P-irs1: phosphorylated-insulin receptor substrate 1
PF: Paeoniflorin
P-JAK2: phosphorylated-Janus kinase 2
P-IRAK1: phosho-interleukin-1 receptor-associated kinase
P-IRF3: phosphorylated interferon regulatory factor 3
P-STAT3: phosphorylated signal transducer and activator of transcription 3
SOD: superoxide dismutase
SMD: standardized mean difference
SIRT1: silent mating type information regulation 2 homolog 1
SD: Sprague Dawley
SCR: serum creatinine
STZ: streptozotocin
TGP: total glucoside of peony
TLR2: toll like receptors 2
TGFβ1: transforming growth factor-β1
TG: triglyceride
TLR4: toll like receptors 4
TNF-α: tumor necrosis factor-α
TRIF: TIR domain-containing adaptor inducing interferon-β
UAlb/UCR: urinary albumin/urinary creatinine
24-h urinary protein: 24-hour urinary protein excretion rate
