# Supplementary material for: Protective effect of paeoniflorin in diabetic nephropathy: A preclinical systematic review revealing the mechanism of action
Source: PLoS One. 2023 Sep 21;18(9):e0282275. doi: 10.1371/journal.pone.0282275 (PMC10513216; doi:10.1371/journal.pone.0282275)
Supplement: S1 Checklist — (DOCX) [file pone.0282275.s001.docx]

| **Section and Topic** | **Item #** | **Checklist item** | **Location where item is reported** |
| --- | --- | --- | --- |
| **TITLE** | | |  |
| Title | 1 | Protective Effect of Paeoniflorin in Diabetic Nephropathy: A Preclinical Systematic Review Revealing the Mechanism of Action |  |
| **ABSTRACT** | | |  |
| Abstract | 2 | **Background:** Paeoniflorin (PF), the main active glucoside of Paeonia lactiflora, has many pharmacological activities, such as inhibition of vasodilation, hypoglycemia, and immunomodulation. Although current evidence suggest that PF might exert therapeutic effects on diabetic nephropathy (DN), its potential action and mechanism are still unclear.  **Purpose:** A systematic review and meta-analysis of the existing literature on paeoniflorin treatment in DN animal models was performed to evaluate the efficacy and mechanisms of PF evaluated in DN animal models.  **Methods:** The risk of bias in each study will be judged using the CAMARADES 10-item quality checklist with the number of criteria met varying from 4 / 10 to 7 / 10, with an average of 5.44. From inception to July 2022, We searched eight databases. We used the Cochrane Collaboration's 10-item checklist and RevMan 5.3 software to assess the risk of bias and to analyze the data. Three-dimensional dose/time-effect analyses were conducted to examine the dosage/time-response relations between PF and DN.  **Results:** Nine animal studies were systematically reviewed to evaluate the effectiveness of PF in improving animal models of DN.Meta-analysis data and intergroup comparisons indicated that PF slowed the indexof mesangial expansion and tubulointerstitial injury, 24-h urinary protein excretion rate, expression of anti-inflammatory mediators (mRNA of MCP-1, TNF- α,iNOS and IL-1 β), expression of immune downstream factors (P-IRAK1, TIRF, P-IRF3, MyD88, NF- κBp-p65). Furthermore, the modeling methods, animal species, treatment duration, thickness of tissue sections during the experiment, and steps of experimental procedures were subjected to subgroup analysis.  **Conclusion:** The results of the present study demonstrate that the reno-protective effects of PF were associated with its inhibition on macrophage infiltration, reduction of inflammatory mediators, and with its immunomodulatory effects. In conclusion, our findings suggest that PF could effectively slow down the progression of DN and hold promise as a protective drug for the treatment of DN. Because of low PF bioavailability, further studies on renal histology in animals are urgently needed. It is recommended to actively explore the dosage and therapeutic time frame of PF to be used in the clinic and in animals, and methods to improve pf bioavailability, to expand the application of PF in the clinic. |  |
| **INTRODUCTION** | | |  |
| Rationale | 3 | Paeoniflorin (PF), the main active glucoside of Paeonia lactiflora, has many pharmacological activities, such as inhibition of vasodilation, hypoglycemia, and immunomodulation. Although current evidence suggest that PF might exert therapeutic effects on diabetic nephropathy (DN), its potential action and mechanism are still unclear.The risk of bias in each study will be judged using the CAMARADES 10-item quality checklist with the number of criteria met varying from 4 / 10 to 7 / 10, with an average of 5.44. From inception to July 2022, We searched eight databases. We used the Cochrane Collaboration's 10-item checklist and RevMan 5.3 software to assess the risk of bias and to analyze the data. Three-dimensional dose/time-effect analyses were conducted to examine the dosage/time-response relations between PF and DN. |  |
| Objectives | 4 | A systematic review and meta-analysis of the existing literature on paeoniflorin treatment in DN animal models was performed to evaluate the efficacy and mechanisms of PF evaluated in DN animal models. |  |
| **METHODS** | | |  |
| Eligibility criteria | 5 | The criteria for selecting articles were as follows: (1) Population (P): establishment of DN rodent models in a generally accepted manner; (2) Intervention (I) and Control (C): the experimental group received treatment with PF single agent or derivative at any dose, and the model group received treatment with an equal amount of nonfunctional substances (normal saline) or no treatment; (3) Results (R): the primary outcome measures of the study were the changes in histopathological, morphological, and renal function parameters, including 24-h urinary protein excretion rate, etc., while the reno-protective mechanism of PF against DN was selected as the secondary outcome measures, including the changes in metabolic parameters, biochemical parameters, and inflammatory and oxidative stress markers.  The exclusion criteria were as follows: (1) population (P): the target disease was not DN (no DN pattern); (2) Intervention (I): PF based prescription or combination therapy with other drugs; (3) Control (C): unclear comparison with other drugs (e.g., Western pharmaceuticals, combination therapies from traditional Chinese Medicine); (4) Outcome (O): no predefined outcome index or available data; (5) Study design and format: non in vivo studies (in vitro studies, clinical trials, review articles, case reports, meta-analyses, reviews, commentaries, abstracts, editorials, multiple publications or patents). (It is worth noting that although a variety of Paeonia species have been widely used in folk medicine, currently the extracts of these plants are not used in clinical trials alone, but often as part of a prescription. However, the combinational application of multiple TCMs in a formula does not seem to represent the traditional medicinal value of a single plant. Therefore, we excluded clinical trials that included a paeoniflorin compound.) |  |
| Information sources | 6 | Based on database retrieval from Chinese Science and Technology Journal Database, Chinese Biomedical Database, Wan Fang, China National Knowledge Infrastructure, PubMed, the Cochrane library, EMBASE and Web of Science database, we identified animal experimental studies that met the targeted PF treatment for DN. And the search strategies were performed from inception to July 2022. |  |
| Search strategy | 7 | Based on database retrieval from Chinese Science and Technology Journal Database, Chinese Biomedical Database, Wan Fang, China National Knowledge Infrastructure, PubMed, the Cochrane library, EMBASE and Web of Science database, we identified animal experimental studies that met the targeted PF treatment for DN. And the search strategies were performed from inception to July 2022. To obtain a complete literature list, We carefully searched for all eligible studies.Searching in PubMed with mesh search terms, e.g “Diabetic Nephropathy’’, “Diabetic Kidney Diseases”, “Kidney Diseases, Diabetic”, “Intracapillary Glomerulosclerosis”, “Glomerulosclerosis, Nodular”, “Paeoniflorin”, “Paeoniflorin-6'-O-benzene sulfonate” and “Peoniflorin sulfonate”. The specific search methods for the database are summarized in Appendix 1. |  |
| Selection process | 8 | The criteria for selecting articles were as follows: (1) Population (P): establishment of DN rodent models in a generally accepted manner; (2) Intervention (I) and Control (C): the experimental group received treatment with PF single agent or derivative at any dose, and the model group received treatment with an equal amount of nonfunctional substances (normal saline) or no treatment; (3) Results (R): the primary outcome measures of the study were the changes in histopathological, morphological, and renal function parameters, including 24-h urinary protein excretion rate, etc., while the reno-protective mechanism of PF against DN was selected as the secondary outcome measures, including the changes in metabolic parameters, biochemical parameters, and inflammatory and oxidative stress markers.  The exclusion criteria were as follows: (1) population (P): the target disease was not DN (no DN pattern); (2) Intervention (I): PF based prescription or combination therapy with other drugs; (3) Control (C): unclear comparison with other drugs (e.g., Western pharmaceuticals, combination therapies from traditional Chinese Medicine); (4) Outcome (O): no predefined outcome index or available data; (5) Study design and format: non in vivo studies (in vitro studies, clinical trials, review articles, case reports, meta-analyses, reviews, commentaries, abstracts, editorials, multiple publications or patents). (It is worth noting that although a variety of Paeonia species have been widely used in folk medicine, currently the extracts of these plants are not used in clinical trials alone, but often as part of a prescription. However, the combinational application of multiple TCMs in a formula does not seem to represent the traditional medicinal value of a single plant. Therefore, we excluded clinical trials that included a paeoniflorin compound.) |  |
| Data collection process | 9 | Two trained and qualified researchers (ZY and JX) shall independently screen the literature and extract data in strict accordance with the inclusion/exclusion criteria and cross-check them. In case of differences, it shall be decided by the third party (JG). |  |
| Data items | 10a | All eligible data extraction articles were independently evaluated from the following aspects: (1) The year of publication of the study and the name of the first author; (2) Details of animals (quantity, species, sex, weight); (3) The method of establishing animal model and the standard of successful modeling (4) The use of anesthetics in the process of the experiment; (5) Treatment group and control group; (6) Primary and secondary outcomes and intergroup differences. If the results were shown by gradient doses of therapeutic drugs or multiple time points, only the final measurement and data for the highest dose of drugs were used. If the documentation data were in the form of graphs, we attempted to obtain the data from the original author. If raw data could not be acquired, it was measured using the relevant charts of the publicly available Webplotdigitizer. Where results are presented only graphically and not as digital text. |  |
|  | 10b | The criteria for selecting articles were as follows: (1) Population (P): establishment of DN rodent models in a generally accepted manner; (2) Intervention (I) and Control (C): the experimental group received treatment with PF single agent or derivative at any dose, and the model group received treatment with an equal amount of nonfunctional substances (normal saline) or no treatment; (3) Results (R): the primary outcome measures of the study were the changes in histopathological, morphological, and renal function parameters, including 24-h urinary protein excretion rate, etc., while the reno-protective mechanism of PF against DN was selected as the secondary outcome measures, including the changes in metabolic parameters, biochemical parameters, and inflammatory and oxidative stress markers.  The exclusion criteria were as follows: (1) population (P): the target disease was not DN (no DN pattern); (2) Intervention (I): PF based prescription or combination therapy with other drugs; (3) Control (C): unclear comparison with other drugs (e.g., Western pharmaceuticals, combination therapies from traditional Chinese Medicine); (4) Outcome (O): no predefined outcome index or available data; (5) Study design and format: non in vivo studies (in vitro studies, clinical trials, review articles, case reports, meta-analyses, reviews, commentaries, abstracts, editorials, multiple publications or patents). (It is worth noting that although a variety of Paeonia species have been widely used in folk medicine, currently the extracts of these plants are not used in clinical trials alone, but often as part of a prescription. However, the combinational application of multiple TCMs in a formula does not seem to represent the traditional medicinal value of a single plant. Therefore, we excluded clinical trials that included a paeoniflorin compound.) |  |
| Study risk of bias assessment | 11 | Two qualified study trainers (BQ and ZY) assessed the risk of bias for each included study using the CAMARADES 10-item quality checklist ^[14]^ , some of which were modified accordingly and cross-checked. Studies that meet the criteria: A: publications should be peer-reviewed; B: Control of laboratory temperature; C: Experiments should be treated according to the principle of random assignment; D: Application of blinded modeling (modelling by randomisation or use of transgenic knockout mice); E: Blinding was applied to assess outcomes; F: The use of anaesthetics should contain no nephroprotective activity or nephrotoxicity; G: Selection of a reasonable animal model (old age, hyperlipidemia, or hypertension); H: Eligible for sample size calculation; 1：Compliance with animal experimental welfare regulations (≥ three of the following: nutrition, disinfection, ambient temperature and humidity, preoperative anesthesia, postoperative analgesia, and finally euthanasia); J: Declaration of potential conflicts of interest. Each study received a maximum quality score of 10 points, and the median of the calculated results was taken. Study data extraction and quality assessment in case of disagreement will be resolved by consensus or third arbitration between the authors (JG and JZ). |  |
| Effect measures | 12 | Considering the model of measurement instrument as well as animal model differences, etc., we quantitatively determined the summary statistics of the results using the standardized mean difference (SMD) and the corresponding 95% confidence interval (95% CI). When *p* < 0.05 indicated that the difference between the experimental and model groups was statistically significant. For heterogeneity between studies, the I2 statistical test was used. If *I^2^* ≤ 50%, it was considered to have no significant heterogeneity, and a fixed effects model was employed to combine effect sizes. If *I^2^* > 50%, the included studies were of different quality, adopted random effects model, or performed sensitivity analysis. |  |
| Synthesis methods | 13a | Tabulating the study intervention characteristics and comparing against the planned groups for each synthesis.Please refer to Table 1 and Table 2 of the article for details. |  |
|  | 13b | We downloaded and used Revman 5.3 software on the official website to perform statistical analysis on the extracted data. Considering the model of measurement instrument as well as animal model differences, etc., we quantitatively determined the summary statistics of the results using the standardized mean difference (SMD) and the corresponding 95% confidence interval (95% CI). When *p* < 0.05 indicated that the difference between the experimental and model groups was statistically significant. For heterogeneity between studies, the I2 statistical test was used. If *I^2^* ≤ 50%, it was considered to have no significant heterogeneity, and a fixed effects model was employed to combine effect sizes. If *I^2^* > 50%, the included studies were of different quality, adopted random effects model, or performed sensitivity analysis. We performed subgroup analyses for modeling method, animal species, PF dose, and treatment duration to explore the sources of heterogeneity. In addition, time dose interval analysis was performed using Origin 2021 software. |  |
|  | 13c | We will make the single research and comprehensive results into a table or a picture directly displayed by Revman 5.3 software to show the results. |  |
|  | 13d | We downloaded and used Revman 5.3 software on the official website to perform statistical analysis on the extracted data. Considering the model of measurement instrument as well as animal model differences, etc., we quantitatively determined the summary statistics of the results using the standardized mean difference (SMD) and the corresponding 95% confidence interval (95% CI). When *p* < 0.05 indicated that the difference between the experimental and model groups was statistically significant. For heterogeneity between studies, the I2 statistical test was used. If *I^2^* ≤ 50%, it was considered to have no significant heterogeneity, and a fixed effects model was employed to combine effect sizes. If *I^2^* > 50%, the included studies were of different quality, adopted random effects model, or performed sensitivity analysis. |  |
|  | 13e | We performed subgroup analyses for modeling method, animal species, PF dose, and treatment duration to explore the sources of heterogeneity. In addition, time dose interval analysis was performed using Origin 2021 software. |  |
|  | 13f | We performed subgroup analyses for modeling method, animal species, PF dose, and treatment duration to explore the sources of heterogeneity. |  |
| Reporting bias assessment | 14 | Two qualified study trainers (BQ and ZY) assessed the risk of bias for each included study using the CAMARADES 10-item quality checklist ^[14]^ , some of which were modified accordingly and cross-checked. |  |
| Certainty assessment | 15 | Studies that meet the criteria: A: publications should be peer-reviewed; B: Control of laboratory temperature; C: Experiments should be treated according to the principle of random assignment; D: Application of blinded modeling (modelling by randomisation or use of transgenic knockout mice); E: Blinding was applied to assess outcomes; F: The use of anaesthetics should contain no nephroprotective activity or nephrotoxicity; G: Selection of a reasonable animal model (old age, hyperlipidemia, or hypertension); H: Eligible for sample size calculation; 1：Compliance with animal experimental welfare regulations (≥ three of the following: nutrition, disinfection, ambient temperature and humidity, preoperative anesthesia, postoperative analgesia, and finally euthanasia); J: Declaration of potential conflicts of interest. Each study received a maximum quality score of 10 points, and the median of the calculated results was taken. Study data extraction and quality assessment in case of disagreement will be resolved by consensus or third arbitration between the authors (JG and JZ). |  |
| **RESULTS** | | |  |
| Study selection | 16a | Identify 132 records from the update database search .Wan Fang:25;Chinese Science and Technology Journal Database:4;Chinese Biomedical Database:10;China National Knowledge Infrastructure:31;PubMed:13;EMBASE:22;Web of Science database:26;Cochrane library:1 |  |
|  | 16b | A total of 123 articles were excluded and 9 articles were included. The reasons are as follows：Duplicates removed;Records removed for other reasons;Case report; Clinical trial; Review article;Abstracts; Comments and editorials;In Vitro studies;Combined with other medicine;No predetermined outcome index;Lack of control group;No diabetic nephropathy model ;Compared with other traditional Chinese medical ;No available |  |
| Study characteristics | 17 | 9 studies with 526 animals were included. The sample size of each study varied from 8 to 12 animals. Male C57BL / 6 mice were used in 6 studies; Sprague Dawley (SD) male rats were used in 2 studies; Male TLR4 - / - mice were used in 1 study and male TLR2 - / - mice were used in 1 study; Male db / db mice were used in 1 study. 7 studies used spontaneous diabetic mutant or transgenic mice (1 study used db / db mice and 6 studies used C57BL / 6 mice, of which two studies used male TLR4 - / - mice versus male TLR2 - / - mice, respectively). SD rats weigh between 200 and 250 g, and mice weigh between 18 and 20 g. 8 studies established DN models by intraperitoneal streptozotocin (STZ) injection; 1 study used feeding of a high-fat diet for several weeks and intraperitoneal injection of STZ. For induction of anesthesia, 5 studies did not report anesthetics, 3 studies reported sodium pentobarbital and 1 study reported CO2. PF details in each study are shown in Table 1. 9 studies implemented a dose gradient of PF from 5 to 100 mg • kg ^− 1^ • d ^− 1^ using oral or intragastric administration.  To evaluate the effect of PF treatment on DN, 7 studies used renal pathology and 24-h urinary protein as the primary outcome measure; Secondary outcomes were as follows: BG in 9 studies; TNF- α in 6 studies; L1- β in 6 studies; MCP-1 in 7 studies; iNOS in 6 studies; NF- κ B- p65 in 5 studies；NF- κ Bp-p65 in 4 studies; CD68 in 6 studies; MyD88 in 4 studies; P-IRAK1 in 4 studies, TRIF in 3 studies; P-IRF3 in 3 studies; TLR2 in 3 studies；TLR4 in 2 studies; P-JAK2 in 2 studies; P-STAT3 in 2 studies.2 studies reported the KW / BW ratio; 1 study reported AUC; 1 study reported the UAlb / UCR; 1 study reported IL-6; 2 studies reported BUN and 1 study reported SCR; CR was reported in 1 study; Chol was reported in 1 study; 1 study reported TG; 1 study reported FIN;GRK2 was reported in 1 study; 1 study reported INR, 1 study reported p-irs1; 1 study reported glut; 2 studies reported p-JAK2 / JAK2 and 2 studies reported p-STAT3 / STAT3;Bax and Bcl-2 were reported in 1 study; 1 study reported SOD; GSH – Px was reported in 1 study and SIRT1 was reported in 1 study; Nrf2 was reported in 1 study; FBG was reported in 1 study; HOMA-IR was reported in 1 study; TGFβ1 was reported in 1 study, Type IV collagen was reported in 1 study, and ICAM1was reported in 1 study. Detailed characteristics of included studies are shown in Table 2. |  |
| Risk of bias in studies | 18 | We will employ the camarades 10 item quality checklist for the assessment of risk of bias in each study. After evaluation by two qualified researchers, the fulfilled criterion scores ranged from 4 / 10 to 7 / 10, with an average of 5.44. Judgments about the risk of bias items for each included study are detailed in Table 3. |  |
| Results of individual studies | 19 | \| **(years)** \| \| **Species (Sex,**  **n = experimental/**  **control group)** \| \| **Weight** \| \| **Model (method)** \| \| **The Criteria**  **for modeling**  **successfully** \| \| **Anesthetic** \| \| **Treatment group**  **(Method to astragal**  **sides)** \| \| **Control group** \| \| **Outcome index (time)** \| \| \| --- \| --- \| --- \| --- \| --- \| --- \| --- \| --- \| --- \| --- \| --- \| --- \| --- \| --- \| --- \| --- \| --- \| --- \| \| **(Yang2018)** \| \| C57BL/6J mice  male  (12/12) \| \| 18-20g \| \| By intraperitoneal injection of  STZ (50 mg/kg) \| \| After 5 days of injection and 7 days after the end of injection, the venous blood glucose concentration was detected, which exceeded 16.7mmol/l \| \| Phenobarbital sodium \| \| PF (100 mg / kg, QD) was orally administered 5 days after STZ injection for 12 weeks \| \| Equal volume of CPBS was injected 5 days after STZ injection for 12 weeks \| \| 1.Pathological changes of renal tissue; 2.BG 3. 24-h urinary protein;4.KW/BW ratio; 5. CD68 、p-JAK2 、 p-STAT3; 6. TNF-α、IL1-β、MCP-1.iNOS mRNA 7. P-JAK2/JAK2 ,p-STAT3/STAT3 \| \| \| **(Huang2020)** \| \| C57BL/6J  mice  male  (8/8) \| \| 18-20g \| \| By intraperitoneal injection of  STZ (60 mg/kg) \| \| 3 days after intraperitoneal injection of STZ 60mg / kg for 5 consecutive days, the fasting blood glucose FBG of mice was measured to be ≥ 11.1mmol/l \| \| NM \| \| Phenylsulfonyl PF（CP-25）(70 mg / kg, QD)was orally administered after STZ injection for 5 weeks \| \| Equal volume of CMC  administered after STZ injection for 5  weeks \| \| 1. body weight and BG；2. IPGTT and AUC；3. FBG，FIN and HOMA-IR；4. renal volume and renal index；5. UAlb/Ucr；6.SCR, BUN, Chol and TG;7. nephron and GRK2；8. f INR, p-IRS1 and PI3K \| \| \| **(Li2018)** \| \| C57BL / 6J  mice  male  (12/12) \| \| 18-20g \| \| By intraperitoneal injection of  STZ (50 mg/kg) \| \| After 5 days of injection and 7 days after the end of injection, the venous blood glucose concentration was detected, which exceeded 16.7mmol/l \| \| NM \| \| PF (100 mg / kg, QD) was orally administered 5 days after STZ injection for 12 weeks \| \| Equal volume of CPBS was injected 5 days after STZ injection for 12 weeks \| \| 1.Pathological changes of renal tissue; 2.BG;3.KW/BW ratio ;4. 24-h urinary protein; 5.CD68，p-JAK2, p-STAT3, JAK2 and STAT3；6.TNF-α、IL1-β、MCP-1 、iNOS mRNA \| \| \| **(Zhao2022)** \| \| SD  rats  male  (10/10) \| \| 200 ± 20  m  g \| \| By intraperitoneal injection of  STZ (60 mg/kg) \| \| After 72 hours of injection the venous blood glucose concentration was detected, which exceeded 16.7mmol/l \| \| NM \| \| PF (100 mg / kg, QD) was orally administered after STZ injection for 8 weeks \| \| Captopril（30mg/kg） was administered after STZ injection for 8  weeks \| \| 1.BG；2. 24-h urinary protein，3.BUN and Cr；4.Pathological observation of renal tissue; 5.  TNF -α，IL - 1 β,IL-6;4.SOD，GSH–Px;5.SIRT1，Nrf2，NF-kBp65 \| \| \| **(Duan2018)** \| \| C57BL／6J mice  male  (12/12) \| \| 18-20g \| \| By intraperitoneal injection of  STZ (50 mg/kg) \| \| After 5 days of injection and 7 days after the end of injection, the venous blood glucose concentration was detected, which exceeded 16.7mmol/l \| \| NM \| \| PF (100 mg / kg, QD) was orally administered 5 days after STZ injection for 12 weeks \| \| By oral gavage of an  equal volume of  distilled water for 12 weeks \| \| 1. Pathological changes of renal tissue; 2.BG;3. 24-h urinary protein;4.KW/BW ratio;5.TNF-， MCP-1，IL-1βmRNA  6.TLR2、MyD88、P-IRAK-1、NF-KBP-p65、NF-KB p65 \| \| \| **(Fu2009)** \| \| SD  rats  female  (10/10) \| \| 220-250g \| \| By intraperitoneal injection of  STZ (65 mg/kg) \| \| After 3days of injection and 7 days after the end of injection, the venous blood glucose concentration was detected, which exceeded 16mmol/l \| \| CO2 \| \| PF (20 mg / kg, QD) was orally administered after injection for 8weeks \| \| By oral gavage of an  equal volume of  distilled water for 8 weeks \| \| 1.Pathological changes of renal tissue; 2. Body weight ；3.Kidney weight4.BP；5.BG；6.Urinary albumin: creatinine ratio;7.TGFβ, type IV collagen, and ICAM1 8. MCP1 9.NFκB，macrophages (ED1-positive cells) \| \| \| **(Yun2017)** \| \| wild-type（TLR2-/-）and C57BL/6J  mice  male  (12/12) \| \| 18-20g \| \| By intraperitoneal injection of  STZ (50 mg/kg) \| \| After 5 days of injection and 7 days after the end of injection, the venous blood glucose concentration was detected, which exceeded 16.7mmol/l \| \| Phenobarbital sodium \| \| PF (100 mg / kg, QD) was orally administered 5 days after STZ injection for 12 weeks \| \| Equal volume of CPBS was injected 5 days after STZ injection for 12 weeks \| \| 1.Pathological changes of renal tissue; 2. BG;3 24-h urinary protein; 4. TLR2, NF-κB p65and  CD-68 ;5.TNF- α， MCP-1，IL-1 β And iNOS;6.TLR2,MyD88,p-IRAK1, Trif, p-IRF3,NF-κBpp65,  NF-κB p65 \| \| \| **(Zhang2017)** \| \| db/db mice  male  (12/12) \| \| NM \| \| NM \| \| blood  glucose levels were over 16.7 mmol/L \| \| NM \| \| PF (60 mg / kg, QD) was orally administered for 2 weeks \| \| By oral gavage of an  equal volume of  distilled water for 2 weeks \| \| 1.BG;2.Body weight;3.Kidney weight;4. 24-h urinary protein；5.Ccr6.TLR2, TLR4, CD-68 and NF-κB p65;7.iNOS, TNF-α, IL-1β and MCP-1  8.TLR2, TLR4  9.MyD88,p-IRAK1, Trif, p-IRF3, NF-κB p-p65 , and  IL-1β \| \| \| **(Yun2019)** \| \| C57BL/6J mice and C57BL/10ScN mice  males  （12/12） \| \| 18-20g \| \| By intraperitoneal injection of  STZ (50 mg/kg) \| \| NM \| \| Phenobarbital sodium \| \| PF (100 mg / kg, QD) was orally administered 5 days after STZ injection for 12 weeks \| \| By oral gavage of an  equal volume of  distilled water for 12 weeks \| \| 1.Pathological changes of renal tissue; 2.BG 3 24-h urinary protein; 4.TNF-a, IL-1B, MCP-1, and iNOS mRNA;5.MyD88, p-IRAK1, Trif, p-IRF3,NF-Bp-p65, and NF-Bp65 \| \| \|  \| \|  \| \|  \| \|  \| \|  \| \|  \| \|  \| \|  \| \|  \| \| |  |
| Results of syntheses | 20a | We will employ the camarades 10 item quality checklist for the assessment of risk of bias in each study. After evaluation by two qualified researchers, the fulfilled criterion scores ranged from 4 / 10 to 7 / 10, with an average of 5.44. Judgments about the risk of bias items for each included study are detailed in Table 3.  Studies fulﬁlling the criteria of: A peer-reviewed publication; B: control of temperature; C: random allocation to treatment or control; D: blinded induction of model (group randomly after modeling or transgenic mice or knockout mice); E: blinded assessment of outcome; F: use of anesthetic without signiﬁcant renal protective activity or nephrotoxicity; G: appropriate animal model (aged, hyperlipemia or hypertensive); H: sample size calculation; I: compliance with animal welfare regulations (including three or more of the following points: preoperative anesthesia, postoperative analgesia, nutrition, disinfection, environment temperature, environment humidity, circadian rhythm, and euthanasia); J: statement of potential conﬂict of interests.   \| **(years)** \| **A** \| **B** \| **C** \| **D** \| **E** \| **F** \| **G** \| **H** \| **I** \| **J** \| **Total** \| \| --- \| --- \| --- \| --- \| --- \| --- \| --- \| --- \| --- \| --- \| --- \| --- \| \| **(Yang2018)** \| √ \| **√** \| √ \| √ \|  \| √ \|  \|  \| √ \|  \| 6 \| \| **(Li2018)** \| √ \| √ \| √ \| √ \|  \|  \|  \|  \| √ \|  \| 5 \| \| **(Huang2020)** \| √ \|  \| √ \| √ \|  \|  \| √ \|  \| √ \|  \| 5 \| \| **(Zhao2022)** \| √ \| √ \| √ \|  \|  \|  \|  \|  \| √ \|  \| 4 \| \| **(Duan2018)** \| √ \| √ \| √ \| √ \|  \|  \|  \|  \| √ \|  \| 5 \| \| **(Fu2009)** \| √ \| √ \| √ \| √ \|  \| √ \|  \|  \| √ \|  \| 6 \| \| **(Yun2017)** \| √ \| √ \| √ \| √ \|  \| √ \|  \|  \| √ \| √ \| 7 \| \| **(Zhang2017)** \| √ \| √ \| √ \| √ \|  \|  \|  \|  \| √ \|  \| 5 \| \| **(Yun2019)** \| √ \| √ \| √ \| √ \|  \| √ \|  \|  \| √ \|  \| 6 \| |  |
|  | 20b | **Effectiveness and mechanism**  **Pathology Analysis and Clinical parameters**  **Renal Pathology**  Compared with the model group, among the 9 studies, 5 studies evaluated the renal histopathological changes, and PF treatment significantly attenuated the renal histopathological changes, with a significant reduction in mesangial expansion index (Fig. 2-A) (n = 120, SMD-4.14, 95% CI (-5.88, -2.41), *p*＜0.00001; heterogeneity: χ^2^ = 27.99, *I^2^* = 86%) and tubulointerstitial injury index scores (Fig. 2-B) (n = 120, SMD -6.69, 95% CI (-9.53, -3.86), *p*＜0.00001; heterogeneity: χ^2^ =39.99, *I^2^* = 90%). Both mesangial expansion and tubulointerstitial damage indices were found to be strongly associated with renal impairment^[25]^. The above research data suggested that PF might significantly prevent DN. studies reduced glomerular volume and 2 studies inhibited mesangial, and basement membrane proliferation; 1 study reduced extracellular matrix; 1 study attenuated renal interstitial congestion, tubular epithelial edema, and inflammatory cell infiltration.  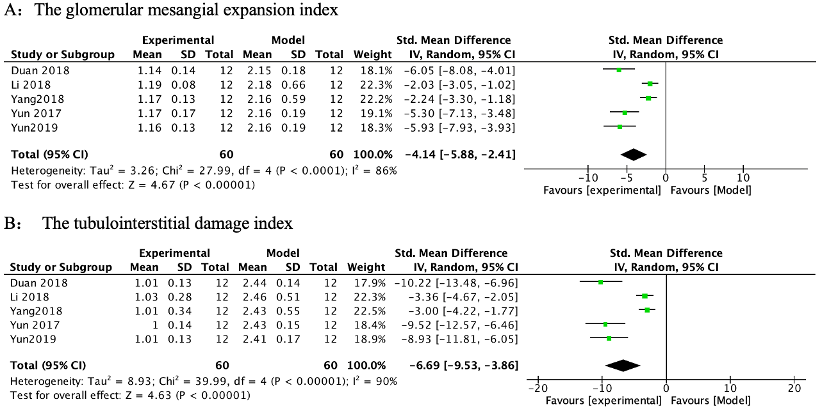  ***Fig. 2. The forest plot: effect of paeoniflorin on mesangial expansion index and tubulointerstitial injury index compared with the model group***  **24-h urinary protein**  A meta-analysis of 9 studies showed that mice in the PF intervention group had significantly lower levels of 24-h urinary protein than did mice in the model group (Fig. 3-A) (n = 200, SMD -9.21, 95% CI (-12.41, -6.00), *p*＜0.00001; heterogeneity: χ^2^ = 21.16, *I^2^* = 93%). Subsequently, we performed sensitivity analysis on BG and 24-h urinary indicators. However, there was no significant decrease in heterogeneity after the elimination of any studies.  **KW/BW ratio**  A meta-analysis of 5 studies showed that mice in the PF intervention group had significantly lower levels of KW/BW ratio than did mice in the model group (Fig. 3-B) (n =120, SMD -0.57, 95% CI (-0.93, -0.20), *p*＝0.002; heterogeneity: χ^2^ =0.02, *I^2^* = 0%).  **BUN**  A meta-analysis of 2 studies showed that mice in the PF intervention group had significantly lower levels of BUN than did mice in the model group (Fig. 3-C) (n = 36, SMD -11.88, 95% CI (-27.69,3.94), *p*＝0.14; heterogeneity: χ^2^ = 14.20, *I^2^* = 93%).  **Blood glucose**  A meta-analysis of 9 studies found no significant differences in BG levels between intervention and model groups in PF mice (*p* > 0.05) (Fig. 3-D) (n = 200, SMD 0.26, 95% CI (-0.06, 0.59), *p* = 0.11; heterogeneity: χ^2^ = 10.33, *I^2^* = 23%).  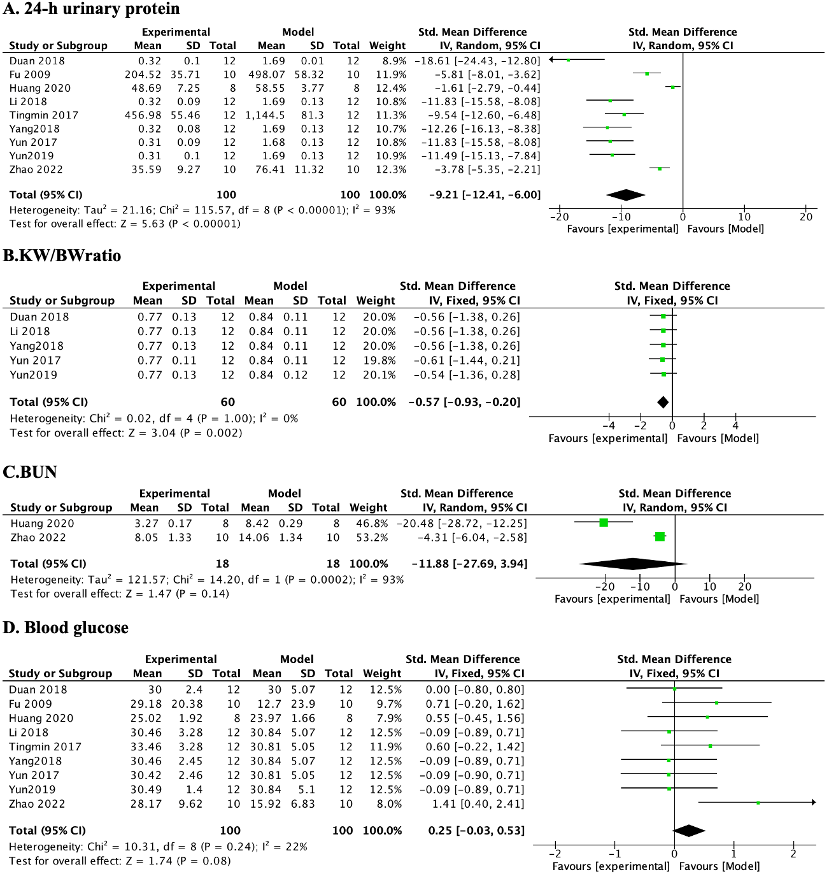  ***Fig. 3. The forest plot: effects of PF for decreasing 24-h urinary protein, KW/BW ratio, BUN and BG compared with the model group.***  **Anti-inflammatory**  **mRNA expression of IL-1 β, TNF- α**  Of the 9 studies, 6 studies focused on the changes in IL-1β mRNA expression in the PF treatment group. After sensitivity analysis, 1 study was removed separately due to that the modeling methods, different animal species, high and low PF doses and length of treatment duration in this study were different from others. Subsequently, the heterogeneity of IL-1β mRNA expression across studies was decreased substantially (Table 4 and Fig. 4-A) (n =120, SMD-29.28, 95% CI (-33.36, -25.20), *p* < 0.00001; heterogeneity: χ^2^ =1.07, *I^2^* = 0%). Of the 9 studies, 7 studies focused on the changes in TNF-α mRNA expression in the PF treatment group. After sensitivity analysis, 2 studies were removed separately because the modeling methods, different animal species, high and low PF doses and length of treatment duration in these two studies were different from others. Subsequently, the heterogeneity of TNF-α mRNA expression was decreased substantially (Table 4 and Fig. 4-B) (n =120, SMD-55.99, 95% CI (-63.77, -48.20), *p* < 0.00001; heterogeneity: χ^2^ =1.61, *I^2^* = 0%).  **mRNA expression of MCP-1**  Of the 9 studies, 7 studies focused on the changes in mRNA expressions of TNF-α and MCP-1 in the PF treatment group. After sensitivity analysis, 2 studies were removed separately because the modeling methods, animal species, doses of PF, and duration of treatment in these 2 studies were different from others. Subsequently, the heterogeneity of MCP-1 was decreased substantially (Table 4 and Fig. 4-C) (n = 120, SMD-1.90, 95% CI (-1.96, -1.85), *p*＜0.00001; heterogeneity: χ^2^ =1.67, *I^2^* =0%).  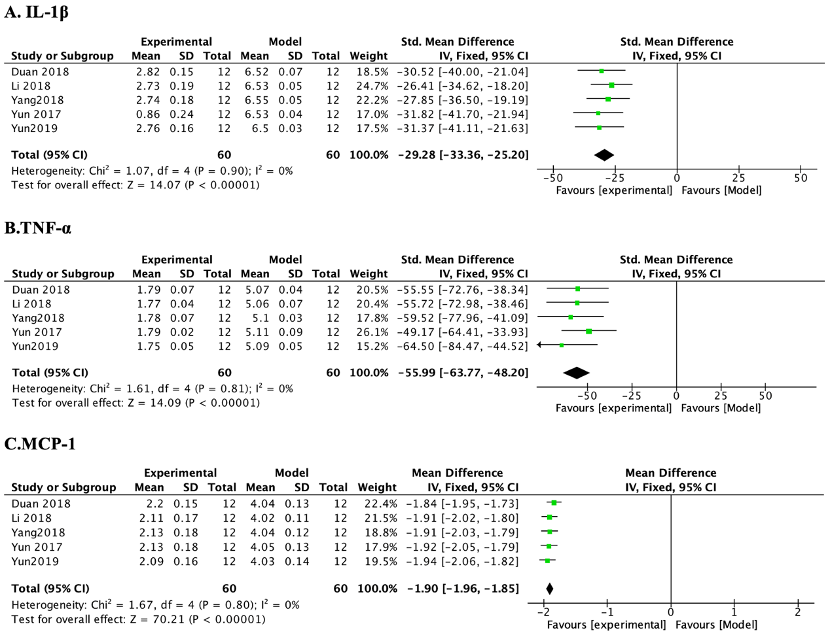  ***Fig .4. The forest plot: effects of PF for decreasing mRNA of IL-1β，TNF-α and MCP-1 compared with the model group.***  **Inhibition of macrophage activation**  **Protein expression of CD68**  Of the 9 studies, 6 studies used CD68 positive macrophages as the observation index. A meta-analysis of 6 studies indicated that paeoniflorin significantly reduced CD68 positive macrophage infiltration in tubulointerstitial (Table 4 and Fig.5-A) (n =144, SMD-3.72, 95% CI(-4.30, -3.14), *p*＜0.00001; heterogeneity: χ^2^ =3.45, *I^2^* = 0%) areas of diabetic nephropathy.  **mRNA of iNOS**  Of the 9 studies, 5 studies focused on the effect of PF treatment on iNOS mRNA expression. After sensitivity analysis, 1 study was removed separately because the modeling methods, animal species, doses of PF, and duration of treatment in this study were different from others. Subsequently, the heterogeneity of iNOS was decreased substantially (Table 4 and Fig. 5-B) (n = 96, SMD-33.05, 95% CI (-38.34, -27.75), *p* < 0.00001; heterogeneity: χ^2^ =9.84, *I^2^* = 69%).  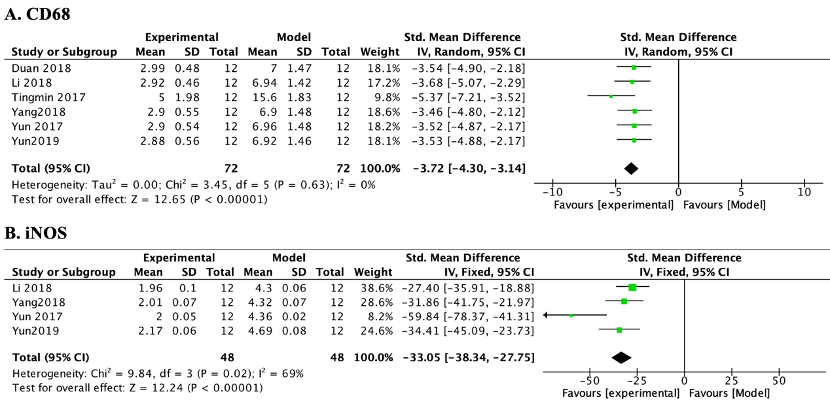  ***Fig. 5. The forest plot: effects of PF for decreasing mRNA of iNOS compared with the model group.***  ***Table 4. List of the anti-inflammatory, inhibition of macrophage activation effects of PF.***   \| Variables \| Experiments(n) \| Individuals  (n) \| SMD \| 95%CI \| P-value \| Heterogeneity \| \| --- \| --- \| --- \| --- \| --- \| --- \| --- \| \| **(1) Effect on inflammatory markers** \| \| \| \| \| \|  \| \| IL-1β \| 5 \| 120 \| -29.28 \| (-33.36, -25.20) \| *P* <0.00001 \| χ^2^ =1.07, *I^2^* = 0% \| \| TNF- α \| 5 \| 120 \| -55.99 \| (-63.77, -48.20) \| *P* <0.00001 \| χ^2^ =1.61, *I^2^* = 0% \| \| MCP-1 \| 5 \| 120 \| -1.90 \| (-1.96, -1.85) \| *P* <0.00001 \| χ^2^ =1.67, *I^2^* = 0% \| \| **(2) Effect on Macrophage infiltration** \| \| \| \| \| \|  \| \| CD68 \| 6 \| 144 \| -3.72 \| (-4.30, -3.14) \| *p*＜0.00001 \| χ^2^ =3.45, *I^2^* = 0% \| \| iNOS \| 4 \| 96 \| -33.0 \| (-38.34, -27.75) \| *p*＜0.00001 \| χ^2^ =9.84, *I^2^* = 69% \|   **Inhibition of signaling pathways and protein expression**  **TLR2 and TLR4 signaling pathway**  A meta-analysis across the 3 studies indicated that PF significantly reduced TLR2 levels compared with the model group (Table 5 and Fig.6-A) (n =72, SMD-12.32, 95% CI (-14.57, -10.07), *p*＜0.00001; heterogeneity: χ^2^ =0.02, *I^2^* = 0%). A meta-analysis across the 2 studies indicated that PF reduced TLR4 levels compared with the model group (Table 5 and Fig.6-B) (n =48, SMD-10.19, 95% CI (-13.61, -6.78), *p*＜0.00001; heterogeneity: χ^2^ =2.09, *I^2^* = 52%).  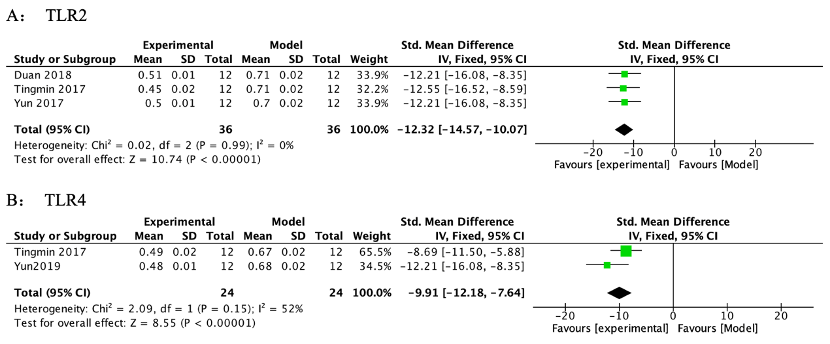  ***Fig. 6. The forest plot: effects of PF for decreasing TLR2 and TLR4 compared with the model group.***  **Downstream signal factors -Protein expression of MyD88, p-IRAK1, Trif, and p-IRF3**  A meta-analysis across 4 studies indicated that PF reduced MyD88 and p-IRAK1 protein expressions compared to controls. The sensitivity of MyD88 and p-IRAK1 was analyzed. The heterogeneity of MyD88 (Table 5 and Fig. 7-A) (n = 96, SMD-15.02, 95% CI (-21.07, -8.96), *p* < 0.00001; heterogeneity: χ^2^ =22.28, *I^2^* = 87%) did not decrease significantly after elimination of any studies. After sensitivity analysis, 1 study was removed separately due to that the modeling methods, different animal species, high and low PF dose and length of treatment duration in this study were different from others. Subsequently, the heterogeneity of p-IRAK1 was decreased substantially (Table 5 and Fig. 7-B) (n = 72, SMD-4.82, 95% CI (-5.80, -3.83), *p* < 0.00001; heterogeneity: χ^2^ =1.92, *I^2^* = 0%). A meta-analysis across 2 studies showed that the PF group had significantly reduced TRIF (Table 5 and Fig. 7-C) (n =72, SMD-8.57, 95% CI (-10.83, -6.31), *p* < 0.00001; heterogeneity: χ^2^ =3.87, *I^2^* = 48%) and p-IRF3 protein expressions (Table 5 and Fig. 7-D) (n = 72, SMD-8.60, 95% CI (-16.07, -1.13), *p* = 0.02; heterogeneity: χ^2^ =41.40, *I^2^* = 95%) compared with the model group (*p* < 0.001).  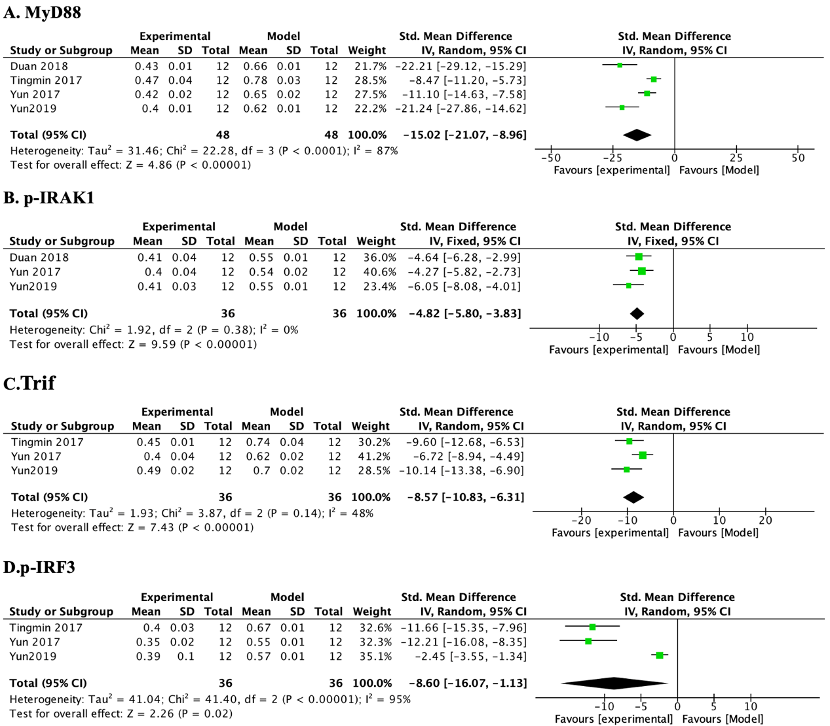  ***Fig. 7. The forest plot: effects of PF for decreasing MyD88,p-IRAK1,Trif and p-IRF3 compared with the model group.***  **JAK2/ STAT3 signal path**  A meta-analysis across the 2 studies indicated that PF significantly reduced glomerular (Table 5 and Fig.8-A) (n =48, SMD-6.17, 95% CI (-6.17, -4.71), p＜0.00001; heterogeneity: χ2 =0.28, I2 = 0%) and tubulointerstitial (Table 5 and Fig.8-B) (n =48, SMD-4.35, 95% CI (-5.46, -3.24), p＜0.00001; heterogeneity: χ2 =0.06, I2 = 0%) P-JAK2 protein levels compared with controls. Also compared with the model group, PF obviously reduced the levels of tubulointerstitial p-STAT3 protein (Table 5 and Fig.8-C) (n =48, SMD-8.26, 95% CI (-10.15, -6.36), p＜0.00001; heterogeneity: χ2 =0.16, I2 = 0%) but had no obvious difference in glomerular p-STAT3 protein (Table 5 and Fig.8-D) (n =48, SMD-0.20, 95% CI (-0.76, 0.37), p=0.95; heterogeneity: χ2 =0, I2 = 0%).  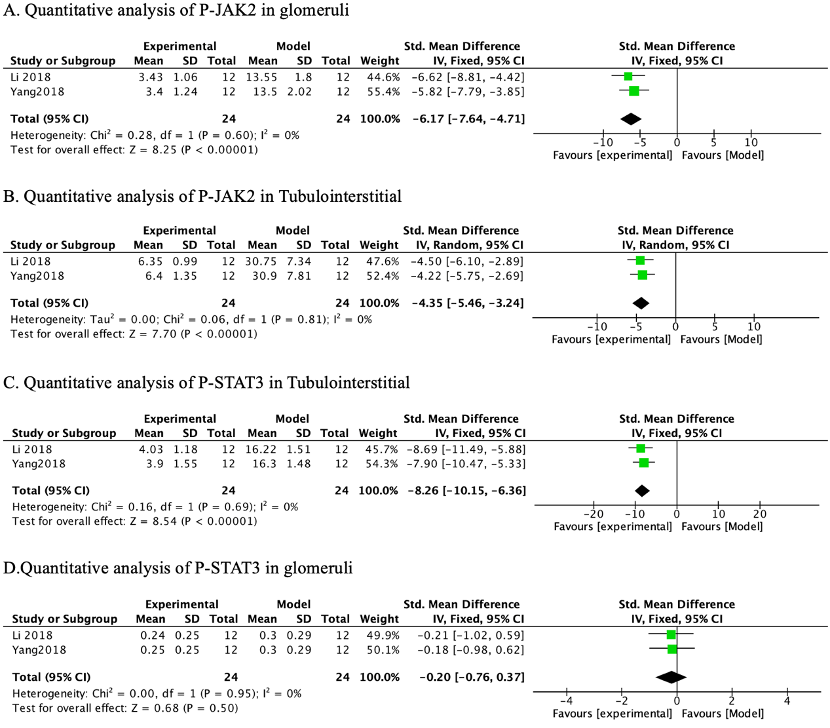  ***Fig. 8. The forest plot: effects of PF for decreasing P-JAK2 and P-STAT3 compared with the model group.***  **Protein expression of NF-κB p-p65, NF-κB p65**  A meta-analysis across 5 studies indicated that PF reduced NF-κB-p65 protein expressions compared to controls. After sensitivity analysis, 1 study was removed separately because the modeling methods, doses of PF, animal species and duration of treatment in this study were varied from others. Subsequently, the heterogeneity of NF-κB-p65 was decreased substantially (Table 5 and Fig. 9-A) (n = 92, SMD-7.87, 95% CI (-9.21, -6.53), *p* < 0.00001; heterogeneity: χ^2^ =5.46, I^2^ = 45%). A meta-analysis across 4 studies revealed that PF reduced NF-κB-p-p65 protein expression compared to controls. After sensitivity analysis, 1 study was removed separately due to that the animal species, modeling methods, doses of PF, and duration of treatment in this study were different from others. Subsequently, the heterogeneity of NF-κB-p-p65 was decreased substantially (Table 5 and Fig. 9-B) (n = 72, SMD-33.16, 95% CI (-39.11, -27.20), *p* < 0.00001; heterogeneity: χ^2^ =0.43, I^2^ = 0%).  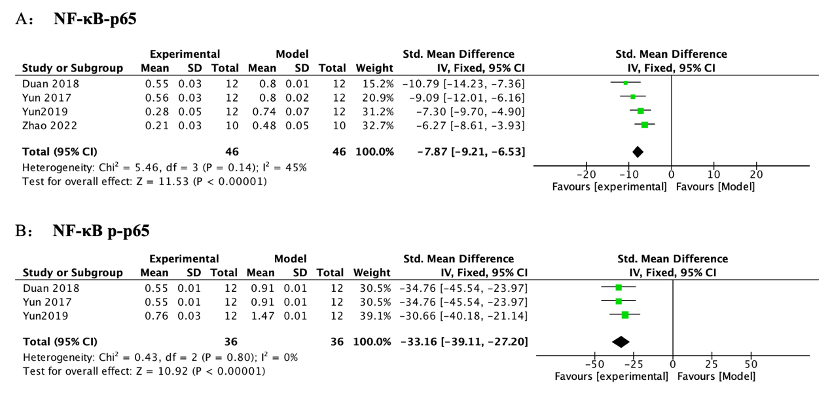  ***Fig. 9. The forest plot: effects of PF for decreasing NF-κB-p65 and NF-κB p-p65 compared with the model group.***  ***Table 5. List of*** ***the inhibition of signaling pathways and protein expression effects of PF.***   \| Variables \| Experiments(n) \| \| Individuals  (n) \| SMD \| 95%CI \| P-value \| Heterogeneity \| \| --- \| --- \| --- \| --- \| --- \| --- \| --- \| --- \| \| **(1) Effect onTLR2 and TLR4 signaling pathway** \| \| \| \| \| \| \| \| \| TLR2 \| \| 3 \| 72 \| -12.32 \| (-14.57, -10.07) \| *P*<0.00001 \| χ^2^ =0.02, *I^2^* = 0% \| \| TLR4 \| \| 2 \| 48 \| 10.19 \| (-13.61, -6.78) \| *P*<0.00001 \| χ^2^ =2.09, *I^2^* = 52% \| \| **(2) Effect on Downstream signal factors** \| \| \| \| \| \| \| \| \| MyD88 \| \| 4 \| 96 \| -15.02 \| (-21.07, -8.96) \| *P* <0.00001 \| χ^2^ =22.28, *I^2^* = 87% \| \| p-IRAK1 \| \| 3 \| 72 \| -4.82 \| (-5.80, -3.83) \| *P* <0.00001 \| χ^2^ =1.92, *I^2^* = 0% \| \| Trif \| \| 3 \| 72 \| -8.57 \| (-10.83, -6.31) \| *P* <0.00001 \| χ^2^ =3.87, *I^2^* = 48% \| \| p-IRF3 \| \| 3 \| 72 \| -8.60 \| (-16.07, -1.13) \| *P* <0.00001 \| χ^2^ =41.40, *I^2^* = 95% \| \| **(3) Effect on JAK2/ STAT3 signaling pathway** \| \| \| \| \| \| \| \| \| P-JAK2 in G \| \| 2 \| 48 \| -6.17 \| (-6.17, -4.71) \| *P* <0.00001 \| χ^2^ =0.28, *I^2^* = 0% \| \| P-JAK2 in T \| \| 2 \| 48 \| -4.35 \| (-5.46, -3.24) \| *P* <0.00001 \| χ^2^ =0.06, *I^2^* = 0% \| \| p-STAT3 in G \| \| 2 \| 48 \| -0.20 \| (-0.76, 0.37) \| *p*=0.95 \| χ^2^ =0, *I^2^* = 0% \| \| p-STAT3 in T \| \| 2 \| 48 \| -8.26 \| (-10.15, -6.36) \| *P* <0.00001 \| χ^2^ =0.16, *I^2^* = 0% \| \| **(4) Effect on NF-κB signaling pathway** \| \| \| \| \| \| \| \| \| NF-κB p65 \| \| 4 \| 92 \| -7.87 \| (-9.21, -6.53) \| *P* <0.00001 \| χ^2^ =5.46, *I^2^* = 45% \| \| NF-κB p-p65 \| \| 3 \| 72 \| -33.16 \| (-39.11, -27.20) \| *P* <0.00001 \| χ^2^ =0.43, *I^2^* = 0% \| |  |
|  | 20c | **Subgroup Analysis**  We explored potential confounders (including modeling method, animal species, PF dose, and treatment duration) that may increase 24-hour urinary protein heterogeneity and performed subgroup analyses of these factors to explore sources of heterogeneity（Table 6）. In the subgroup analysis of different animal species, we can see that the group of DN induced by C57BL / 6J mice（Table 6 and Fig.11-A）(n =136, SMD-4.89, 95% CI (-5.87, -3.91, p＜0.00001; heterogeneity: χ2 =104.22, I2 = 95%) is more heterogeneous than SD rats（Table 6 and Fig.11-A）(n =40, SMD-4.47, 95% CI (-5.75, -3.19, p＜0.00001; heterogeneity: χ2 =2.18, I2 = 54%).Before analyzing the subgroups of various modeling methods, to reduce sensitivity, we deleted the literature^[23]^ because it used the db/db diabetes mouse model and did not mention the relevant modeling reagent selection. In the subgroup analysis of different methods of modeling, we can see that the group of DN induced by STZ 50mg/kg（Table 6 and Fig.11-B）(n =120, SMD-12.48, 95% CI (-14.26, -10.69, p＜0.00001; heterogeneity: χ2 =4.80, I2 = 17%) is less heterogeneous than ＞STZ 50mg/kg in mice（Table 6 and Fig.11-B）(n =56, SMD-2.92, 95% CI ( -3.78, -2.05, p＜0.00001; heterogeneity: χ2 =12.60, I2 = 84%). Before analyzing the subgroup of varying PF doses, we deleted the literature (Huang et al., 2020) because it uses a derivative of paeoniflorin (CP-25) to reduce sensitivity. In the subgroup analysis of PF dose, we could see that the heterogeneity was not obviously different between the PF ＝100mg/kg（Table 6 and Fig.12-A）(n =140, SMD-11.34, 95% CI (-15.92, -6.76, *p*＜0.00001; heterogeneity: χ^2^ =56.14, *I^2^* = 91%) and PF＜100mg/kg groups（Table 6 and Fig.12-A）(n =60, SMD-5.48, 95% CI (-9.99, -0.98, p＝0.002; heterogeneity: χ2 =28.98, I2 = 93%).In the subgroup analysis of duration of treatment, we could see that the group with treatment time 12 weeks（Table 6 and Fig.12-B）(n =120, SMD-12.48, 95% CI ( -14.26, -10.69, p＜0.00001; heterogeneity: χ2 =4.80, I2 = 17%) had a significantly lower heterogeneity than the ＜ 12 weeks group（Table 6 and Fig.12-B）(n =80, SMD-3.41, 95% CI (-4.24, -2.58, *p*＜0.00001; heterogeneity: χ^2^ =29.27, *I^2^* = 90%). Through the above subgroup analysis, we included various methods of modeling, different animal species, and duration of treatment. These potential confounders all may increase the heterogeneity of outcome measures.  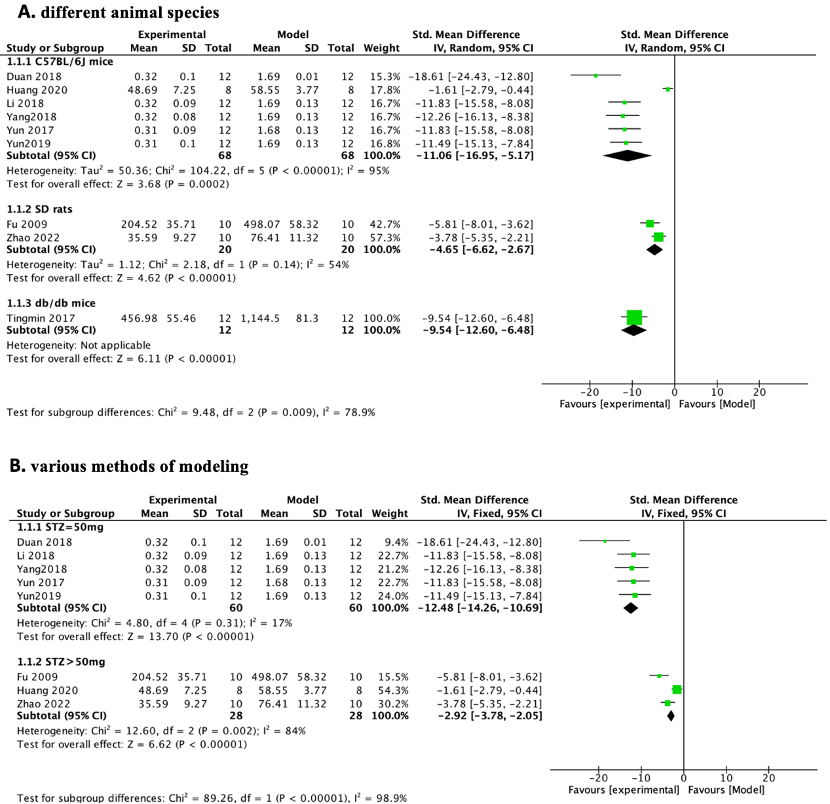  ***Fig. 11. Effect of PF on 24-h urinary protein in subgroups. (A) Induction type; (B) Species.***  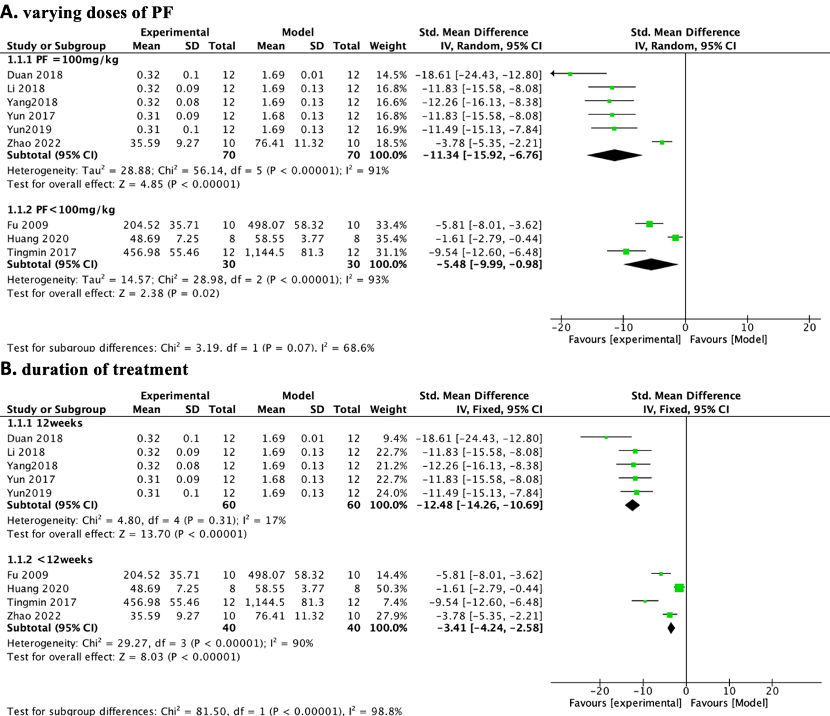  ***Fig.12. Effect of PF on 24-h urinary protein in subgroups. (A) PF dose; (B) Duration of treatment.***  ***Table 6. Stratified analysis of pooled estimates according to24-h urinary protein.***   \| Variables \| Experiments(n) \| Individuals  (n) \| SMD \| 95%CI \| P-value \| Heterogeneity \| \| --- \| --- \| --- \| --- \| --- \| --- \| --- \| \| **species** \|  \|  \|  \|  \|  \|  \| \| C57BL / 6J mice \| 6 \| 136 \| -4.89 \| (-5.87, -3.91) \| P<0.00001 \| χ^2^ =104.22, *I^2^* = 95% \| \| SD rats \| 2 \| 40 \| -4.47 \| (-5.75, -3.19) \| P<0.00001 \| χ^2^ =2.18, *I^2^* = 54% \| \| **modeling** \|  \|  \|  \|  \|  \|  \| \| STZ=50mg/kg \| 5 \| 120 \| -12.48 \| (-14.26, -10.69) \| P<0.00001 \| χ^2^ =4.80, *I^2^* = 17% \| \| STZ>50mg/kg \| 3 \| 56 \| -2.92 \| (-3.78, -2.05) \| P<0.00001 \| χ^2^ =12.60, *I^2^*= 84% \| \| **Dosage** \|  \|  \|  \|  \|  \|  \| \| PF =100mg/kg \| 6 \| 140 \| -11.34 \| (-15.92, -6.76) \| P<0.00001 \| χ^2^ =56.14, *I^2^*= 91% \| \| PF<100mg/kg \| 3 \| 60 \| -5.48 \| (-9.99, -0.98) \| P=0.002 \| χ^2^=28.98, *I^2^* = 93% \| \| **Period** \|  \|  \|  \|  \|  \|  \| \| 12weeks \| 5 \| 120 \| -12.48 \| (-14.26, -10.69) \| P<0.00001 \| χ^2^ =4.80, *I^2^*= 17% \| \| <12weeks \| 4 \| 80 \| -3.41 \| (-4.24, -2.58) \| P<0.00001 \| χ^2^ =29.27, *I^2^* = 90% \|   Consistently, the same confounding factors were revealed in another previous study with renal pathology as an experimental index. We re-screened the following potential confounding factors (including evaluation and grading of the glomerular mesangial expansion index and the tubulointerstitial damage index, tissue section thickness, and periodic acid-Schiff (PAS) staining method) through subgroup analysis of the renal pathology. In the subgroup analysis of tissue section thickness, 1 of the papers was removed because renal tissue section thickness was not mentioned. Contrasting the 2 µm thickness（Fig. 13-A）(n =48, SMD-3.17, 95% CI (-4.06, -2.27, *p*＜0.00001; heterogeneity: χ^2^ =0.15, *I^2^* = 0%)against the others（Fig. 13-A）(n =48 SMD-9.21, 95% CI (-11.30, -7.11, *p*＜0.00001; heterogeneity: χ^2^ =0.07, *I^2^* = 0%), the heterogeneity between the two groups was substantially reduced, respectively. In the subgroup analysis of the PAS staining method, 2 of these literatures were removed because they did not mention the specific method of PAS staining. Contrasting the potassium permanganate concentration (0.5%) （Fig. 13-B）(n =48 SMD-9.21, 95% CI (-11.30, -7.11, *p*＜0.00001; heterogeneity: χ^2^ =0.07, *I^2^* = 0%)in PAS staining with that of the two groups with potassium permanganate (1%), the heterogeneity of the potassium permanganate concentration (0.5%) was greatly reduced. Through the above subgroup analysis, we considered that the slice thickness of tissues, the drug concentration ratio of specific experimental procedures (e.g., PAS, HE, Masson) during the experiment and these potential confounding factors may all increase the heterogeneity of outcome measurement.  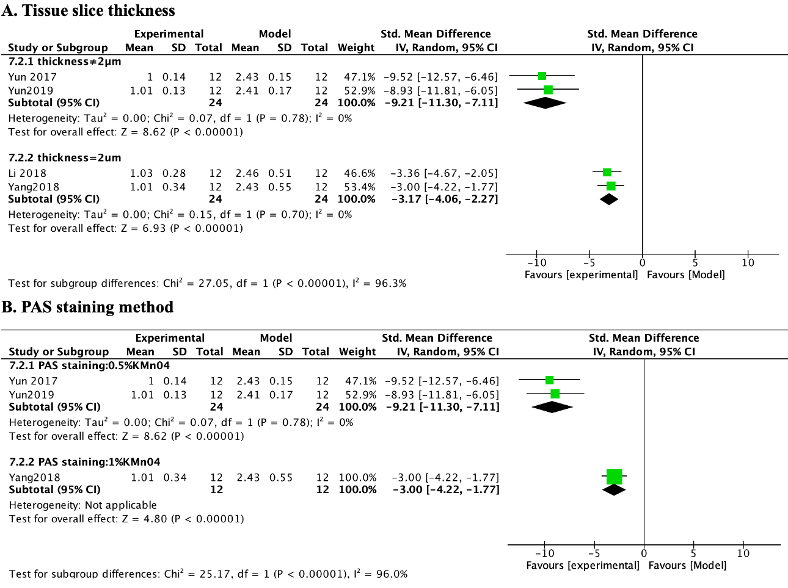  ***Fig. 13. \| Effect of PF on Renal Pathology in subgroups. (A) thickness; (B) PAS staining.*** |  |
|  | 20d | We explored potential confounders (including modeling method, animal species, PF dose, and treatment duration) that may increase 24-hour urinary protein heterogeneity and performed subgroup analyses of these factors to explore sources of heterogeneity（Table 6）. In the subgroup analysis of different animal species, we can see that the group of DN induced by C57BL / 6J mice（Table 6 and Fig.11-A）(n =136, SMD-4.89, 95% CI (-5.87, -3.91, p＜0.00001; heterogeneity: χ2 =104.22, I2 = 95%) is more heterogeneous than SD rats（Table 6 and Fig.11-A）(n =40, SMD-4.47, 95% CI (-5.75, -3.19, p＜0.00001; heterogeneity: χ2 =2.18, I2 = 54%).Before analyzing the subgroups of various modeling methods, to reduce sensitivity, we deleted the literature^[23]^ because it used the db/db diabetes mouse model and did not mention the relevant modeling reagent selection. In the subgroup analysis of different methods of modeling, we can see that the group of DN induced by STZ 50mg/kg（Table 6 and Fig.11-B）(n =120, SMD-12.48, 95% CI (-14.26, -10.69, p＜0.00001; heterogeneity: χ2 =4.80, I2 = 17%) is less heterogeneous than ＞STZ 50mg/kg in mice（Table 6 and Fig.11-B）(n =56, SMD-2.92, 95% CI ( -3.78, -2.05, p＜0.00001; heterogeneity: χ2 =12.60, I2 = 84%). Before analyzing the subgroup of varying PF doses, we deleted the literature (Huang et al., 2020) because it uses a derivative of paeoniflorin (CP-25) to reduce sensitivity. In the subgroup analysis of PF dose, we could see that the heterogeneity was not obviously different between the PF ＝100mg/kg（Table 6 and Fig.12-A）(n =140, SMD-11.34, 95% CI (-15.92, -6.76, *p*＜0.00001; heterogeneity: χ^2^ =56.14, *I^2^* = 91%) and PF＜100mg/kg groups（Table 6 and Fig.12-A）(n =60, SMD-5.48, 95% CI (-9.99, -0.98, p＝0.002; heterogeneity: χ2 =28.98, I2 = 93%).In the subgroup analysis of duration of treatment, we could see that the group with treatment time 12 weeks（Table 6 and Fig.12-B）(n =120, SMD-12.48, 95% CI ( -14.26, -10.69, p＜0.00001; heterogeneity: χ2 =4.80, I2 = 17%) had a significantly lower heterogeneity than the ＜ 12 weeks group（Table 6 and Fig.12-B）(n =80, SMD-3.41, 95% CI (-4.24, -2.58, *p*＜0.00001; heterogeneity: χ^2^ =29.27, *I^2^* = 90%). Through the above subgroup analysis, we included various methods of modeling, different animal species, and duration of treatment. These potential confounders all may increase the heterogeneity of outcome measures.  Consistently, the same confounding factors were revealed in another previous study with renal pathology as an experimental index. We re-screened the following potential confounding factors (including evaluation and grading of the glomerular mesangial expansion index and the tubulointerstitial damage index, tissue section thickness, and periodic acid-Schiff (PAS) staining method) through subgroup analysis of the renal pathology. In the subgroup analysis of tissue section thickness, 1 of the papers was removed because renal tissue section thickness was not mentioned. Contrasting the 2 µm thickness（Fig. 13-A）(n =48, SMD-3.17, 95% CI (-4.06, -2.27, *p*＜0.00001; heterogeneity: χ^2^ =0.15, *I^2^* = 0%)against the others（Fig. 13-A）(n =48 SMD-9.21, 95% CI (-11.30, -7.11, *p*＜0.00001; heterogeneity: χ^2^ =0.07, *I^2^* = 0%), the heterogeneity between the two groups was substantially reduced, respectively. In the subgroup analysis of the PAS staining method, 2 of these literatures were removed because they did not mention the specific method of PAS staining. Contrasting the potassium permanganate concentration (0.5%) （Fig. 13-B）(n =48 SMD-9.21, 95% CI (-11.30, -7.11, *p*＜0.00001; heterogeneity: χ^2^ =0.07, *I^2^* = 0%)in PAS staining with that of the two groups with potassium permanganate (1%), the heterogeneity of the potassium permanganate concentration (0.5%) was greatly reduced. Through the above subgroup analysis, we considered that the slice thickness of tissues, the drug concentration ratio of specific experimental procedures (e.g., PAS, HE, Masson) during the experiment and these potential confounding factors may all increase the heterogeneity of outcome measurement. |  |
| Reporting biases | 21 | We explored potential confounders (including modeling method, animal species, PF dose, and treatment duration) that may increase 24-hour urinary protein heterogeneity and performed subgroup analyses of these factors to explore sources of heterogeneity（Table 6）. In the subgroup analysis of different animal species, we can see that the group of DN induced by C57BL / 6J mice（Table 6 and Fig.11-A）(n =136, SMD-4.89, 95% CI (-5.87, -3.91, p＜0.00001; heterogeneity: χ2 =104.22, I2 = 95%) is more heterogeneous than SD rats（Table 6 and Fig.11-A）(n =40, SMD-4.47, 95% CI (-5.75, -3.19, p＜0.00001; heterogeneity: χ2 =2.18, I2 = 54%).Before analyzing the subgroups of various modeling methods, to reduce sensitivity, we deleted the literature^[23]^ because it used the db/db diabetes mouse model and did not mention the relevant modeling reagent selection. In the subgroup analysis of different methods of modeling, we can see that the group of DN induced by STZ 50mg/kg（Table 6 and Fig.11-B）(n =120, SMD-12.48, 95% CI (-14.26, -10.69, p＜0.00001; heterogeneity: χ2 =4.80, I2 = 17%) is less heterogeneous than ＞STZ 50mg/kg in mice（Table 6 and Fig.11-B）(n =56, SMD-2.92, 95% CI ( -3.78, -2.05, p＜0.00001; heterogeneity: χ2 =12.60, I2 = 84%). Before analyzing the subgroup of varying PF doses, we deleted the literature (Huang et al., 2020) because it uses a derivative of paeoniflorin (CP-25) to reduce sensitivity. In the subgroup analysis of PF dose, we could see that the heterogeneity was not obviously different between the PF ＝100mg/kg（Table 6 and Fig.12-A）(n =140, SMD-11.34, 95% CI (-15.92, -6.76, *p*＜0.00001; heterogeneity: χ^2^ =56.14, *I^2^* = 91%) and PF＜100mg/kg groups（Table 6 and Fig.12-A）(n =60, SMD-5.48, 95% CI (-9.99, -0.98, p＝0.002; heterogeneity: χ2 =28.98, I2 = 93%).In the subgroup analysis of duration of treatment, we could see that the group with treatment time 12 weeks（Table 6 and Fig.12-B）(n =120, SMD-12.48, 95% CI ( -14.26, -10.69, p＜0.00001; heterogeneity: χ2 =4.80, I2 = 17%) had a significantly lower heterogeneity than the ＜ 12 weeks group（Table 6 and Fig.12-B）(n =80, SMD-3.41, 95% CI (-4.24, -2.58, *p*＜0.00001; heterogeneity: χ^2^ =29.27, *I^2^* = 90%). Through the above subgroup analysis, we included various methods of modeling, different animal species, and duration of treatment. These potential confounders all may increase the heterogeneity of outcome measures.  Consistently, the same confounding factors were revealed in another previous study with renal pathology as an experimental index. We re-screened the following potential confounding factors (including evaluation and grading of the glomerular mesangial expansion index and the tubulointerstitial damage index, tissue section thickness, and periodic acid-Schiff (PAS) staining method) through subgroup analysis of the renal pathology. In the subgroup analysis of tissue section thickness, 1 of the papers was removed because renal tissue section thickness was not mentioned. Contrasting the 2 µm thickness（Fig. 13-A）(n =48, SMD-3.17, 95% CI (-4.06, -2.27, *p*＜0.00001; heterogeneity: χ^2^ =0.15, *I^2^* = 0%)against the others（Fig. 13-A）(n =48 SMD-9.21, 95% CI (-11.30, -7.11, *p*＜0.00001; heterogeneity: χ^2^ =0.07, *I^2^* = 0%), the heterogeneity between the two groups was substantially reduced, respectively. In the subgroup analysis of the PAS staining method, 2 of these literatures were removed because they did not mention the specific method of PAS staining. Contrasting the potassium permanganate concentration (0.5%) （Fig. 13-B）(n =48 SMD-9.21, 95% CI (-11.30, -7.11, *p*＜0.00001; heterogeneity: χ^2^ =0.07, *I^2^* = 0%)in PAS staining with that of the two groups with potassium permanganate (1%), the heterogeneity of the potassium permanganate concentration (0.5%) was greatly reduced. Through the above subgroup analysis, we considered that the slice thickness of tissues, the drug concentration ratio of specific experimental procedures (e.g., PAS, HE, Masson) during the experiment and these potential confounding factors may all increase the heterogeneity of outcome measurement. |  |
| Certainty of evidence | 22 | The certainty (or confidence) in the body of evidence for each outcome assessed is currently assessed. It has been mentioned earlier. |  |
| **DISCUSSION** | | |  |
| Discussion | 23a | This is the first preclinical systematic review to evaluate the efficacy of PF for animal models of DN which included 9 studies with 526 animals. The quality of studies included was generally moderate. The above studies suggest that PF is a multifaceted reno-protective agent for the treatment of DN.The main mechanism of PF in the treatment of DN may be through the inhibition of the TLR2 / 4 signaling pathway under high glucose stimulation to initiate macrophage activation I. Its therapeutic effects are not only related to regulation of inflammatory pathways (e.g., MyD88 and NF- κ B pathway), inflammatory cytokines (e.gIL- α、 IL-1 β、 MCP-1), but also related to the regulation of antioxidant effects, including SOD and GSH PX (Fig.14).The possible mechanisms by which PF mediates reno-protection are summarized as follows: (1) Reduced mesangial expansion index and reduced tubulointerstitial injury index, and alleviated pathological damage to the kidney; (2) Inhibited macrophage to M1 type transformation; (3) Reduced the production of inflammatory factors (TNF- α, IL-1 β，MCP-1) and iNOS, a marker of macrophage activation; (4) Intervention on the expression of TLR2 / 4 and their downstream signaling pathways (MyD88,P-IRAK1, Trif, P-IRF3, NF- κ B p-p65, NF- κ B p65; (5) Significantly decreased p-JAK2 and p-STAT3 protein expression and inhibited JAK2 / STAT3 signaling pathway activation; (6) Increased SOD and GSH-Px activity to reduce the release of MDA, inhibit renal inflammatory responses, improve the body peroxidation status, thus alleviating the degree of renal tissue damage. |  |
|  | 23b | This meta-analysis has some limitations: (1) 9 studies did not report blind induction of models and were flawed in both blind assessment of outcomes and sample size calculation. The arrival guidelines were strictly followed for sample size estimation and blind assessment of outcomes in future animal studies; (2) The lack of relevant adverse PF outcomes reported, which may have led to an overestimation of the true effect of PF ; (3) Chronic hyperglycemia and hypertension are major risk factors for the development of DN, which is often accompanied by comorbidities such as hypertension and high blood lipids. Then the relevant factors such as hypertension and hyperlipidemia were not included in a modeling way in 8 animal models out of 9 studies. This may have biased the effectiveness of PF for DN treatment. In future animal studies, the clinic should be closely linked, and the effects caused by multiple factors should be comprehensively considered to minimize the possibility of bias;（4）In the absence of active intervention, DN on average will progress to ESRD within 6~7 years. The rate of renal function decline in DN varies among patients and is influenced by proteinuria, blood pressure, and blood glucose. Modeling in the 9 studies were all early diabetic nephropathy models. 2~3 of these studies focused on the ability of PF to effectively reduce BNU, and Ccr renal function-related clinical indicators. But this cannot illustrate that the performance of PF treatment is effective throughout DN development, so it is necessary for the future to design more rigorous experiments to ask about the value of PF in treating different stages of DN development. |  |
|  | 23c | This meta-analysis has some limitations: (1) 9 studies did not report blind induction of models and were flawed in both blind assessment of outcomes and sample size calculation. The arrival guidelines were strictly followed for sample size estimation and blind assessment of outcomes in future animal studies; (2) The lack of relevant adverse PF outcomes reported, which may have led to an overestimation of the true effect of PF ; (3) Chronic hyperglycemia and hypertension are major risk factors for the development of DN, which is often accompanied by comorbidities such as hypertension and high blood lipids. Then the relevant factors such as hypertension and hyperlipidemia were not included in a modeling way in 8 animal models out of 9 studies. This may have biased the effectiveness of PF for DN treatment. In future animal studies, the clinic should be closely linked, and the effects caused by multiple factors should be comprehensively considered to minimize the possibility of bias;（4）In the absence of active intervention, DN on average will progress to ESRD within 6~7 years. The rate of renal function decline in DN varies among patients and is influenced by proteinuria, blood pressure, and blood glucose. Modeling in the 9 studies were all early diabetic nephropathy models. 2~3 of these studies focused on the ability of PF to effectively reduce BNU, and Ccr renal function-related clinical indicators. But this cannot illustrate that the performance of PF treatment is effective throughout DN development, so it is necessary for the future to design more rigorous experiments to ask about the value of PF in treating different stages of DN development. |  |
|  | 23d | STZ is a highly selective islet β Cytotoxic agent, typically administered as a single high dose, resulting in complete β Cell necrosis. Different animals differ in their sensitivity to STZ. C57BL / 6 mice, like Wistar and SD rats, are exquisitely sensitive to STZ^[53]^. In fact, since the body size of the rat facilitates access to adequate renal tissue for monitoring renal physiological cases, studies are more inclined to select rats to establish diabetes models. STZ dose is one of the most important factors determining whether the DN model is successfully established. STZ may not successfully induce the expected diabetes model at lower doses, while at high doses (≥ 65 mg/kg) it may cause death, nephrotoxicity, or acute tubular necrosis in animals. It showed that a dose of 55 mg/kg STZ significantly reduced the above effects in rats. Our subgroup analysis of STZ dose showed a significant difference between the24-h urinary protein of > 50 mg/kg STZ and ≤ 50 mg/kg STZ. The inconsistent results may be because relatively high doses of STZ (≥ 50 mg/kg) caused nephrotoxicity, rather than hyperglycemia affecting24-h urinary protein. Since different types of mice are highly sensitive to STZ, experiments should strictly control the dosage of STZ to avoid high animal mortality and solve the problem of low modeling rate. (1) The protocol employed multiple administrations of low-dose STZ to model pathological outcomes similar to human T1DM with insulitis and insulin deficiency. (2) T Animals exposed to a high-fat diet were given an intermediate dose of STZ to reduce the β Cell capacity and, as a result, induce hyperglycemia associated with insulin resistance; (3) The suitable temperature and humidity were guaranteed throughout the modeling process. The animals had sufficient food and water before STZ injection, then the mice had to fast for 4 h, the rats fasted for 6 to 8 h, etc.  Although no obvious difference was found in the treatment between the PF high dose (100 mg/kg) and the PF low dose (60 mg/kg) in this study. But our subgroup analysis of the effect of PF treatment duration on 24-h urinary protein showed a clear difference between the 12 weeks treatment (n =120, SMD-16.38, 95% CI (-23.78, -8.99, *p*＜0.00001; heterogeneity: χ^2^ =58.56, *I^2^* = 93%)and < 12 weeks treatment groups(n =44, SMD-0.02, 95% CI (-0.61, 0.57, *p*=0.94; heterogeneity: χ^2^ =0, *I^2^* = 0%). This also suggests that PF treatment duration is most likely the source of heterogeneity. The heterogeneity produced by the length of oral PF exposure may be related to the absorption of PF in the body or the pharmacokinetics of the receptor binding. Numerous pharmacokinetic studies have shown that the bioavailability of PF is low. The bioavailability is around 3% to 4% after oral administration of PF in rats. This may be related to the low penetration rate of PF and the metabolic pathway. lipophilicity as well as p-glycoprotein-mediated eﬄux would affect PF's penetration rate in vivo. The metabolic pathways of PF are mainly the hydrolysis of ester bonds, glycosidic bonds and the conjugation with glucuronic acid. Studies have shown that PF is not metabolized by intestinal mucosal enzymes in the intestinal wall, as well as the degradation rate is extremely low in the liver and lung. In addition, oral bioavailability is a key factor to ensure that effective drug concentrations are achieved^[64]^. The low bioavailability of PF limits its clinical application due to its high hydrophilicity and low lipophilicity, low permeability, transporter efflux, and hydrolytic degradation in the intestinal lumen. The present study is still in the phase of exploring whether the drug is effective or not. The mechanism and results suggest that PF suppresses the immune and controls renal functions in DN. However, the differential relationship between high and low PF doses and efficacy remains uncertain, which may be related to the lower bioavailability of PF.Therefore, (1）We suggest that approaches to promote pf bioavailability should be explored in future experiments to explore the optimal dosage and therapeutic time frame of PF in the treatment of DN and expand the application of PF in the clinic. For example, accumulating evidence has confirmed that esterification of hydrophilic compounds can modulate the affinity of PF; (2) The bioavailability of PF can be increased by blocking efflux transporters, perhaps by using glucosidase inhibitors; (3) In addition, there are studies that found paeoniflorin-6 ′ - o-benzene sulfonate (cp-25) to have higher bioavailability as a derivative of paeoniflorin. Cp-25 exhibited a favorable absorption profile, low clearance, long mean residence time, and moderate in vivo bioavailability in rats. |  |
| **OTHER INFORMATION** | | |  |
| Registration and protocol | 24a | Registration PROSPERO number :CRD42022322110 |  |
|  | 24b | https://www.crd.york.ac.uk/PROSPERO/#myprospero |  |
|  | 24c | Change to systematic review modified primary outcome measure observations versus secondary outcome measure observations at registration. This is because pathological changes in diabetic nephropathy should be included as primary outcome measures, and we included downstream factors such as TLR2 / TLR4 as secondary outcome measures. |  |
| Support | 25 | This work was supported by the National Natural Science Foundation of China (No. 8207152547) and Sichuan Provincial Science (No.2021ZYD0089). |  |
| Competing interests | 26 | All authors declare no conflicts of interest. |  |
| Availability of data, code and other materials | 27 | Report the following are publicly available materials for the article: template data collection form; Data extracted from included studies; Was used for all data analyzed. |  |

*From:*  Page MJ, McKenzie JE, Bossuyt PM, Boutron I, Hoffmann TC, Mulrow CD, et al. The PRISMA 2020 statement: an updated guideline for reporting systematic reviews. BMJ 2021;372:n71. doi: 10.1136/bmj.n71

For more information, visit: <http://www.prisma-statement.org/>
