# Supplementary material for: Protective effect of paeoniflorin in diabetic nephropathy: A preclinical systematic review revealing the mechanism of action
Source: PLoS One. 2023 Sep 21;18(9):e0282275. doi: 10.1371/journal.pone.0282275 (PMC10513216; doi:10.1371/journal.pone.0282275)
Supplement: S1 File — (DOCX) [file pone.0282275.s002.docx]

**Supplementary materials**

***Appendix 1. Literature search strategy in Pubmed***

| **Database** | **Number** | **Search terms** |
| --- | --- | --- |
| **PubMed** | #1 | (Diabetic Nephropathy) OR (Nephropathies, Diabetic) OR (Nephropathy, Diabetic) OR (Diabetic Nephropathy) OR (Diabetic Kidney Disease) OR (Diabetic Kidney Diseases) OR (Kidney Disease, Diabetic) OR (Kidney Diseases, Diabetic) OR (Diabetic Glomerulosclerosis) OR (Glomerulosclerosis,Diabetic) OR (Intracapillary Glomerulosclerosis) OR (Nodular Glomerulosclerosis) OR (Glomerulosclerosis, Nodular) OR (Kimmelstiel-Wilson  Syndrome) OR (Kimmelstiel Wilson Syndrome) OR (Syndrome, Kimmelstiel-Wilson) OR (Kimmelstiel-Wilson Disease) OR (Kimmelstiel Wilson Disease) |
|  | #2 | (peoniflorin) OR (paeoniflorin) OR (peoniflorin sulfonate) OR (paeoniflorin-4-ethylether) OR (paeoniflorin-6'-O-benzenesulfonate) |
|  | #3 | #1 AND #2 |

***Appendix 2. Abbreviations***

| ***Abbreviation*** | ***Full Name*** |
| --- | --- |
| AUC | area under the curve |
| Bax | BCL2-Associated X |
| Bcl-2 | B-cell lymphoma-2 |
| BG | blood glucose |
| CVD | cardiovascular disease |
| CI | confidence interval |
| CP-25 | paeoniflorin-6 ′ - o-benzene sulfonate |
| Chol | cholesterol |
| CD68 | cluster of differentiation 68 |
| DN | diabetic nephropathy |
| DKD | diabetic kidney disease |
| DM1 | type 1 diabetes mellitus |
| DM2 | type 2 diabetes mellitus |
| ESRD | end-stage renal disease |
| FBG | fasting blood-glucose |
| FIN | fasting insulin level |
| GSH-Px | glutathione peroxidase |
| GRK2 | G protein coupled receptor kinases 2 |
| GFR | glomerular filtration rate |
| HOMA-IR | homeostasis model assessment of insulin resistance |
| iNOS | inducible nitric oxide synthase |
| ICAM1 | intercellular cell adhesion molecule 1 |
| INR | international normalized ratio |
| MCP-1 | monocyte chemoattractant protein 1 |
| KW | kidney weight |
| BW | body weight |
| IL-1β | Interleukin-1β |
| MyD88 | myeloid differentiation factor 88 |
| MD | mean difference |
| NF- κ B- p65 | nuclear factor kappa p65 |
| Nrf2 | nuclear factor erythroid-2 related factor 2 |
| p-IRS1 | phosphorylated-Insulin receptor substrate 1; |
| PF | paeoniflorin |
| p-JAK2 | phosphorylated-Janus kinase 2 |
| p-IRAK1 | phosphorylated interleukin-1 receptor-associated kinase |
| p-IRF3 | phosphorylated interferon regulatory factor 3 |
| p-STAT3 | phosphorylated signal transducer and activator of transcription 3 |
| SOD | superoxide dismutase |
| SMD | standardized mean difference |
| SIRT1 | silent information regulator factor 2-related enzyme 1 |
| SD | Sprague Dawley |
| SCR | serum creatinine |
| STZ | streptozotocin |
| TGP | total glucoside of peony |
| TLR2 | toll like receptors 2 |
| TGF-β1 | transforming growth factor-β1 |
| TG | triglyceride |
| TLR4 | toll like receptors 4 |
| TNF- α | tumor necrosis factor-α |
| TRIF | TIR domain-containing adaptor inducing interferon-β |
| UAlb | urinary albumin |
| UCR | urinary creatinine |

***Supplementary Table 1-4: List of the dose/time-effect between main DN index and PF.***

***Supplementary Table 1: List of the dose/time-effect between 24-h urinary protein and PF administration***

|  | **year** | **duration of treatment (W)** | **Dose(mg)** | **P** |
| --- | --- | --- | --- | --- |
| (Duan et al.) | 2018 | 12 | 25 | *P* < 0. 01 |
| (Duan et al.) | 2018 | 12 | 50 | *P* < 0. 01 |
| (Duan et al.) | 2018 | 12 | 100 | *P* < 0. 01 |
| (Li et al., n.d.) | 2018 | 12 | 25 | *P* < 0. 01 |
| (Li et al., n.d.) | 2018 | 12 | 50 | *P* < 0. 01 |
| (Li et al., n.d.) | 2018 | 12 | 100 | *P* < 0. 01 |
| (Yang et al.) | 2018 | 12 | 25 | *P* < 0. 01 |
| (Yang et al.) | 2018 | 12 | 50 | *P* < 0. 01 |
| (Yang et al.) | 2018 | 12 | 100 | *P* < 0. 01 |
| (Y. Shao et al.) | 2017 | 12 | 100 | *P* < 0. 01 |
| (Y. Shao et al.) | 2017 | 12 | 50 | *P* < 0. 01 |
| (Y. Shao et al.) | 2017 | 12 | 25 | *P* < 0. 01 |
| (Y. Shao et al.) | 2019 | 12 | 25 | *P* < 0. 01 |
| (Y. Shao et al.) | 2019 | 12 | 50 | *P* < 0. 01 |
| (Y. Shao et al.) | 2019 | 12 | 100 | *P* < 0. 01 |
| (T. Zhang et al.) | 2017 | 2 | 60 | *P* < 0. 01 |
| (T. Zhang et al.) | 2017 | 2 | 30 | *P* < 0. 01 |
| (T. Zhang et al.) | 2017 | 2 | 15 | *P* < 0. 01 |
| (Zhao et al.) | 2022 | 8 | 100 | *P* < 0. 01 |
| (Zhao et al.) | 2022 | 8 | 50 | *P* < 0. 01 |
| (Fu et al.) | 2009 | 8 | 20 | *P* < 0. 01 |
| (Fu et al.) | 2009 | 8 | 10 | *P* < 0. 01 |
| (Fu et al.) | 2009 | 8 | 5 | *P* < 0. 05 |
| (Huang et al.) | 2020 | 5 | 70 | *P* < 0. 05 |

***Supplementary Table 2: List of the dose/time-effect between*** ***the glomerular mesangial expansion index and PF administration***

| **Study** | **year** | **duration of treatment (W)** | **Dose(mg)** | **P** |
| --- | --- | --- | --- | --- |
| (Duan et al.) | 2018 | 12 | 25 | *P* < 0. 01 |
| (Duan et al.) | 2018 | 12 | 50 | *P* < 0. 01 |
| (Duan et al.) | 2018 | 12 | 100 | *P* < 0. 01 |
| (Y. Shao et al.) | 2017 | 12 | 100 | *P* < 0. 01 |
| (Y. Shao et al.) | 2017 | 12 | 50 | *P* < 0. 01 |
| (Y. Shao et al.) | 2017 | 12 | 25 | *P* < 0. 01 |
| (Y. Shao et al.) | 2019 | 12 | 25 | *P* < 0. 01 |
| (Y. Shao et al.) | 2019 | 12 | 50 | *P* < 0. 01 |
| (Y. Shao et al.) | 2019 | 12 | 100 | *P* < 0. 01 |
| (Li et al., n.d.) | 2018 | 12 | 25 | *P* < 0. 05 |
| (Li et al., n.d.) | 2018 | 12 | 50 | *P* < 0. 05 |
| (Li et al., n.d.) | 2018 | 12 | 100 | *P* < 0. 05 |
| (Yang et al.) | 2018 | 12 | 25 | *P* < 0. 05 |
| (Yang et al.) | 2018 | 12 | 50 | *P* < 0. 05 |
| (Yang et al.) | 2018 | 12 | 100 | *P* < 0. 05 |

***Supplementary Table 3: List of the dose/time-effect between*** t***he tubulointerstitial damage index and PF administration***

| **Study** | **year** | **duration of treatment (W)** | **Dose(mg)** | **P** |
| --- | --- | --- | --- | --- |
| (Duan et al.) | 2018 | 12 | 25 | *P* < 0. 01 |
| (Duan et al.) | 2018 | 12 | 50 | *P* < 0. 01 |
| (Duan et al.) | 2018 | 12 | 100 | *P* < 0. 01 |
| (Y. Shao et al.) | 2017 | 12 | 100 | *P* < 0. 01 |
| (Y. Shao et al.) | 2017 | 12 | 50 | *P* < 0. 01 |
| (Y. Shao et al.) | 2017 | 12 | 25 | *P* < 0. 01 |
| (Y. Shao et al.) | 2019 | 12 | 25 | *P* < 0. 01 |
| (Y. Shao et al.) | 2019 | 12 | 50 | *P* < 0. 01 |
| (Y. Shao et al.) | 2019 | 12 | 100 | *P* < 0. 01 |
| (Li et al., n.d.) | 2018 | 12 | 25 | *P* < 0. 05 |
| (Li et al., n.d.) | 2018 | 12 | 50 | *P* < 0. 05 |
| (Li et al., n.d.) | 2018 | 12 | 100 | *P* < 0. 05 |
| (Yang et al.) | 2018 | 12 | 25 | *P* < 0. 05 |
| (Yang et al.) | 2018 | 12 | 50 | *P* < 0. 05 |
| (Yang et al.) | 2018 | 12 | 100 | *P* < 0. 05 |

***Supplementary Table 4: List of the dose/time-effect between MCP-1 mRNA expressionand PF administration***

| **Study** | **year** | **duration of treatment (W)** | **Dose(mg)** | **P** |
| --- | --- | --- | --- | --- |
| (Duan et al.) | 2018 | 12 | 25 | *P* < 0. 01 |
| (Duan et al.) | 2018 | 12 | 50 | *P* < 0. 01 |
| (Duan et al.) | 2018 | 12 | 100 | *P* < 0. 01 |
| (Li et al., n.d.) | 2018 | 12 | 25 | *P* < 0. 01 |
| (Li et al., n.d.) | 2018 | 12 | 50 | *P* < 0. 01 |
| (Li et al., n.d.) | 2018 | 12 | 100 | *P* < 0. 01 |
| (Yang et al.) | 2018 | 12 | 25 | *P* < 0. 01 |
| (Yang et al.) | 2018 | 12 | 50 | *P* < 0. 01 |
| (Yang et al.) | 2018 | 12 | 100 | *P* < 0. 01 |
| (Y. Shao et al.) | 2017 | 12 | 100 | *P* < 0. 01 |
| (Y. Shao et al.) | 2017 | 12 | 50 | *P* < 0. 01 |
| (Y. Shao et al.) | 2017 | 12 | 25 | *P* < 0. 01 |
| (Y. Shao et al.) | 2019 | 12 | 25 | *P* < 0. 01 |
| (Y. Shao et al.) | 2019 | 12 | 50 | *P* < 0. 01 |
| (Y. Shao et al.) | 2019 | 12 | 100 | *P* < 0. 01 |
| (T. Zhang et al.) | 2017 | 2 | 60 | *P* < 0. 01 |
| (T. Zhang et al.) | 2017 | 2 | 30 | *P* < 0. 01 |
| (T. Zhang et al.) | 2017 | 2 | 15 | *P* < 0. 01 |
| (Fu et al.) | 2009 | 8 | 20 | *P* < 0. 01 |
| (Fu et al.) | 2009 | 8 | 10 | *P* < 0. 01 |
| (Fu et al.) | 2009 | 8 | 5 | *P* < 0. 01 |
| (Huang et al.) | 2020 | 5 | 70 | *P* < 0. 05 |

**
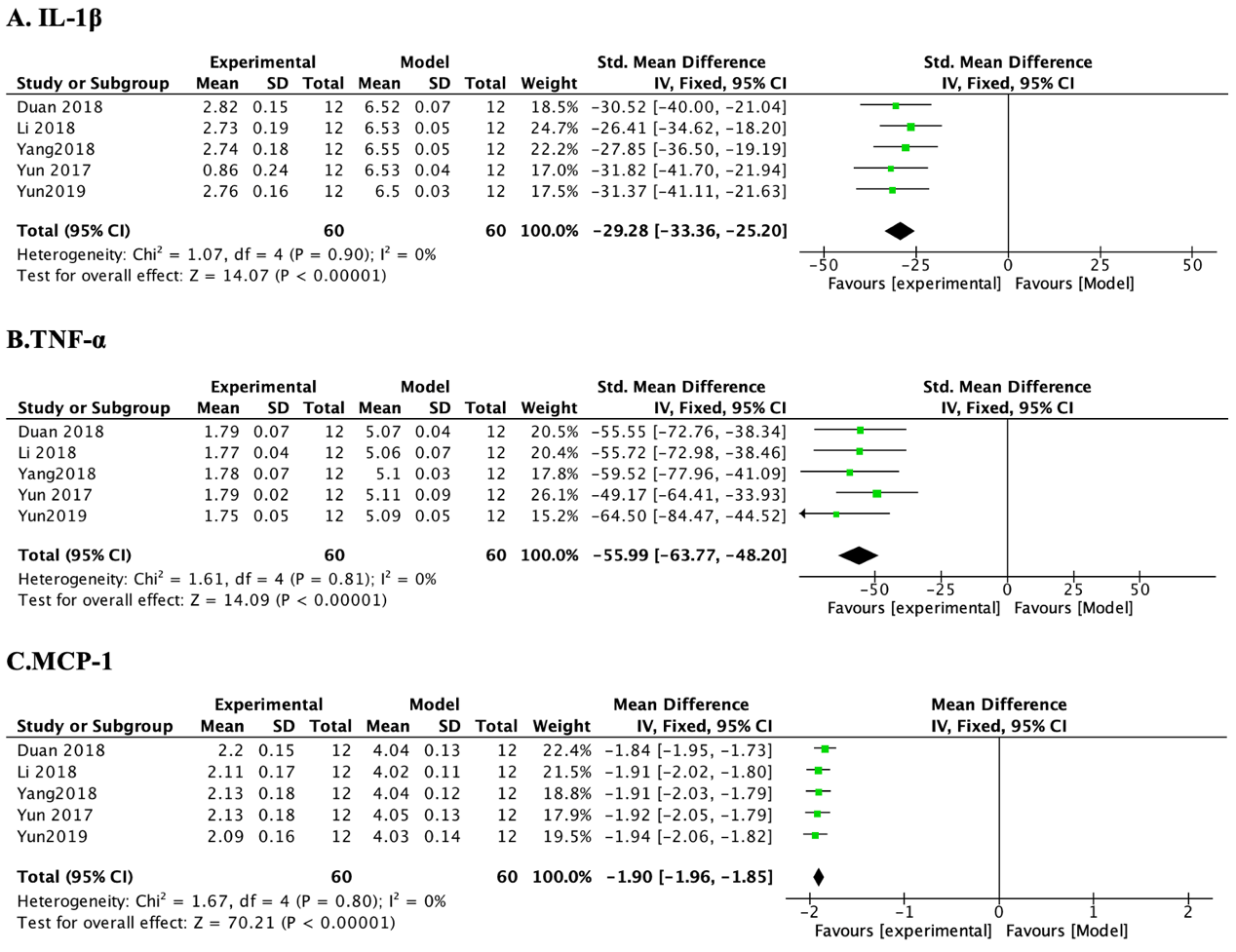
**

***Supplementary Figure 1. The forest plot: effects of PF for decreasing mRNA of IL-1β，TNF-α and MCP-1 compared with the model group.***

**
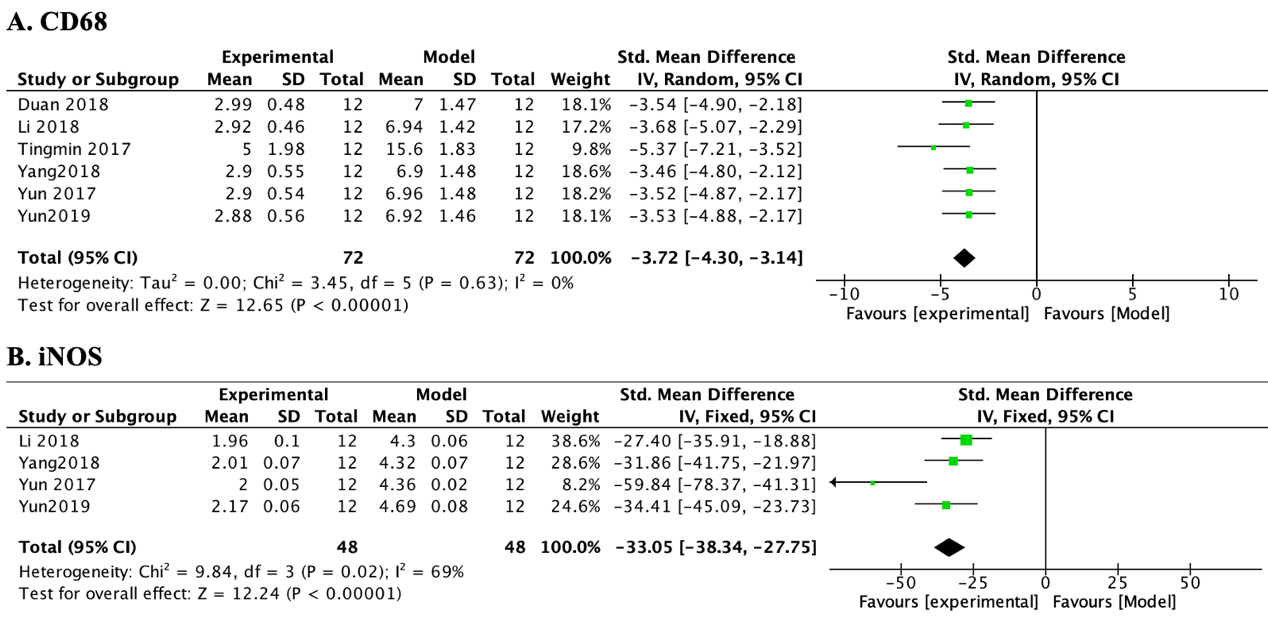
**

***Supplementary Figure 2. The forest plot: effects of PF for decreasing mRNA of iNOS compared with the model group.***

**
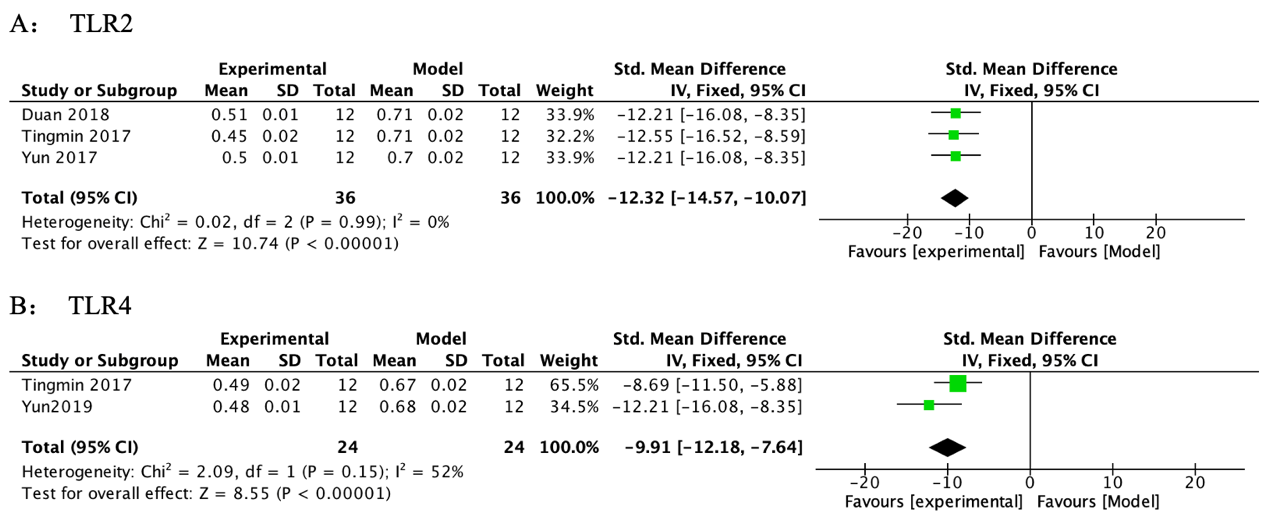
**

***Supplementary Figure 3. The forest plot: effects of PF for decreasing TLR2 and TLR4 compared with the model group.***

**
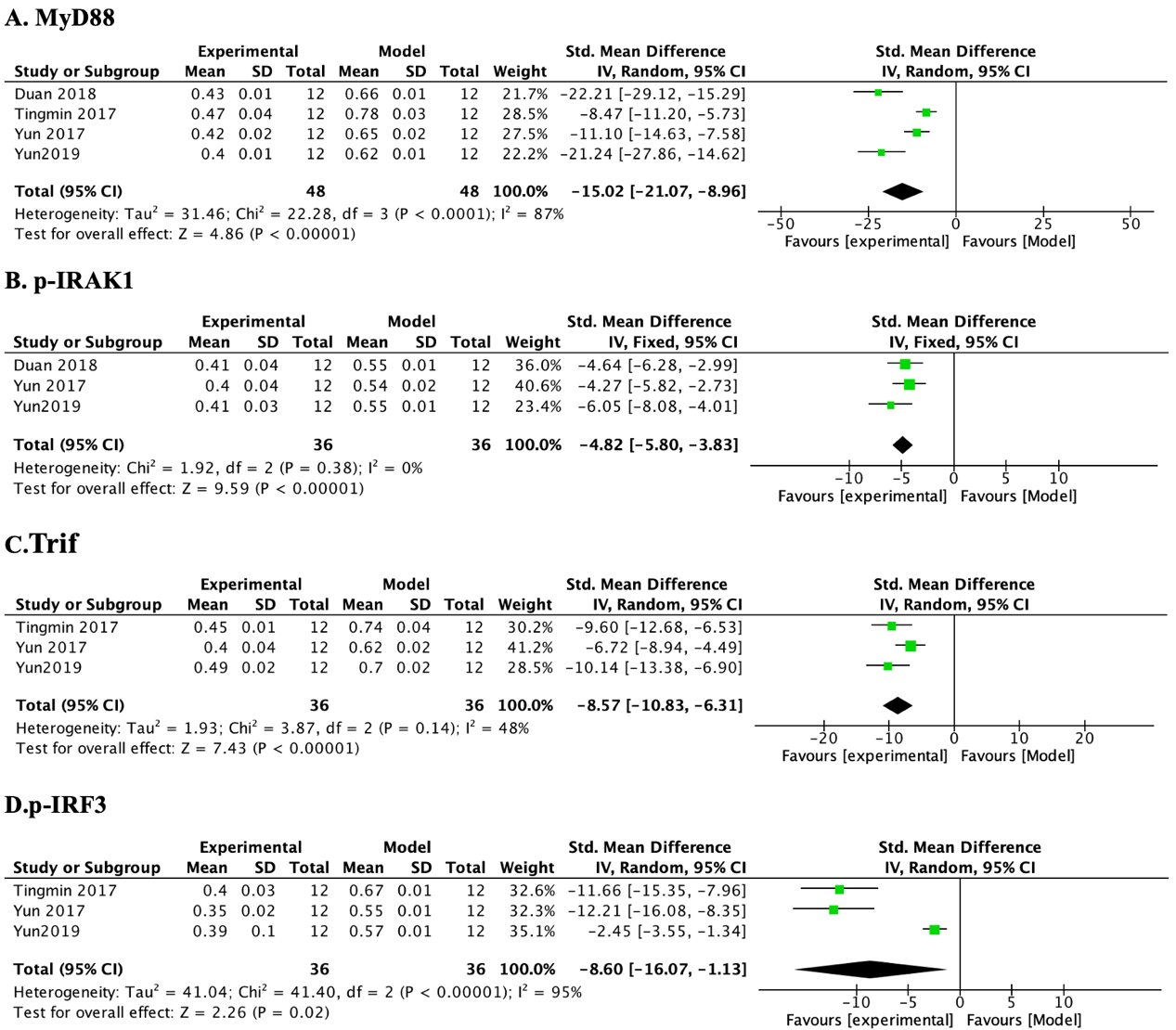
**

***Supplementary Figure 4. The forest plot: effects of PF for decreasing MyD88,p-IRAK1,Trif and p-IRF3 compared with the model group.***


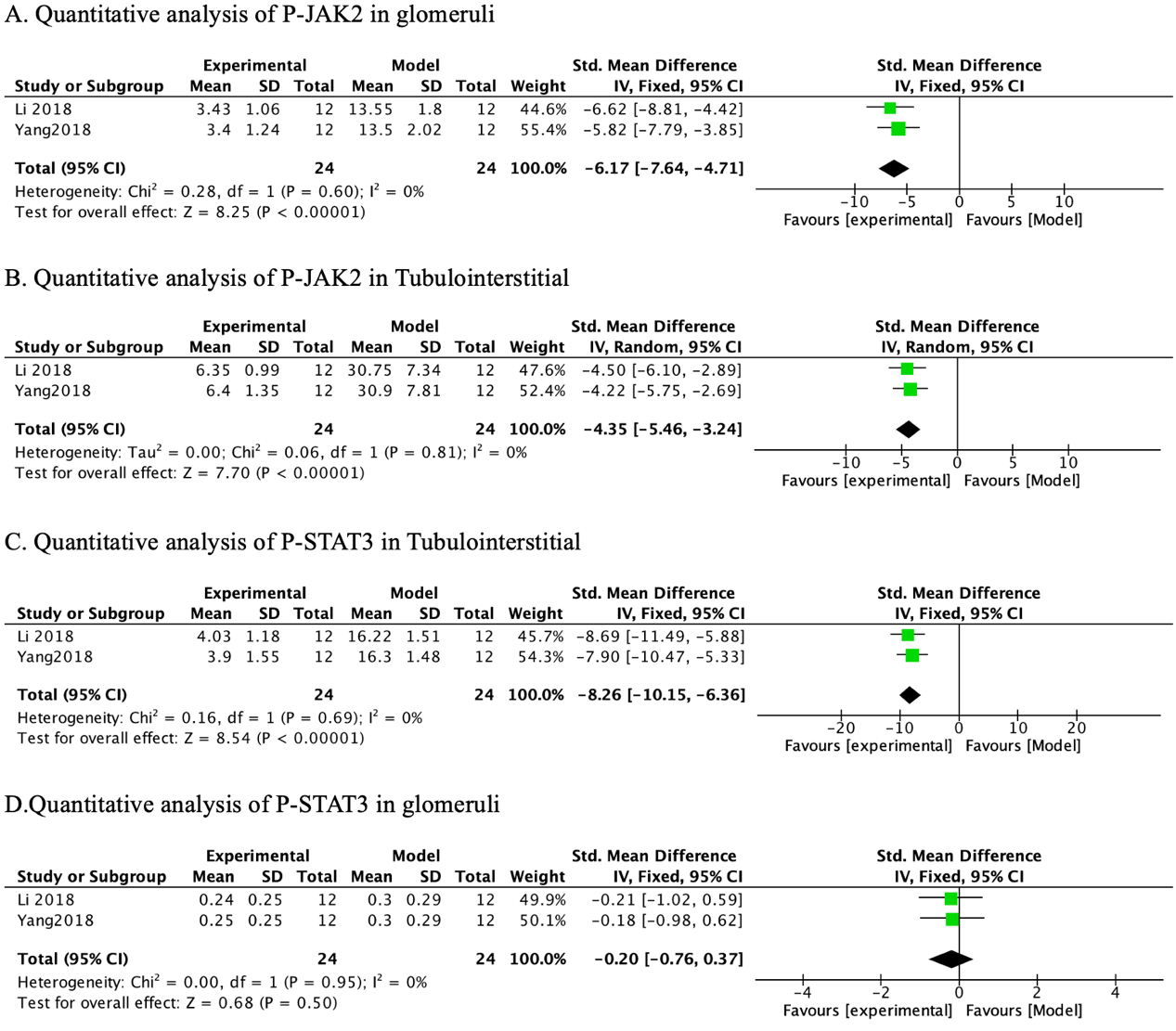


***Supplementary Figure 5. The forest plot: effects of PF for decreasing P-JAK2 and P-STAT3 compared with the model group.***


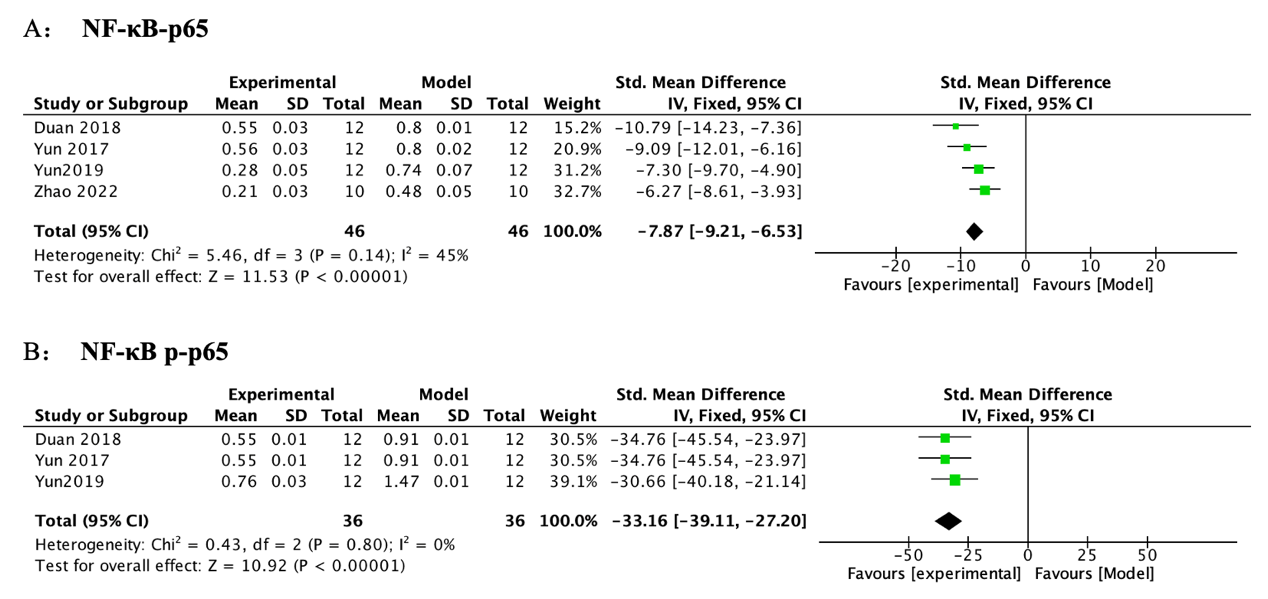


***Supplementary Figure 6. The forest plot: effects of PF for decreasing NF-κB-p65 and NF-κB p-p65 compared with the model group.***


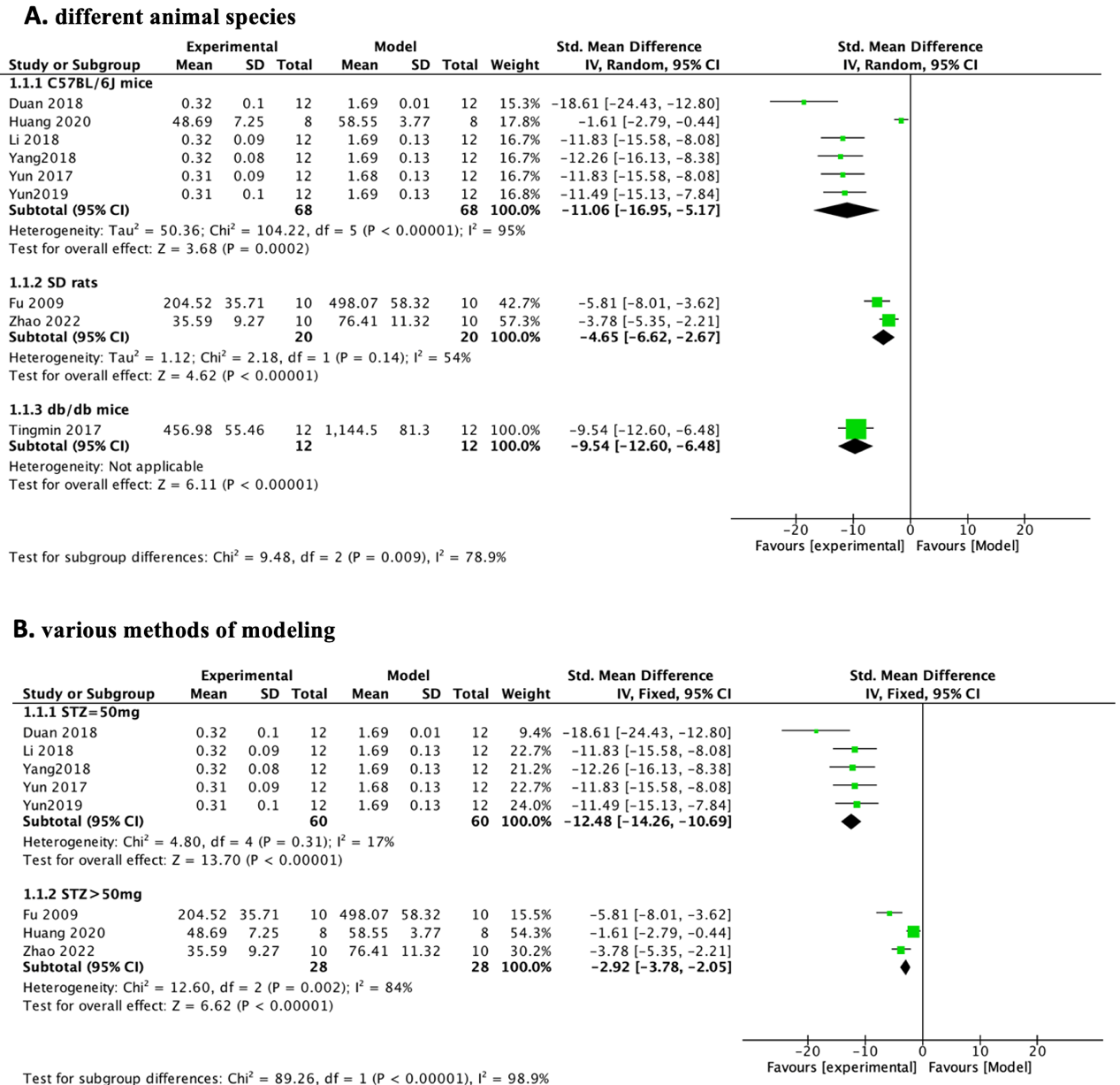


***Supplementary Figure 7. Effect of PF on 24-h urinary protein in subgroups. (A) Induction type; (B) Species.***


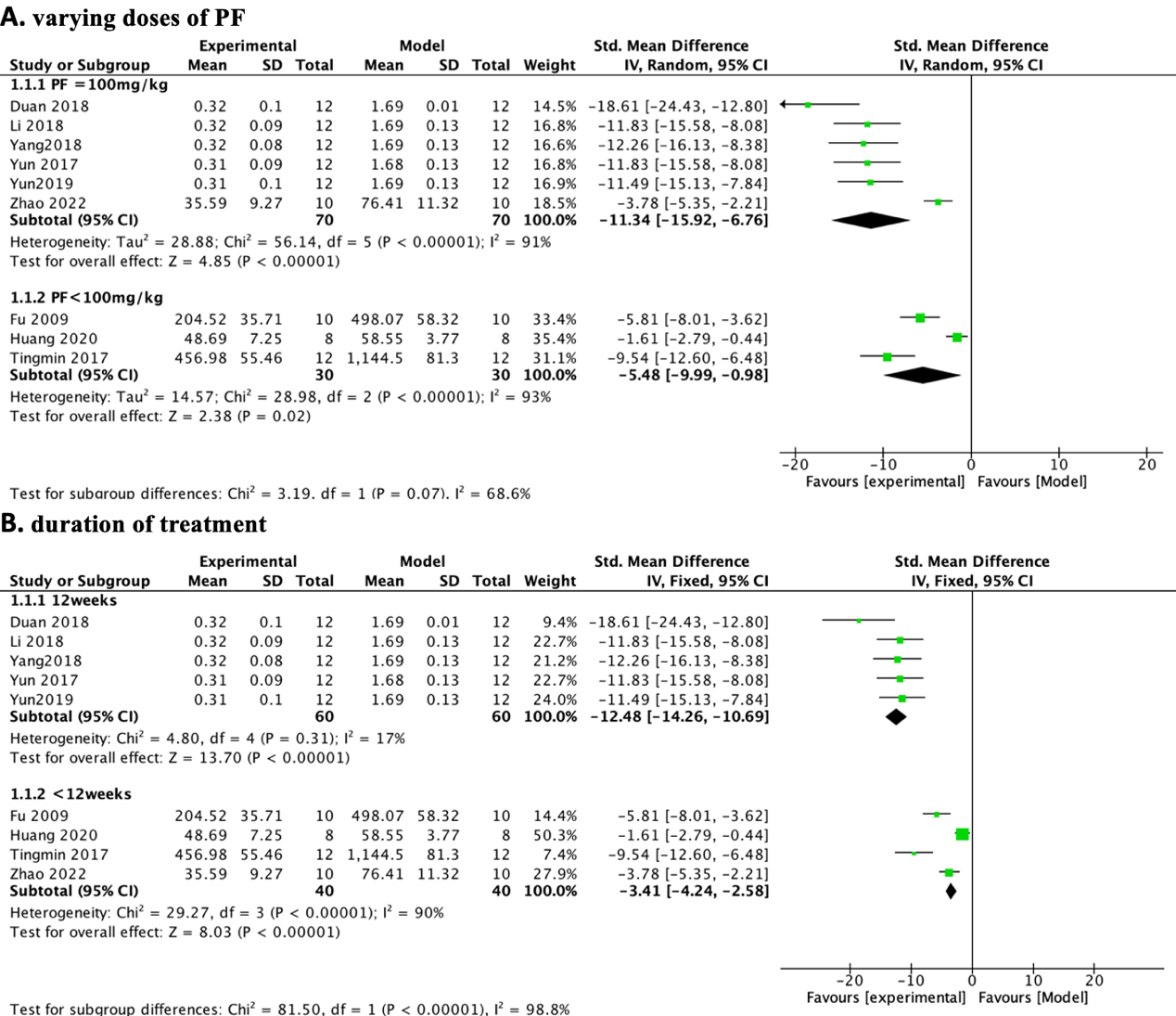


***Supplementary Figure 8. Effect of PF on 24-h urinary protein in subgroups. (A) PF dose; (B) Duration of treatment.***
